# Solar energy conversion using first row d-block metal coordination compound sensitizers and redox mediators

**DOI:** 10.1039/d1sc06828h

**Published:** 2022-01-05

**Authors:** Catherine E. Housecroft, Edwin C. Constable

**Affiliations:** Department of Chemistry, University of Basel Mattenstrasse 24a, BPR 1096 4058 Basel Switzerland catherine.housecroft@unibas.ch

## Abstract

The use of renewable energy is essential for the future of the Earth, and solar photons are the ultimate source of energy to satisfy the ever-increasing global energy demands. Photoconversion using dye-sensitized solar cells (DSCs) is becoming an established technology to contribute to the sustainable energy market, and among state-of-the art DSCs are those which rely on ruthenium(ii) sensitizers and the triiodide/iodide (I_3_^−^/I^−^) redox mediator. Ruthenium is a critical raw material, and in this review, we focus on the use of coordination complexes of the more abundant first row d-block metals, in particular copper, iron and zinc, as dyes in DSCs. A major challenge in these DSCs is an enhancement of their photoconversion efficiencies (PCEs) which currently lag significantly behind those containing ruthenium-based dyes. The redox mediator in a DSC is responsible for regenerating the ground state of the dye. Although the I_3_^−^/I^−^ couple has become an established redox shuttle, it has disadvantages: its redox potential limits the values of the open-circuit voltage (*V*_OC_) in the DSC and its use creates a corrosive chemical environment within the DSC which impacts upon the long-term stability of the cells. First row d-block metal coordination compounds, especially those containing cobalt, and copper, have come to the fore in the development of alternative redox mediators and we detail the progress in this field over the last decade, with particular attention to Cu^2+^/Cu^+^ redox mediators which, when coupled with appropriate dyes, have achieved *V*_OC_ values in excess of 1000 mV. We also draw attention to aspects of the recyclability of DSCs.

## Abstract 2

The progress over the last decade in the applications of first row d-block metal, especially iron, cobalt, copper and zinc, coordination compounds in redox shuttles and sensitizers in dye-sensitized solar cells is reviewed.

## Graphical abstract

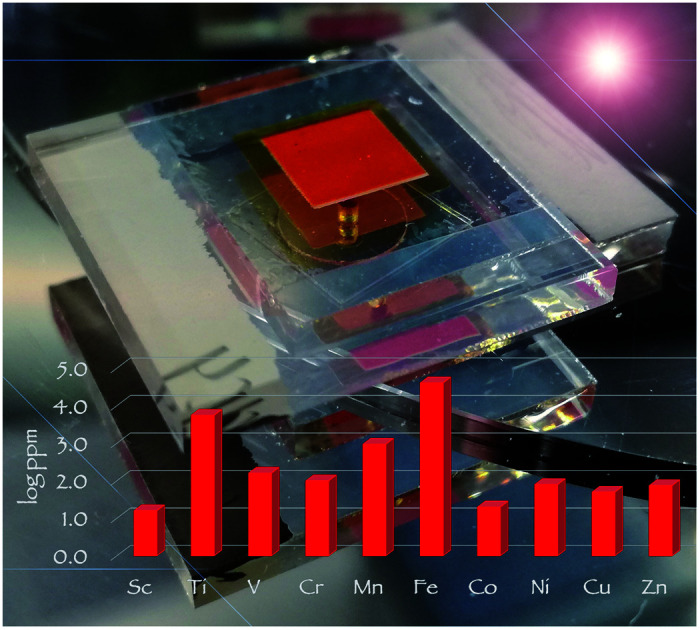

## Introduction

### Why solar energy?

The United Nations Member States adopted the 2030 Agenda for Sustainable Development in 2015. This recognizes seventeen sustainable development goals (SDGs), of which SDG7 has the aim to “ensure access to affordable, reliable, sustainable and modern energy for all” by 2030.^1^ Renewable energy incorporates biomass, wind, hydroelectric, solar and geothermal technologies. Because of their unlimited and cost-free supply, solar photons are an ideal source of energy to satisfy the ever-increasing global demands. Moreover, in contrast to fossil fuels, solar energy poses no direct threat to the environment.

**Fig. 1:**
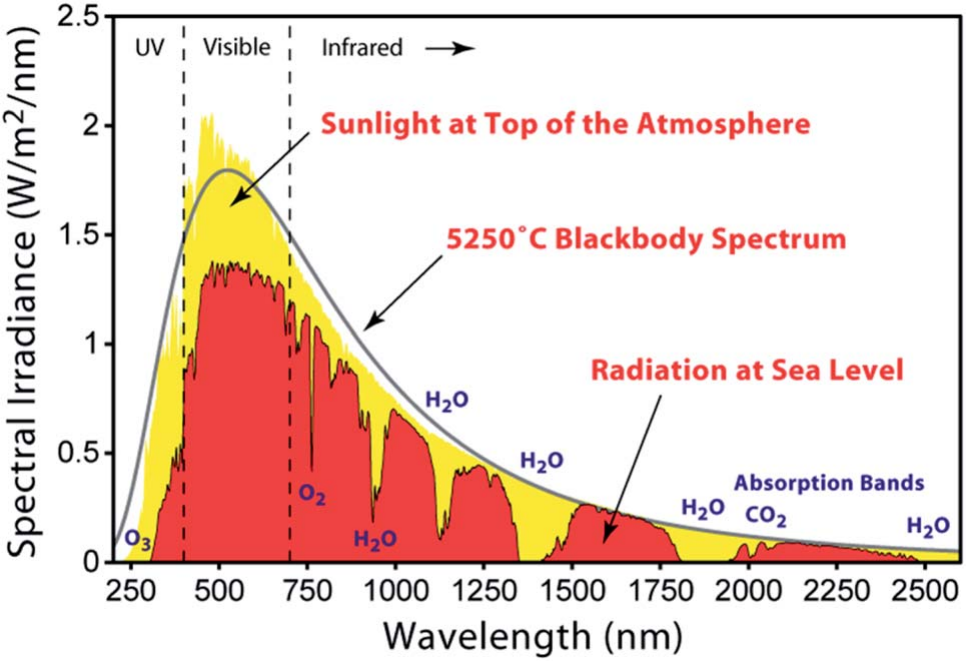
The air mass (AM) 1.5 solar spectrum [https://commons.wikimedia.org/wiki/File:Solar_spectrum_en.svg].

**Fig. 2:**
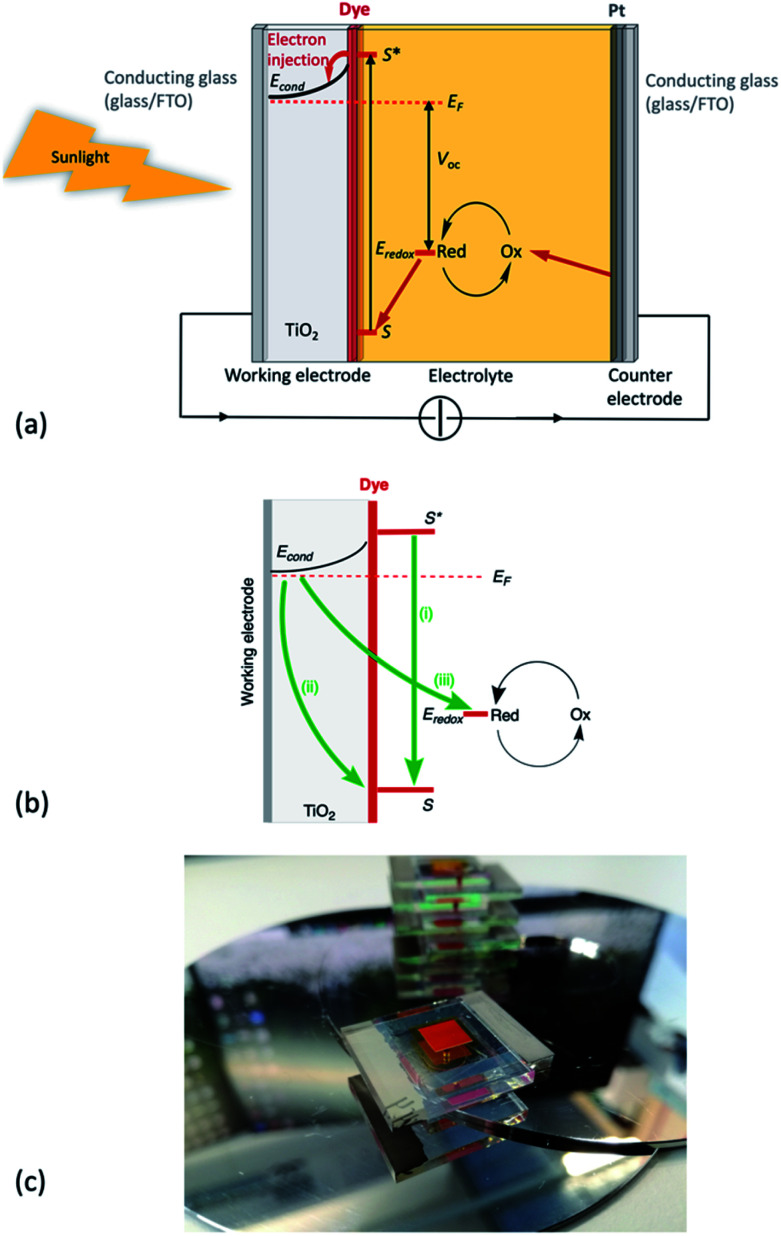
(a) A schematic representation of an n-type DSC. S = ground state of the dye; S* = excited state of the dye; *E*_F_ = Fermi level; *E*_cond_ = conduction band of the semiconductor; *E*_redox_ = redox potential of the redox shuttle (a component of the electrolyte); *V*_OC_ = open-circuit voltage. Working electrode = photoanode. The glass substrates may be replaced by polymer substrates. (b) Recombination processes: (i) decay of the excited state dye back to the ground state; (ii) recombination of the injected electron with the oxidized dye; (iii) recombination of the injected electron with the oxidized form of the redox shuttle. (c) A typical research DSC with glass/FTO/TiO_2_/dye photoanode, glass/Pt counter electrode, and electrolyte. This particular DSC contains an N-heterocyclic iron(ii) dye and an I_3_^−^/I^−^ redox mediator (photo: Dr Mariia Becker, University of Basel).

The solar spectrum (Fig. 1) peaks in the visible region, and the latter accounts for *ca.* 40% of the total radiation; 55% falls in the infrared (IR) region, and the remaining 5% in the ultraviolet (UV). When light falls on an n-type semiconductor and the photons possess energies equal to or greater than the band gap, electrons are excited from the valence to the conduction band of the semiconductor. Photoenergy conversion then follows to transform light into electrical current. Naturally, semiconductors such as silicon are optimal for such applications, but have the disadvantage that the material is not optically transparent and the majority of the photoelectric effects occur at the surface. This prompted investigations of optically transparent materials which, of course, do not absorb visible light. For electron excitation to occur, wide-band gap semiconductors such as TiO_2_ (band gap = 3.2 eV for anatase) must absorb photons with energies in the UV region. From Fig. 1, it is clear that pristine wide-band gap semiconductors are not appropriate for efficient photoenergy conversion. Although a reactive titanium-terminated anatase surface phase with a band gap of <2 eV has been discovered,^2^ the most convenient method of utilizing longer wavelength radiation is to functionalize the surface of the semiconductor with a material that absorbs in the visible region. Such materials are termed sensitizers or dyes and critically, the ground state (S) of the sensitizer must lie below the conduction band of the semiconductor, and the excited state (S*) above the conduction band (Fig. 2a).

### The dye-sensitized solar cell: a general overview

The Grätzel n-type dye-sensitized solar cell (DSC) was developed in the early 1990s, and the use of sintered nanoparticles of TiO_2_ to produce an enormous surface area while maintaining a small device is crucial to the design.^3–8^ The principle of the working device is shown schematically in Fig. 2a with detrimental recombination processes shown in Fig. 2b; Fig. 2c shows a typical laboratory device. The conducting glass must be transparent and is typically colourless glass coated with fluorine-doped tin oxide (FTO). The processes at the photoanode (Fig. 2a) are the sequential photoexcitation of the dye, electron injection into the semiconductor, and electron transfer from the reduced form of the redox shuttle to the oxidized form of the dye. After dye-excitation and electron injection, the dye is formally in an oxidized state. The redox mediator (also referred to as a redox shuttle or couple) is responsible for transferring electrons from the counter electrode through the cell to regenerate the ground state of the dye. A note at this point about terminology: it is important to distinguish between the redox mediator and the electrolyte – the *electrolyte* comprises the *redox mediator and additives in a solvent*.

**Scheme 1:**
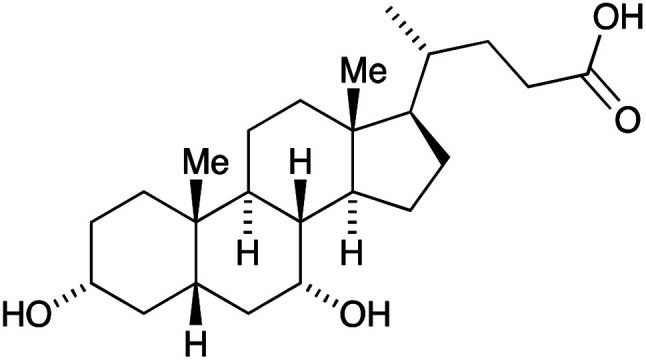
The structure of chenodeoxycholic acid (cheno).

The three essential processes mentioned above (dye photoexcitation, electron injection and dye regeneration) compete with non-beneficial electron transfers (Fig. 2b): decay of the excited state dye back to the ground state (*i.e.* no net electron injection), recombination of the injected electron with the oxidized dye (again, no net electron injection), and recombination of the injected electron with the oxidized form of the redox shuttle (once again, no electron injection). Minimizing recombination processes (back-reactions) at the interface between the semiconductor, dye and redox mediator is essential, and common ways to address this are through the use of co-adsorbents and additives. A popular additive is 4-*tert*-butylpyridine (TBP) which is added to the electrolyte in the DSC and leads to a raising of the conduction band (*E*_cond_, Fig. 2a) with a concomitant increase in the open-circuit voltage (*V*_OC_, Fig. 2a). The addition to the dye of co-adsorbents such as chenodeoxycholic acid (cheno, Scheme 1) decreases the aggregation of dye molecules, and enhances electron injection.^7,9^ Computational studies play an important role in the development of structure–property relationships for molecular sensitizers, and interactions between dye molecules and between dye and coadsorbent species.^10–12^

In an n-type DSC, the counter electrode functions as a catalyst (the Pt coating shown in Fig. 2a) for regeneration of the redox mediator and as a contact in the electrical circuit. In this type of DSC, light harvesting is governed by the dye adsorbed on the n-type semiconductor. In a double-junction or tandem DSC, both the photoanode and photocathode can be functionalized with dyes, and the solar energy conversion in the cell could, theoretically, reach *ca.* 40%.^13^ However, progress in the development of p-type DSCs is hampered by the lack of effective combinations of wide-band gap p-type semiconductors and sensitizers. The last decade has seen an explosion of interest in the use of quantum dot sensitized solar cells and perovskite solar cells. These areas are out of the scope of the present review, and readers are directed to the following articles and references therein.^14–25^

In this review, we focus on n-type DSCs and, critically, on the need to develop DSCs incorporating sustainable materials.^26^ Ideally, all components in a DSC should utilize sustainable materials. Use of TiO_2_ for the photoanode fits this criterion, with titanium having a natural abundance in the Earth's crust of *ca.* 5600 ppm.^27^ The major use of TiO_2_ is as a white pigment, and the United States Geological Survey (USGS) reported in 2021 that world resources of titanium minerals exceed two billion tons and that there is currently no recycling of TiO_2_.^28^ To date, many of the best-performing DSCs have incorporated ruthenium(ii) dyes and iodine-based redox mediators. Since ruthenium has an extremely low abundance in the Earth's crust (*ca.* 0.001 ppm),^27^ dependence on this metal for large-scale DSC production is not sustainable. Perhaps less well recognized is the low crustal abundance of iodine (*ca.* 0.14 ppm),^27^ and, as discussed later, electrolytes containing the I_3_^−^/I^−^ redox couple possess intrinsic corrosive properties.

The highest DSC photoconversion efficiency (PCE, *η*) values^29–35^ are achieved by optimizing not only the molecular design and performance of the sensitizer.^36–41^ Tuning the composition of the electrolyte is critical,^42–44^ as are optimizing both the fabrication of the photoanode, and the materials and fabrication of the counter electrode.^45^ State-of-the-art dyes for n-type DSCs are typically ruthenium(ii) coordination compounds,^46–52^ zinc(ii) porphyrinato or phthalocyanato complexes,^46,48,53–57^ and metal-free organic dyes.^47,48,58^ Natural pigments have also been thoroughly investigated, but their photoconversion efficiencies are limited.^59,60^

**Scheme 2:**
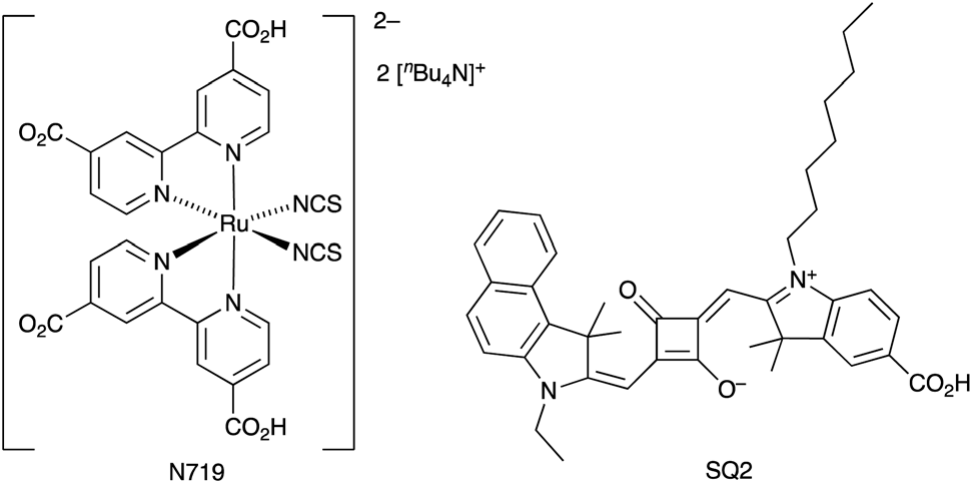
Structures of the dyes N719 and SQ2.

We end this introduction with several general comments concerning the need for consistency in reporting data. Critically, DSCs should be fully masked to prevent the overestimation of their performance.^61,62^ Wherever possible, we have only made direct comparisons between DSCs fabricated under the same or similar conditions. A second problem in overviewing the DSC literature is knowing the reproducibility of cell performances. Not all researchers report data for multiple devices. This appertains, not only to *J*_SC_ and *V*_OC_ values, but also to electrochemical impedance spectroscopic (EIS) data. We recently explored the reproducibility of EIS data for DSCs sensitized with N719 and SQ2 (Scheme 2). Whereas data for DSCs with N719 were reproducible, SQ2 proved to be an instructive example of a dye for which the EIS parameters can be rather variable within one set of DSCs with identical components and fabricated by the same person and in the same fashion.^63^

A general note about light intensity is necessary. For most routine evaluations of the performances of DSCs, devices are illuminated under a light intensity of 1 sun = 1000 W m^−2^ = 100 mW cm^−2^.††In the literature, authors report light intensity in units of either W m^−2^ or mW cm^−2^ (1 sun). For consistency, we use W m^−2^ throughout the review, and note that 1000 W m^−2^ = 100 mW cm^−2^. However, photoconversion efficiencies increase when lower light intensities are used. Such low or diffuse light sources are relevant to indoor applications of DSCs, and it is important to note that DSCs are reliable devices even under diffuse lighting conditions.^64,65^

A final comment to the introduction is that, although this review strives to cover the literature as broadly as possible, it is not fully comprehensive. We have chosen not to include studies in which the metal complexes used as sensitizers were not adequately characterized, or in which insufficient information was provided about cell fabrication.

## An unfriendly chemical environment within the DSC: enter the first row metals

### The I_3_^−^/I^−^ redox mediator works: why change it?

For efficient photoconversion efficiency, a critical factor for a redox mediator is that it can regenerate the ground state of the dye on a faster timescale than recombination events which negate electron injection. The I_3_^−^/I^−^ couple fulfils this requirement and has become an established component of most DSCs.^66^ A disadvantage, however, is that using an I_3_^−^/I^−^ redox mediator limits values of the open-circuit voltage, *V*_OC_ (Fig. 2) to 700–800 mV.^29,67^ Furthermore, its use creates a corrosive chemical environment within a dye-sensitized solar cell, limiting DSC stability.^66,68^ Thus, the past decade has seen the development of alternative and less corrosive redox mediators having more positive reduction potentials than I_3_^−^ in order to increase values of *V*_OC_.^69–72^ The most notable are those based on Co^3+^/Co^2+^ and Cu^2+^/Cu^+^ couples. In this review, we focus mainly on the use of first row d-block metal-ion redox mediators with dyes containing first row d-block metals, and the discussion in this section on the use of Co^3+^/Co^2+^ and Cu^2+^/Cu^+^ redox mediators with other dyes is limited to introductory comments and selected highlights, as well as reviews to lead the reader into the relevant literature.

### Co^3+^/Co^2+^ redox mediators: organic and zinc(ii) porphyrin dyes

**Scheme 3:**
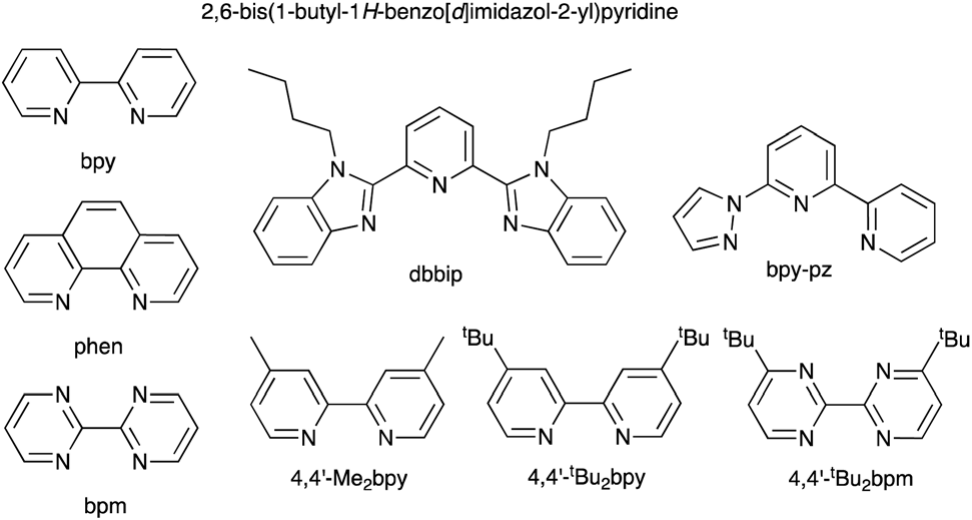
Structures of bpy, phen, bpm, dbbip, bpy-pz, 4,4′-Me_2_bpy, 4,4′-^*t*^Bu_2_bpy and 4,4′-di-*tert*-butyl-2,2′-bipyrimidine (4,4′-^*t*^Bu_2_bpm).

**Fig. 3:**
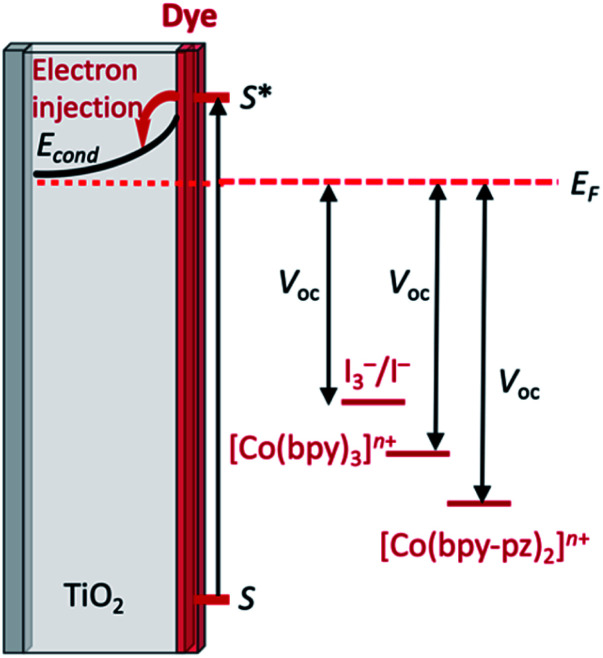
Schematic illustration of the relative *E*_redox_ levels (in red) for two representative Co^3+^/Co^2+^ redox mediators with respect to the I_3_^−^/I^−^ couple and the effect on the value of *V*_OC_ (see Fig. 2a for complete DSC diagram).

The early history of the development of Co^3+^/Co^2+^ redox mediators incorporating [Co(bpy)_3_]^3+^/[Co(bpy)_3_]^2+^ and [Co(phen)_3_]^3+^/[Co(phen)_3_]^2+^ and their derivatives (bpy = 2,2′-bipyridine, phen = 1,10-phenanthroline) and [Co(dbbip)_2_]^3+^/[Co(dbbip)_2_]^2+^ (dbbip = 2,6-bis(1-butyl-1*H*-benzo[*d*]imidazol-2-yl)pyridine, Scheme 3) was documented in 2012 by Hamann,^73^ and the electrochemical properties of Co^3+^/Co^2+^ couples and their applications in n-type DSCs containing zinc(ii) porphyrin and organic dyes have been thoroughly reviewed.^72,74–79^ One of the beauties of the Co^3+^/Co^2+^ redox couple is that the standard reduction potential, *E*° (and hence *E*_redox_, Fig. 2a) can be easily tuned by varying the coordinated ligands, and this is a way of increasing the value of *V*_OC_ (Fig. 3). Berlinguette and coworkers demonstrated a linear relationship between *E*°(Co^3+^/Co^2+^) and *V*_OC_ for a series of [CoL_3_]^3+^/[CoL_3_]^2+^ redox couples containing bpy, 2,2′-bipyrimidine (bpm), 4,4′-di-*tert*-butyl-2,2′-bipyridine or 4,4′-di-*tert*-butyl-2,2′-bipyrimidine (Scheme 3) ligands.^80^ The use of polydentate ligands enhances the stability of the cobalt(ii) complexes with respect to ligand dissociation.^81,82^

**Scheme 4:**
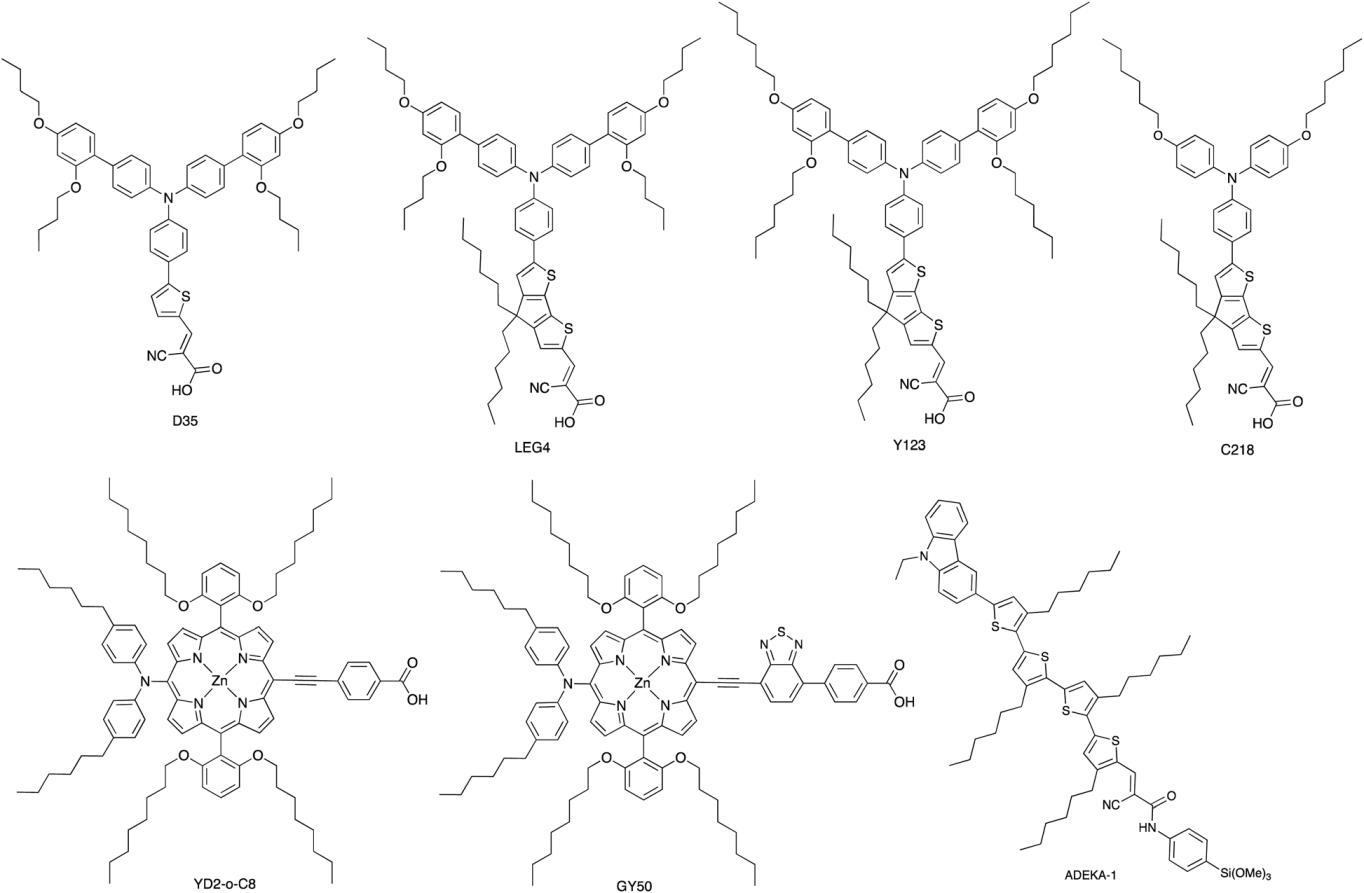
Structures of some of the high extinction coefficient metal-free and zinc(ii) porphyrin dyes used with cobalt(ii)/(iii) and/or copper(ii)/(i) redox mediators.

The use of the Co^3+^/Co^2+^ redox shuttle was initially demonstrated by Nusbaumer *et al.* who showed that the redox potential of [Co(dbbip)_2_]^3+^/[Co(dbbip)_2_]^2+^ in MeCN was comparable to that of I_3_^−^/I^−^, and that the kinetics of electron transfer of the two redox shuttles in a DSC were similar. Moreover, the weak visible light absorption by both [Co(dbbip)_2_]^3+^ and [Co(dbbip)_2_]^2+^ leads to minimal competition with light absorption with the dye in a DSC.^83^ This is also true of other cobalt(ii)/(iii) coordination compounds used as redox mediators. Potentials for the [Co(bpy)_3_]^3+^/[Co(bpy)_3_]^2+^ and [Co(phen)_3_]^3+^/[Co(phen)_3_]^2+^ couples are +0.56 and +0.62 V, respectively (*vs.* NHE);^84^ these are significantly more positive than *E*° for I_3_^−^/I^−^ (+0.31 V *vs.* NHE). In 2010, Feldt *et al.* achieved *V*_OC_ and *J*_SC_ values of 920 mV and 10.7 mA cm^−2^, respectively, for DSCs containing a combination of [Co(bpy)_3_]^3+^/[Co(bpy)_3_]^2+^ with the donor–π-bridge–acceptor (D–π–A) triphenylamine dye D35 (Scheme 4) under a light intensity of 1000 W m^−2^. Mass transport limitations associated with the sterically demanding cationic cobalt complexes were circumvented by careful matching of energy levels of dye and the [Co(bpy)_3_]^3+^/[Co(bpy)_3_]^2+^ couple. With D35, the overall photoconversion efficiency was 6.7%, and the inclusion of the butoxy chains in dye D35 reduced recombination.^85^ Note that in [Co(dbbip)_2_]^3+^/[Co(dbbip)_2_]^2+^, long alkyl chains were introduced into the cobalt complex, but it proves beneficial to incorporate them into the dye structure rather than the cobalt redox mediator. On the other hand, Mozer and coworkers have shown that the electron lifetime increases considerably when both the dye and the Co^3+^/Co^2+^ shuttle contain alkyl chains.^86^

A dramatic improvement in PCE to 11.9% was achieved by combining the [Co(bpy)_3_]^3+^/[Co(bpy)_3_]^2+^ shuttle with the donor–π-bridge–acceptor zinc(ii) porphyrin dye YD2-*o*-C8 (Scheme 4), and co-sensitization with Y123 (Scheme 4) led to a further enhancement to 12.3%. With the related dye GY50 (Scheme 4), a record PCE of 12.75% was attained. Key to these successes are the high values of *V*_OC_ (965 mV for a DSC with YD2-*o*-C8, and 885 mV with GY50),^29,67,87^ and Fig. 3 illustrates the effect on *E*_redox_ (defined in Fig. 2a and 3) upon going from I_3_^−^/I^−^ to [Co(bpy)_3_]^3+^/[Co(bpy)_3_]^2+^. A further lowering of the potential is achieved upon going from [Co(bpy)_3_]^3+^/[Co(bpy)_3_]^2+^ to [Co(bpy-pz)_2_]^3+^/[Co(bpy-pz)_2_]^2+^ (*E*° = +0.86 V *vs.* NHE, see Scheme 3 for bpy-pz), and DSCs sensitized with dye Y123 coupled with [Co(bpy-pz)_2_]^3+^/[Co(bpy-pz)_2_]^2+^ achieved *V*_OC_, *J*_SC_ and PCE values of 1020 mV, 12.54 mA cm^−2^ and 8.87% under an illumination of 1000 W m^−2^. This compared with *V*_OC_ = 754 mV, *J*_SC_ = 13.01 mA cm^−2^ and *η* = 6.57% for an analogous DSC containing I_3_^−^/I^−^. The DSC performances are also dependent upon the thickness of the TiO_2_ layer.^88^ The addition of electron-donating TPAA (TPAA = tris(4-methoxyphenyl)amine) to the electrolyte is beneficial. TPAA (like (2,2,6,6-tetramethylpiperidin-1-yl)oxyl, TEMPO^89^) acts as an intermediate redox species, increasing the rate of dye regeneration. Electrons are transferred from TPAA to the oxidized dye in an extremely fast process (100–1000 ps), and are then transferred from [Co(bpy)_3_]^2+^ to the oxidized form of TPAA (TPAA˙^+^). DSCs with the organic dye LEG4 (Scheme 4) and containing [Co(bpy)_3_]^3+^/[Co(bpy)_3_]^2+^ as redox shuttle, with and without TPAA in the electrolyte, attained values of *V*_OC_, *J*_SC_ and *η* values of 915 *vs.* 835 mV, 14.1 *vs.* 12.1 mA cm^−2^ and 9.1 *vs.* 7.2%, respectively, under an illumination of 1000 W m^−2^.^90^ In 2014, Kakiage *et al.* reached PCEs of up to 12.5% in DSCs sensitized with the dye ADEKA-1 (Scheme 4) combined with the [Co(5-Clphen)_3_]^3+^/[Co(5-Clphen)_3_]^2+^ redox mediator (5-Clphen = 5-chloro-1,10-phenanthroline, *E*° = +0.72 V *vs.* NHE). For a set of three cells, average values of *V*_OC_, *J*_SC_ and PCE values were 1036 mV, 15.6 mA cm^−2^ and 12.5% under an illumination of 1000 W m^−2^.^32^ This PCE was enhanced further by co-sensitization of ADEKA-1 with LEG4 (Scheme 4), and for four DSCs, the average *V*_OC_, *J*_SC_ and PCE values were 1014 mV, 18.27 mA cm^−2^ and 14.3% (light intensity 1000 W m^−2^) and using a [Co(phen)_3_]^3+^/[Co(phen)_3_]^2+^ redox shuttle.^34^ Yella *et al.* demonstrated the effects of pore size and porosity of the TiO_2_ layer and the viscosity of the electrolyte on DSC performance. They concluded that porosity and pore size must be modified for different combinations of dyes and electrolytes in order to minimize the diffusion limitations of the cobalt-based redox mediator,^91^ and these conclusions are consistent with those of Boschloo and coworkers.^92^

### Co^3+^/Co^2+^ redox mediators: ruthenium(ii) dyes

**Scheme 5:**
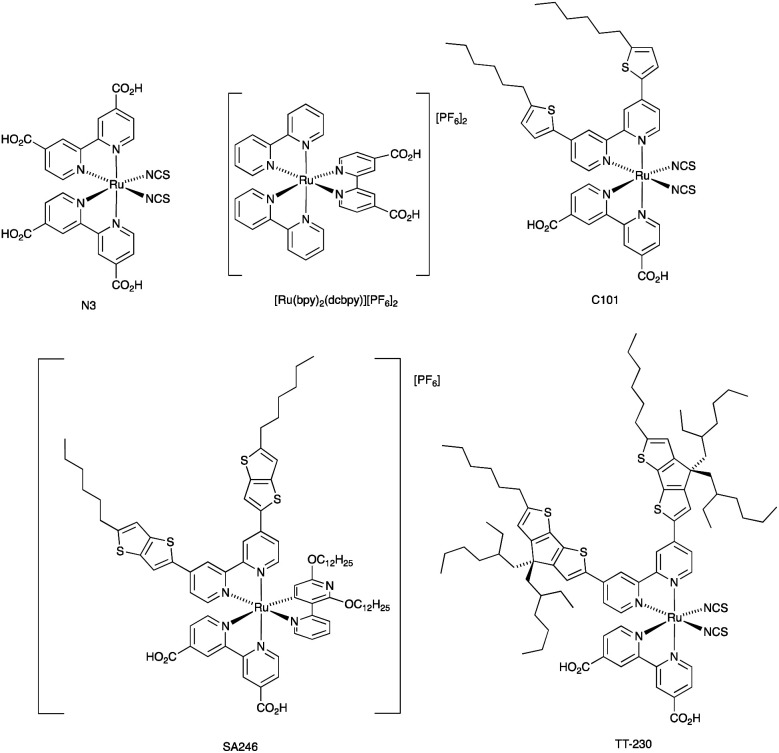
Structures of the ruthenium(ii) dyes discussed in the text; see Scheme 2 for the structure of N719.

The I_3_^−^/I^−^ redox shuttle was originally optimized for compatibility with ruthenium(ii) dyes such as N719 and N3 (Schemes 2 and 5). In contrast to the metal-free and zinc(ii) porphyrin dyes described above, a simple move from I_3_^−^/I^−^ to cobalt-based redox mediators to increase *V*_OC_ was not achieved with these conventional Ru(ii) dyes due to dominant recombination processes. Intermolecular interactions between the ruthenium(ii) sensitizer and cobalt(iii)/(ii) species must be limited, and the use of a coadsorbent such as cheno,^93^ and/or a shift in design of the ruthenium dye were required.^94–96^ We provide selected examples here, and otherwise direct the reader to reviews that are focused on this topic.^71,76,77^ Dye regeneration by redox mediators such as [Co(4,4′-Me_2_bpy)_3_]^3+^/[Co(4,4′-Me_2_bpy)_3_]^2+^ and [Co(4,4′-^*t*^Bu_2_bpy)_3_]^3+^/[Co(4,4′-^*t*^Bu_2_bpy)_3_]^2+^ (see Scheme 3 for bpy derivatives) is efficient for [Ru(bpy)_2_(dcbpy)][PF_6_]_2_ (Scheme 5).^97^ Dye C101 (Scheme 5) possesses long chains to militate against recombination but is still representative of a ‘conventional’ ruthenium(ii) dye.^98^ Under irradiation of 1000 W m^−2^, a DSC with C101 combined with a [Co(bpy)_3_]^3+^/[Co(bpy)_3_]^2+^ redox shuttle gave *V*_OC_ = 735 mV and *J*_SC_ = 6.5 mA cm^−2^ and overall *η* = 3.6%. Upon going to the more sterically demanding dye TT-230 (Scheme 5), a higher *V*_OC_ was attained (774 mV) which could be boosted to 804 mV with the addition of the coadsorbent cheno. However, this was at the expense of *J*_SC_ (3.3 and 3.0 mA cm^−2^, without and with cheno).^98^ Thiocyanate-free ruthenium(ii) dyes are a promising route forward for reducing recombination and enhancing compatibility with cobalt-based redox mediators,^99,100^ and cyclometallated ruthenium(ii) sensitizers have also been investigated.^101,102^ PCE values of between 6.1 and 9.4% were obtained for DSCs sensitized with a series of cyclometallated Ru(ii) dyes using a [Co(phen)_3_]^3+^/[Co(phen)_3_]^2+^ redox shuttle; the best performing dye (*V*_OC_ = 845 mV, *J*_SC_ = 14.55 mA cm^−2^ and *η* = 9.4% under a light intensity of 1000 W m^−2^) is SA246 shown in Scheme 5.^102^

### Cu^2+^/Cu^+^ redox mediators: breaking the 1000 mV *V*_OC_ barrier

**Scheme 6:**
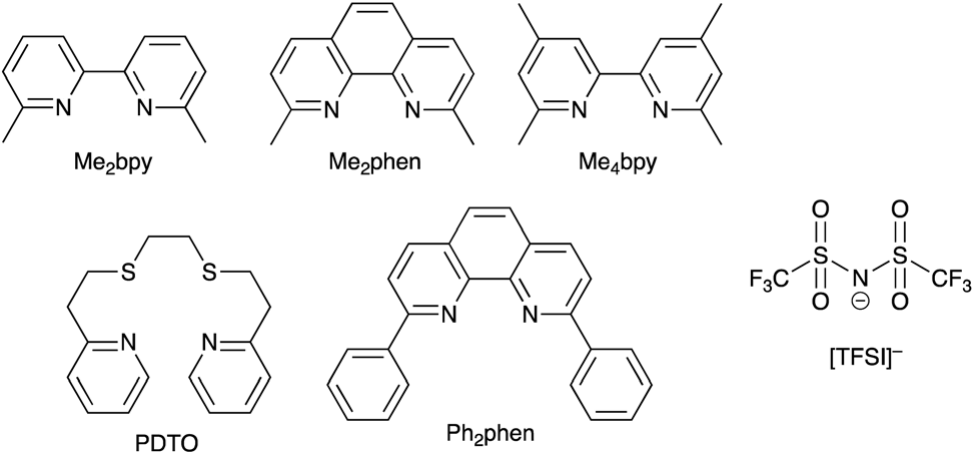
Structures of some of N^N ligands used in copper(ii)/copper(i) redox mediators, and the structure of the [TFSI]^−^ anion.

Copper-based redox mediators^70,103–105^ entered the arena in 2005,^106^ but little further progress was made until a report from Bai *et al.* in 2011.^107^ This was followed by highly promising results from Li *et al.*,^108^ Magni *et al.*,^109^ Freitag *et al.*^110^ and Saygili *et al.*^111^ Homoleptic bis(diimine)copper(i) complexes such as [Cu(bpy)_2_]^+^ and [Cu(phen)_2_]^+^ are tetrahedral, while the corresponding copper(ii) compounds are tetragonal. Flattening of the coordination sphere upon oxidation means that the Cu^+^ to Cu^2+^ potential is shifted to more positive values when substituents are introduced into the 6,6′-positions of bpy or the 2,9-positions of phen. For example, *E*° values (*vs.* NHE) are +0.87 V for [Cu(Me_4_bpy)_2_]^2+^/[Cu(Me_4_bpy)_2_]^+^, +0.97 V for [Cu(Me_2_bpy)_2_]^2+^/[Cu(Me_2_bpy)_2_]^+^, and +0.93 V for [Cu(Me_2_phen)_2_]^2+^/[Cu(Me_2_phen)_2_]^+^.^111^ Ligand abbreviations are defined in Scheme 6. In order to provide good solubilities in typical electrolyte solvents, the copper complexes are usually used as the [TFSI]^−^ salts (Scheme 6).

Bai *et al.* showed that DSCs sensitized with the organic dye C218 (Scheme 4) and using a [Cu(Me_2_phen)_2_]^2+^/[Cu(Me_2_phen)_2_]^+^ redox couple could attain an *η* value of 7.0% under irradiation of 1000 W m^−2^, with values of *J*_SC_ = 11.29 mA cm^−2^ and *V*_OC_ = 932 mV. Analogous DSCs with an I_3_^−^/I^−^ redox mediator achieved higher *J*_SC_ (13.74 mA cm^−2^) but significantly lower *V*_OC_ (714 mV), leading to a lower overall *η* value of 6.5%.^107^ These results were pivotal in delineating the use of copper(ii)/(i) redox mediators, but at the same time, Bai *et al.* also commented upon the very low electron-transfer rates at the counter-electrode interface and the need for careful choice of counter-electrode materials compatible with copper(ii)/(i) redox couples.^107^ The dyes C218 and LEG4 have related structures (Scheme 4), and Freitag *et al.* demonstrated that masked DSCs containing LEG4 and the [Cu(Me_2_phen)_2_]^2+^/[Cu(Me_2_phen)_2_]^+^ redox shuttle realized open-circuit voltages in excess of 1000 mV under irradiation of 1000 W m^−2^. The best performing DSC exhibited values of *V*_OC_ = 1020 mV, *J*_SC_ = 12.6 mA cm^−2^ and *η* = 8.3%, with the high value of *V*_OC_ exceeding the 875 mV recorded for a cell containing LEG4 and the cobalt-based redox couple [Co(bpy)_3_]^3+^/[Co(bpy)_3_]^2+^. The copper-based redox mediator was found to exhibit both higher diffusion coefficients and faster dye regeneration than [Co(bpy)_3_]^3+^/[Co(bpy)_3_]^2+^. However, this study revealed a number of recombination pathways involving the [Cu(Me_2_phen)_2_]^2+^/[Cu(Me_2_phen)_2_]^+^ redox mediator, including the reductive quenching of the excited-state dye and interaction with the FTO/TiO_2_ layer.^110^ The low driving force (0.2 eV) for regeneration of the dye^110^ could be decreased further by using a [Cu(Me_2_bpy)_2_]^2+^/[Cu(Me_2_bpy)_2_]^+^ redox shuttle as was shown by Saygili *et al.*^111^ and by Li *et al.*^108^ in DSCs with the dye Y123 (Scheme 4). Only a small structural perturbation occurs upon going from [Cu(Me_2_bpy)_2_]^2+^ to [Cu(Me_2_bpy)_2_]^+^, and *vice versa*, and this contributes to rapid electron self-exchange. Table 1 presents data from the independent work of Li *et al.* and Saygili *et al.* demonstrating the influence of varying the redox shuttle from I_3_^−^/I^−^ to [Co(bpy)_3_]^3+^/[Co(bpy)_3_]^2+^ to [Cu(Me_2_bpy)_2_]^2+^/[Cu(Me_2_bpy)_2_]^+^. The rise in *V*_OC_ is the essential parameter that leads to enhanced photoconversion efficiency. Of particular importance is the observation that the photovoltage remains above 1000 mV down to 0.2 sun light intensity, making the use of copper-based redox mediators appealing for indoor applications.^111^ These landmark results are not confined to liquid DSCs. In 2015, Frietag *et al.* demonstrated 8.2% efficiency in solid-state DSCs under a light intensity of 1000 W m^−2^ using a solid hole transport material comprising a mixture of [Cu(Me_2_phen)_2_][TFSI]_2_ and [Cu(Me_2_phen)_2_][TFSI] with TiO_2_ sensitized with the dye LEG4. The notable value of *η* = 8.2% was a consequence of high values of *V*_OC_ = 1010 mV and *J*_SC_ = 13.8 mA cm^−2^.^112^

**Table 1:** The effects of the redox mediator on DSC performances using dye Y123 (under a light intensity of 1000 W m^−2^) from the work of Li *et al.*^108^ (entries 1–3 in the table) and Saygili *et al.*^111^ (entries 4–6)

Entry	Redox couple	*J*_SC_/mA cm^−2^	*V*_OC_/mV	ff^a^/%	*η*/%
1	I_3_^−^/I^−^	15.8 ± 0.3	724 ± 10	70.4 ± 0.6	8.0 ± 0.2
2	[Co(bpy)_3_]^3+^/[Co(bpy)_3_]^2+^	15.3 ± 0.3	844 ± 5	71.2 ± 0.7	9.2 ± 0.1
3	[Cu(Me_2_bpy)_2_]^2+^/[Cu(Me_2_bpy)_2_]^+^	14.4 ± 0.2	1048 ± 7	68.1 ± 0.5	10.3 ± 0.1
4	[Cu(Me_2_bpy)_2_]^2+^/[Cu(Me_2_bpy)_2_]^+^	14.15	1070	68.7	10.0
5	[Cu(Me_4_bpy)_2_]^2+^/[Cu(Me_4_bpy)_2_]^+^	15.53	1040	64.0	10.3
6	[Cu(Me_2_phen)_2_]^2+^/[Cu(Me_2_phen)_2_]^+^	13.61	1060	69.2	10.3

a ff = fill factor.

### Cu^2+^/Cu^+^ redox mediators: interactions with Lewis base additives in the electrolyte

Earlier, we noted the use of TBP as a common additive to the electrolyte in DSCs containing the I_3_^−^/I^−^ redox couple because of the associated increase in values of *V*_OC_. The use of TBP is not confined to use with I_3_^−^/I^−^. However, its role in electrolytes using Cu^2+^/Cu^+^ couples poses particular problems because of the possibility for coordination of TBP to copper and the associated consequences on the electrochemical behaviour of the redox mediator. The results of investigations of the interactions of TBP and other Lewis bases with [Cu(N^N)_2_]^2+^ species have resulted in a rather complicated picture, although the underlying message is that TBP is a coordinatively non-innocent additive to DSC electrolytes.

**Fig. 4:**
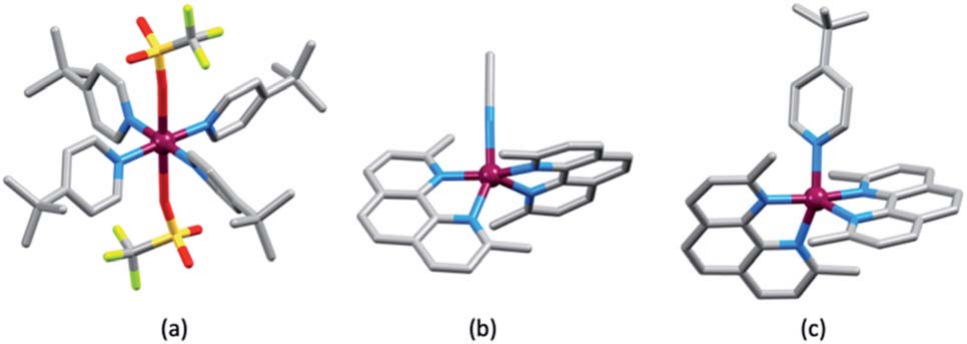
The structures of (a) *trans*-[Cu(TBP)_4_(O_3_SCF_3_)_2_] (CSD^120^ refcode IPEWAD), (b) the [Cu(Me_2_phen)_2_(NCMe)]^2+^ cation in the perchlorate salt (CSD refcode XIDWEP), and (c) the [Cu(Me_2_phen)_2_(TBP)]^2+^ cation from the structure of the TFSI^−^ salt; the cif was kindly provided by the authors of ref. 119. Hydrogen atoms are omitted for clarity. As a general note, 3D-structures in this review have been drawn using coordinates retrieved from the Cambridge Structural Database (CSD, version 2021.2.0)^121^ and using Mercury version 2021.2.0.^122^

In an investigation of the effects of the Lewis bases TBP, 2,6-bis(*tert*-butyl)pyridine, 4-methoxypyridine and 4-(5-nonyl)pyridine, Hagfeldt and coworkers concluded that the optimization of the pyridine base used in DSC electrolytes containing a copper-based redox shuttle depended upon a balance of basicity and coordination capacity.^113^ In 2016, Saygili *et al.* pointed to possible changes in the copper(ii) coordination sphere, especially for [Cu(Me_2_bpy)_2_]^2+^ and [Cu(Me_4_bpy)_2_]^2+^, that could be caused by both TBP and [TFSI]^−^.^111^ Hupp and coworkers found that when a [Cu(PDTO)]^2+^/[Cu(PDTO)]^+^ redox couple (PDTO, see Scheme 6) was used in the presence of TBP in a DSC electrolyte, TBP ligands displaced the tetradentate PDTO in the oxidized form of the redox mediator. They were able to isolate single crystals of *trans*-[Cu(TBP)_4_(CF_3_SO_3_)_2_] (Fig. 4) from a CH_2_Cl_2_ solution of [Cu(PDTO)][CF_3_SO_3_]_2_ containing a 10-fold excess of TBP. Hupp proposed that in a MeCN-based electrolyte, likely copper(ii) species would be 4-, 5- or 6-coordinate [Cu(TBP)_4+*x*_(NCMe)_*y*_]^2+^ ions with the fifth or sixth coordination site occupied by MeCN or TBP ligands.^114^ In 2018, Wang and Hamman proposed that TBP could displace ligands such as Me_2_bpy (Scheme 6) to give [Cu(TBP)_4_]^2+^ in MeCN solution. In the electrolyte in a DSC which initially contains [Cu(Me_2_bpy)_2_]^2+^/[Cu(Me_2_bpy)_2_]^+^ redox mediator, it was proposed that when TBP is in sufficient excess, [Cu(TBP)_4_]^2+^/[Cu(Me_2_bpy)_2_]^+^ is the pertinent redox species. Since [Cu(TBP)_4_]^2+^ is a poor electron acceptor, recombination is reduced which contributes to enhanced *J*_SC_ and *V*_OC_.^115^ In contrast, Saygili *et al.* favoured a 5-coordinate complex (based upon density functional theory calculations) in which one molecule of TBP adds to the [Cu(N^N)_2_]^2+^ species. This results in different charge recombination kinetics, but nonetheless, the recombination resistance and electron lifetime values were higher for copper-based than for cobalt-based redox mediators.^116^

Yeh, Wei and coworkers focused on the interactions of MeCN (a common electrolyte solvent) and TBP with components of the [Cu(Me_2_phen)_2_]^2+^/[Cu(Me_2_phen)_2_]^+^ redox mediator.^117^ Firstly, they demonstrated the formation of the 5-coordinate complex [Cu(Me_2_phen)_2_(NCMe)]^2+^ in MeCN solutions containing [Cu(Me_2_phen)_2_]^2+^, and this is consistent with the report of Kloo and coworkers that crystals of [Cu(Me_2_phen)_2_(NCMe)][ClO_4_]_2_ (Fig. 4b) grow from an MeCN solution of [Cu(Me_2_phen)_2_][ClO_4_]_2_.^118^ Yeh, Wei and coworkers further showed that the addition of 15 equivalents of TBP (*i.e.* replicating the [Cu(Me_2_phen)_2_]^2+^ : TBP ratio in a typical DSC electrolyte) resulted in coordination of TBP to the Cu(ii) centre. From absorption spectroscopic data, they concluded that the species present was [Cu(Me_2_phen)_2_(TBP)(NCMe)_*x*_]^2+^ where *x* = 0 or 1. This investigation confirmed a negative shift in the redox potential compared to that of pristine [Cu(Me_2_phen)_2_]^2+^/[Cu(Me_2_phen)_2_]^+^. Additionally, the performance of DSCs over a 46 day period suffered from a significant decrease in the fill-factor which has its origins in reduced charge transfer at the counter electrode and slow mass transport associated with the sterically demanding [Cu(Me_2_phen)_2_(TBP)(NCMe)_*x*_]^2+^ cations.^117^

**Scheme 7:**
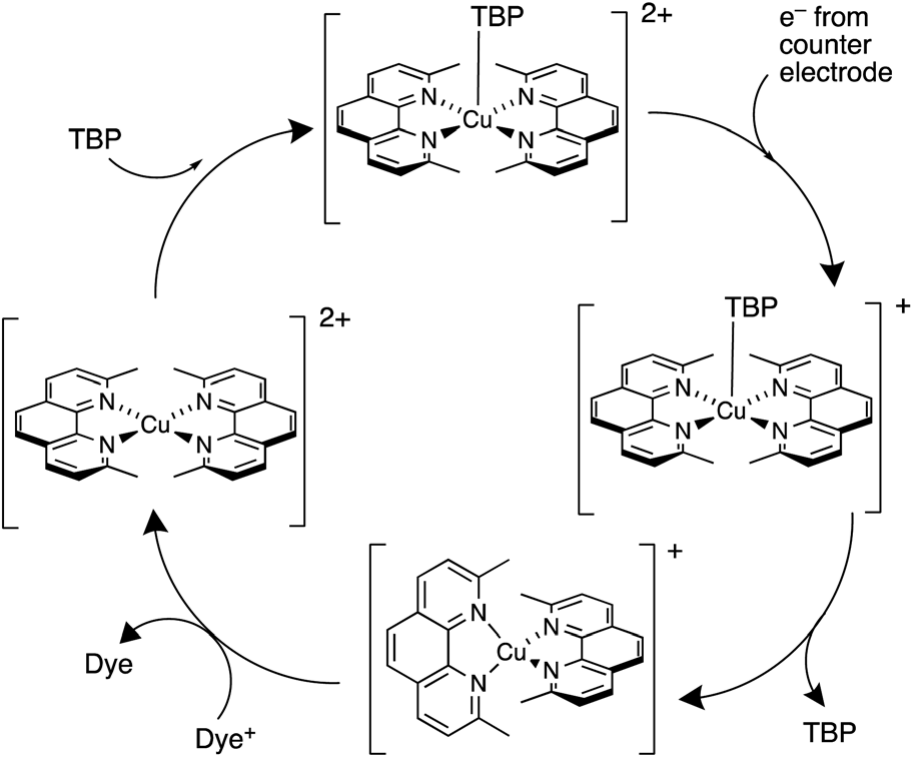
Representation of the involvement of a strong Lewis base such as TBP in the redox cycle of the [Cu(Me_2_phen)_2_]^2+^/[Cu(Me_2_phen)_2_]^+^ mediator. Based on a scheme from the work of Bach and coworkers.^119^

The pros and cons of using TBP as an additive continue to be debated. Recently, Fürer *et al.* presented a detailed investigation that confirms the critical benefits of adding strong Lewis bases such as TBP or 1-methylbenzimidazole (NMBI) to the electrolyte, but importantly, these results distinguish between the formation of 5-coordinate complexes [Cu(Me_2_phen)_2_(LB)]^2+^ (LB = Lewis base) and ligand exchange to give [Cu(LB)_4_]^2+^. The latter is exemplified with the redox mediator [Cu(Ph_2_phen)_2_]^2+^/[Cu(Ph_2_phen)_2_]^+^ (Ph_2_phen, see Scheme 6). Crystallographic data confirm the formation of [Cu(Me_2_phen)_2_(TBP)][TFSI]_2_ (Fig. 4c). Fürer *et al.* conclude that NMBI is a better additive than TBP in DSCs sensitized with Y123 (Scheme 4) and containing the [Cu(Me_2_phen)_2_]^2+^/[Cu(Me_2_phen)_2_]^+^ redox shuttle. The Lewis base additive is essential for the best-performing DSCs (*J*_SC_ > 1100 mV), but at the same time, careful choice of Lewis base to optimize coordination rather than ligand exchange at copper(ii) is critical. The formation of [Cu(LB)_4_]^2+^ has a detrimental effect on the regeneration of the reduced form of the redox mediator at the counter electrode (Scheme 7) and, therefore, limits the current output of the DSC.^119^

**Scheme 8:**
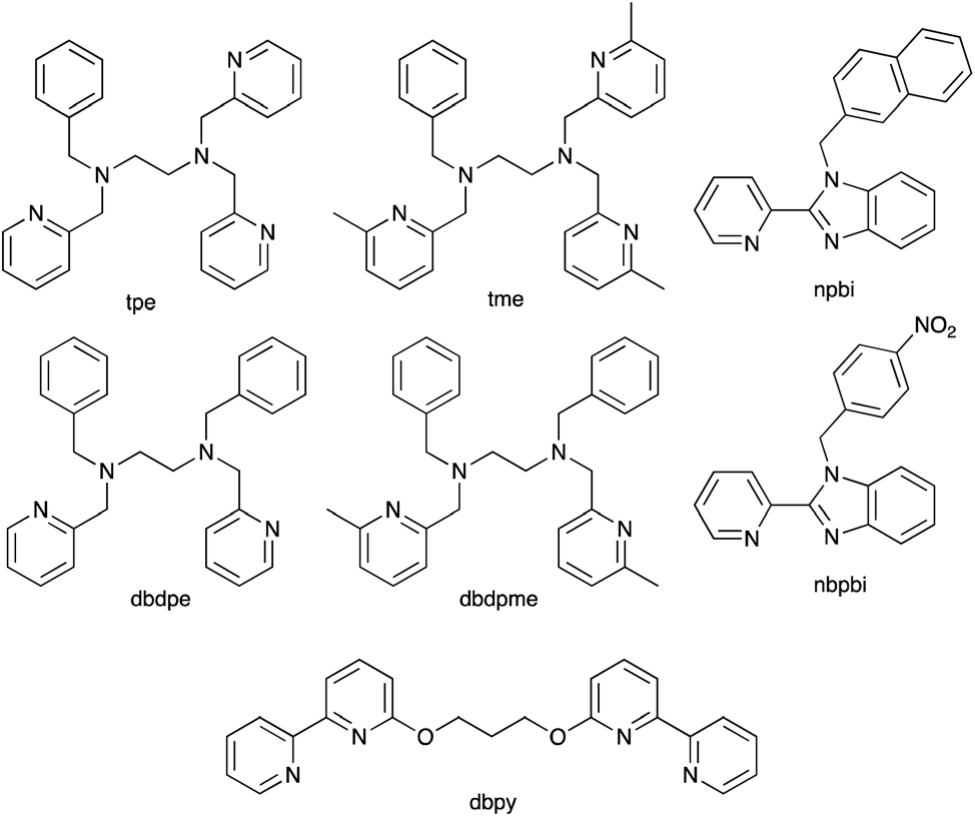
Structures of the pentatdentate ligands tpe and tme and the related tetradentate ligand dbdpe, the bidentate ligands npbi and nbpbi, and a bpy-based ligand dbpy designed to form a double stranded copper(i) helicate.

With the aim of sterically protecting the copper centre from attack by a Lewis base and, at the same time, increasing the stabilities of the Cu(i) and Cu(ii) species, Sun and coworkers designed the [Cu(tpe)]^2+^/[Cu(tpe)]^+^ and [Cu(tme)]^2+^/[Cu(tme)]^+^ redox mediators in which tpe and tme are pentadentate ligands (Scheme 8).^123^ These are closely related to the tetradentate ligands dbdpe and dbdpme (Scheme 8) which had previously proved promising in [CuL]^2+^/[CuL]^+^ (L = dbdpe or dbdpme) redox mediators when combined in DSCs with the dye Y123.^124^ Rodrigues *et al.* have also explored the use of tetradentate ligands in Cu^2+^/Cu^+^ redox shuttles where a rigid ligand backbone was found to lead to more efficient electron transfer and to enhanced *J*_SC_ values.^125^ Returning to the [Cu(tpe)]^2+^/[Cu(tpe)]^+^ and [Cu(tme)]^2+^/[Cu(tme)]^+^ couples, the presence of the methyl substituents in the ligand tme results in a higher oxidation potential for [Cu(tme)]^+^ (+0.52 V *vs.* NHE) than for [Cu(tpe)]^+^ (+0.10 V). The crystal structures of [Cu(tpe)][PF_6_]_2_ and [Cu(tme)][PF_6_]_2_ were determined, and in the latter, the Cu–N bond lengths are longer (1.990(4)–2.262(4) Å) than in the former (1.969(2)–2.113(2) Å). In DSCs sensitized with dye Y123 (Scheme 4) and containing PEDOT (PEDOT = poly(3,4-ethylenedioxythiophene)), use of the [Cu(tme)]^2+^/[Cu(tme)]^+^ redox mediator resulted in values for the best-performing cells of *J*_SC_ = 15.9 mA cm^−2^, *V*_OC_ = 840 mV and *η* = 9.4% when the device was irradiated under a light intensity of 1000 W m^−2^. Corresponding values for cells with [Cu(tpe)]^2+^/[Cu(tpe)]^+^ were *J*_SC_ = 6.6 mA cm^−2^, *V*_OC_ = 510 mV and *η* = 2.1%, with the much lower *V*_OC_ being consistent with the difference in redox potentials (see above). The stability of the system is evidenced by the fact that DSCs containing [Cu(tme)]^2+^/[Cu(tme)]^+^ maintained >90% of their initial PCE after 400 hours of continuous illumination.^123^

An alternative approach to overcome the detrimental effects of TBP is to develop TBP-free electrolytes. One interesting direction has been to design double-stranded helical dicopper complexes with a redox process based upon equilibria (1)–(4); the relative importance of each equilibrium depends upon the ligand, L. With L = dbpy (Scheme 8), the highest PCE achieved for DSCs sensitized with Y123 (Scheme 4) was *ca.* 8%, suggesting that the use of these dinuclear species is a promising way forward.^126^

1 [Cu^I^L]^+^ ⇌ [Cu^II^L]^2+^ + e^−^

2 [Cu^I^_2_L_2_]^2+^ ⇌ [Cu^I^Cu^II^L_2_]^3+^ + e^−^

3 [Cu^I^Cu^II^L_2_]^3+^ ⇌ [Cu^II^_2_L_2_]^4+^ + e^−^

4 [Cu^I^_2_L_2_]^2+^ ⇌ [Cu^II^_2_L_2_]^4+^ + 2e^−^

### Cu^2+^/Cu^+^ redox mediators: from metal-free to ruthenium(ii) dyes

**Scheme 9:**
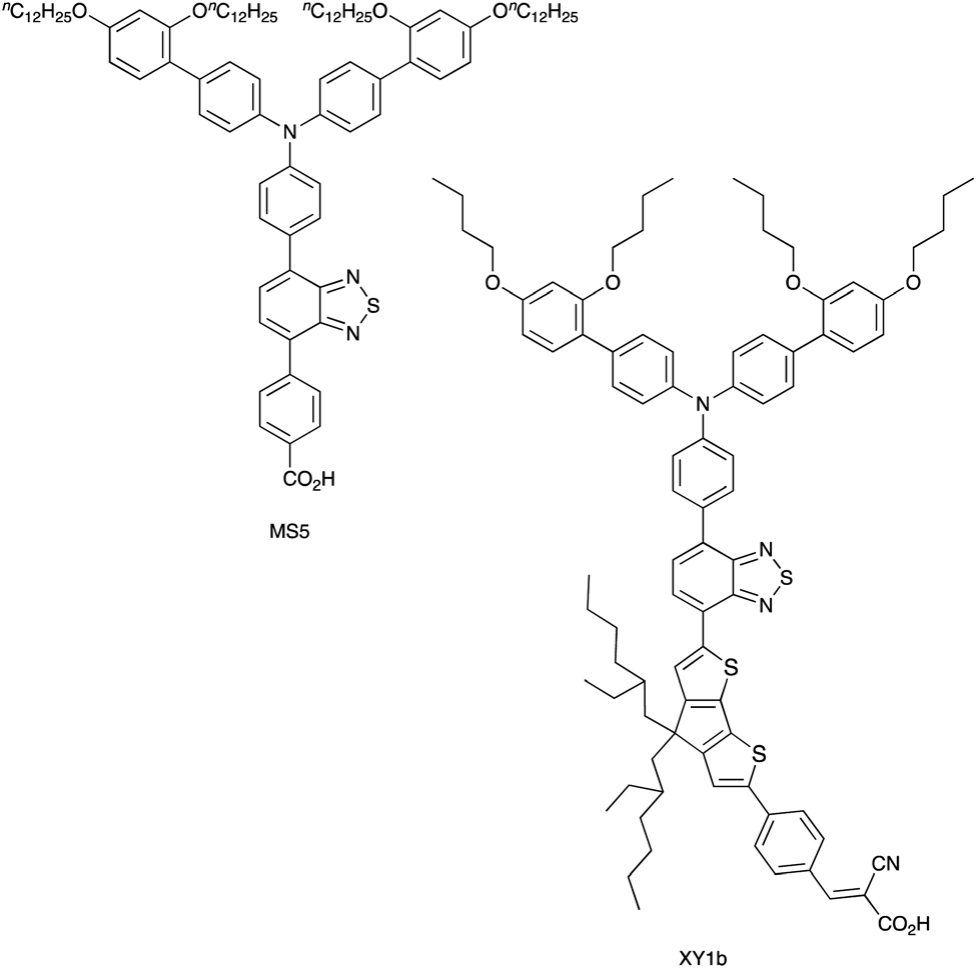
Structures of the metal-free dyes MS5 and XY1b.

Over the 2020–2021 period, the number of publications focusing on DSCs which combine organic or zinc(ii) porphyrin dyes with Cu^2+^/Cu^+^ redox shuttles has increased significantly,^127–137^ with some record breaking DSC performances originating in extremely high *V*_OC_ values. Colombo *et al.* recently reviewed the field.^104^ A particularly striking example is the realization of a *V*_OC_ value of 1240 mV for a DSC containing the dye MS5 (Scheme 9) and the [Cu(Me_4_bpy)_2_]^2+^/[Cu(Me_4_bpy)_2_]^+^ redox shuttle. The absorption maximum of MS5 in the visible range lies between *ca.* 420 and 540 nm, and in order to extend this towards to red, DSCs co-sensitized with MS5 and XY1b (Scheme 9) were investigated. Table 2 summarizes data for DSCs with MS5, XY1b and a combination of the two dyes, all with the [Cu(Me_4_bpy)_2_]^2+^/[Cu(Me_4_bpy)_2_]^+^ redox shuttle. Under ambient lighting, the co-sensitized DSC reached a record-breaking 34.5% photoconversion efficiency.^137^

**Table 2:** DSC (masked) performances using MS5, XY1b and a combination of the two dyes (under a light intensity of 1000 W m^−2^) with the [Cu(Me_4_bpy)_2_]^2+^/[Cu(Me_4_bpy)_2_]^+^ redox shuttle^137^

Dye	*J*_SC_/mA cm^−2^	*V*_OC_/mV	ff/%	*η*/%
MS5	8.87 ± 0.21	1240 ± 3	73.3 ± 0.4	8.0 ± 0.3
XY1b	15.26 ± 0.18	1010 ± 3	76.3 ± 0.2	11.8 ± 0.2
Co-sensitized	15.84 ± 0.24	1050 ± 2	81.3 ± 0.2	13.5 ± 0.2

Compatibility between ruthenium(ii) dyes and Cu^2+^/Cu^+^ redox couples in order to achieve high photoconversion efficiencies remains a challenge. Shanmugan and coworkers recently reported the performances of DSCs containing N719 or N3 (Schemes 2 and 5) and [Cu(nbpbi)_2_]^2+^/[Cu(nbpbi)_2_]^+^ (*E*° = +0.68 V *vs.* NHE) or [Cu(npbi)_2_]^2+^/[Cu(npbi)_2_]^+^ (*E*° = +0.61 V *vs.* NHE) in MeCN with TBP as an additive (see Scheme 8 for the ligand structures). Analogous DSCs containing [Co(nbpbi)_3_]^3+^/[Cu(nbpbi)_3_]^2+^ or [Co(npbi)_3_]^3+^/[Cu(npbi)_3_]^2+^ were also fabricated. In keeping with the more positive redox potentials of the copper-containing redox mediators, DSCs with the latter out-performed those with the cobalt-based couples. Table 3 displays DSC parameters for cells with [Cu(nbpbi)_2_]^2+^/[Cu(nbpbi)_2_]^+^ and [Cu(npbi)_2_]^2+^/[Cu(npbi)_2_]^+^, and these DSCs represent state-of-the-art combinations of ruthenium(ii) dyes and copper-based redox mediators. Factors contributing to the performances include relatively long electron lifetimes, slow recombination processes and rapid dye regeneration. However, the relatively low ff values in Table 3 are noteworthy.^138^

**Table 3:** DSC (masked) performances using dyes N719 and N3 (under a light intensity of 1000 W m^−2^) from the work of Shanmugan and coworkers.^138^ The electrolytes contained TBP

Redox couple	Dye	*J*_SC_/mA cm^−2^	*V*_OC_/mV	ff/%	*η*/%
[Cu(nbpbi)_2_]^2+^/[Cu(nbpbi)_2_]^+^	N719	14.3	760	44	4.82
[Cu(nbpbi)_2_]^2+^/[Cu(nbpbi)_2_]^+^	N3	14.5	690	49	4.99
[Cu(npbi)_2_]^2+^/[Cu(npbi)_2_]^+^	N719	8.8	750	48	3.19
[Cu(npbi)_2_]^2+^/[Cu(npbi)_2_]^+^	N3	8.8	740	50	3.26

## Other first row M^*n*+^/M^*m*+^ redox mediators

While Co^3+^/Co^2+^ and Cu^2+^/Cu^+^ redox couples have been investigated in detail as alternatives to I_3_^−^/I^−^, the first row of the d-block offers a number of other redox-active metals among which V, Mn, Fe and Ni have received some attention.

### VO^3+^/VO^2+^

**Scheme 10:**
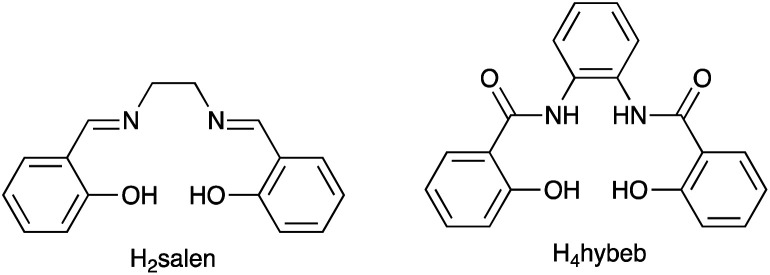
Structures of the conjugate acids of the tetradentate ligands [salen]^2−^ and [hybeb]^4−^.

**Scheme 11:**
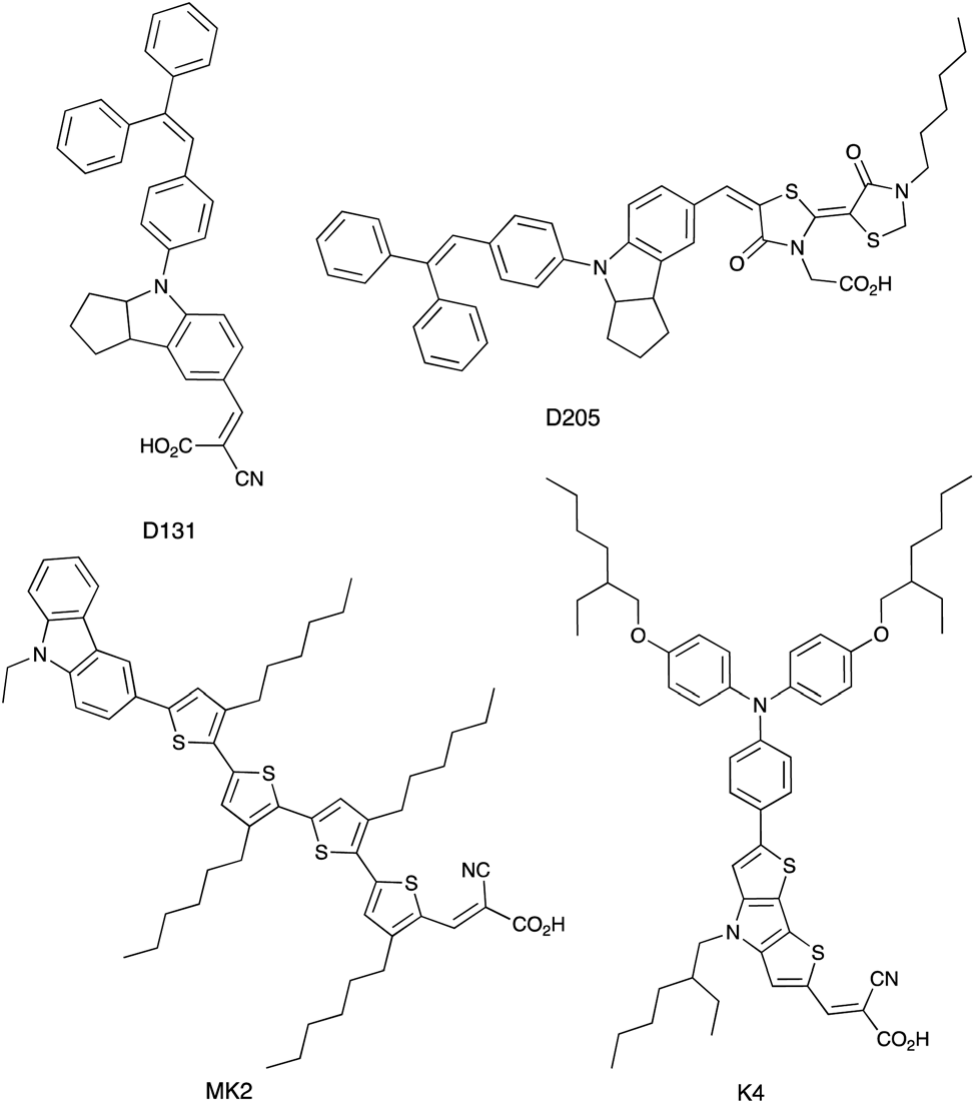
Structures of the commercially available dyes D131 and D205, and of the metal-free dyes MK2 and K4.

Vanadium-based redox mediators have not often been employed in DSCs, but some rather promising results have been reported using oxidovanadium(v/iv) species. The first example from Oyaizu *et al.* in 2013 was motivated in part by the high solubility of [VO(salen)] (see Scheme 10 for H_2_salen) in MeCN. To generate the [VO(salen)]^+^/[VO(salen)] redox mediator, the oxidized form was prepared by aerobic oxidation of [VO(salen)] in the presence of CF_3_SO_3_H to give [VO(salen)(O_3_SCF_3_)]. The single crystal structure of the latter confirms a 6-coordinate vanadium(v) complex. The redox potential for the [VO(salen)]^+^/[VO(salen)] couple (+0.64 V *vs.* Ag/AgCl) is *ca.* 0.3 V more positive than that of I_3_^−^/I^−^. DSCs combining the dyes D131 and D205 (Scheme 11) with [VO(salen)]^+^/[VO(salen)] as the redox mediator were fabricated with and without the co-adsorbent cheno. The value of *E*_redox_ and fast electron transfer kinetics contributed to the promising performance of the DSCs. In the presence of cheno to suppress recombination processes, values of *J*_SC_ = 12.3 mA cm^−2^, *V*_OC_ = 740 mV, ff = 59% and *η* = 5.4% were realized.^139^

In 2015, Apostolopoulou *et al.* reported the use of the [VO(hybeb)]^−^/[VO(hybeb)]^2−^ redox shuttle (for H_4_hybeb, see Scheme 10), with the complexes present as [PPh_4_]^+^ salts. Preliminary experimental and computational studies revealed that the complexes exhibited high rates of electron exchange and transfer, and that the values of *E*_redox_ and the ground state energy level of the dye N719 are well matched for dye regeneration. After optimization of the initial concentrations of [Ph_4_P][VO(hybeb)] and [Ph_4_P]_2_[VO(hybeb)], the best performing DSC under illumination of 1000 W m^−2^ achieved a PCE of 2%, with cell parameters of *J*_SC_ = 5.2 mA cm^−2^, *V*_OC_ = 660 mV, ff = 58%.^140^

While vanadium-based redox mediators have gained minimal attention, the results that are available in the literature demonstrate promise. However, to the best of our knowledge, the long-term stability of these electrolyte components remains untested.

### Mn^4+^/Mn^3+^ and Mn^3+^/Mn^2+^

The range of oxidation states offered by manganese makes it an attractive target for use in redox mediators. In addition, it is abundant in the Earth's crust (*ca.* 950 ppm)^27^ and has a low toxicity. The first application in DSCs came from Spiccia and coworkers who employed the [Mn(acac)_3_]^+^/[Mn(acac)_3_] (Hacac = pentane-2,4-dione) couple in devices sensitized with the ruthenium(ii) dye N719 or the organic dyes MK2 and K4 (Scheme 11). Different fabrications of counter electrode were tested: thermally decomposed Pt/FTO, sputter-coated Pt/FTO, sputter-coated Au/FTO and PEDOT/FTO. PEDOT/FTO electrodes were found to be most compatible with the [Mn(acac)_3_]^+^/[Mn(acac)_3_] redox shuttle. The composition of the electrolyte was optimized to an MeCN solution containing [Mn(acac)_3_] (0.50 M), NOBF_4_ (0.10 M), TBP (1.20 M), LiBF_4_ (0.05 M) and cheno (0.01 M). Note that the co-adsorbent cheno was added to the electrolyte rather than the dye bath; compare this with, for example, the use of cheno with copper(i) and iron(ii) dyes discussed in later sections. Under a light intensity of 1000 W m^−2^, values of *η* for DSCs sensitized with N719, MK2 and K4 were 4.4 ± 0.2%, 4.4 ± 0.2% and 3.9 ± 0.1%, respectively. The three dyes produced values of *J*_SC_ in the range 7.8–8.6 mA cm^−2^, and values of *V*_OC_ in the range 733–771 mV. Good fill-factors (69–73%) contributed to respectable DSC performances. Although the results confirmed the compatibility of the [Mn(acac)_3_]^+^/[Mn(acac)_3_] redox mediator with both ruthenium(ii) and organic dyes, the electron lifetimes of these DSCs were shorter than those for analogous cells containing I_3_^−^/I^−^ and [Co(bpy)_3_]^3+^/[Co(bpy)_3_]^2+^ redox shuttles. This reveals faster electron recombination at the photoanode for the [Mn(acac)_3_]^+^/[Mn(acac)_3_] couple leading to lower PCEs.^141^ Carli *et al.* investigated the use of other [Mn(β-diketonate)_3_]^+^/[Mn(β-diketonate)_3_] redox mediators. Their results demonstrated that although TBP is a critical additive to the electrolyte to suppress charge recombination, the manganese complexes are unstable with respect to ligand exchange with TBP.^142,143^

**Fig. 5:**
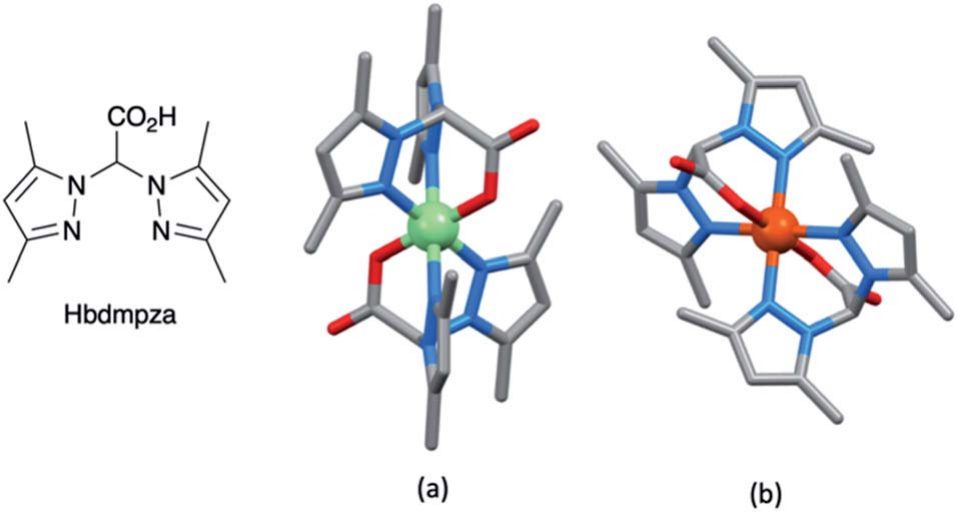
The conjugate acid of the heteroscorpionate ligand [bdmpza]^−^ and the structures of (a) the manganese(iii) complex [Mn(bdmpza)_2_]^+^ (CSD refcode ITEQOP) and (b) the iron(iii) complex [Fe(bdmpza)_2_]^+^ (refcode ITEQEF) both in the [BF_4_]^−^ salts.

A series of octahedral [M(bdmpza)_2_][BF_4_] and [M(bdmpza)_2_] complexes in which M = Mn, Fe and Co, and Hbdmpza is the heteroscorpionate ligand shown in Fig. 5 has also been screened for use in redox mediators. The structure of the [Mn(bdmpza)_2_]^+^ cation is shown in Fig. 5a. Although electrochemical properties of the complexes appeared promising, the solubilities of the Mn(ii) compounds were low in polar solvents.^144^ We return to the iron complexes in the next section.

Before closing this section of manganese-based redox mediators, we note that [Mn(HBpz_3_)_2_]^+^/[Mn(HBpz_3_)_2_] ([HBpz_3_]^−^ = hydridotris(pyrazolyl)borate) and several alkylated derivatives have been applied as redox mediators in quantum dot solar cells.^145^

### Fe^3+^/Fe^2+^

**Scheme 12:**
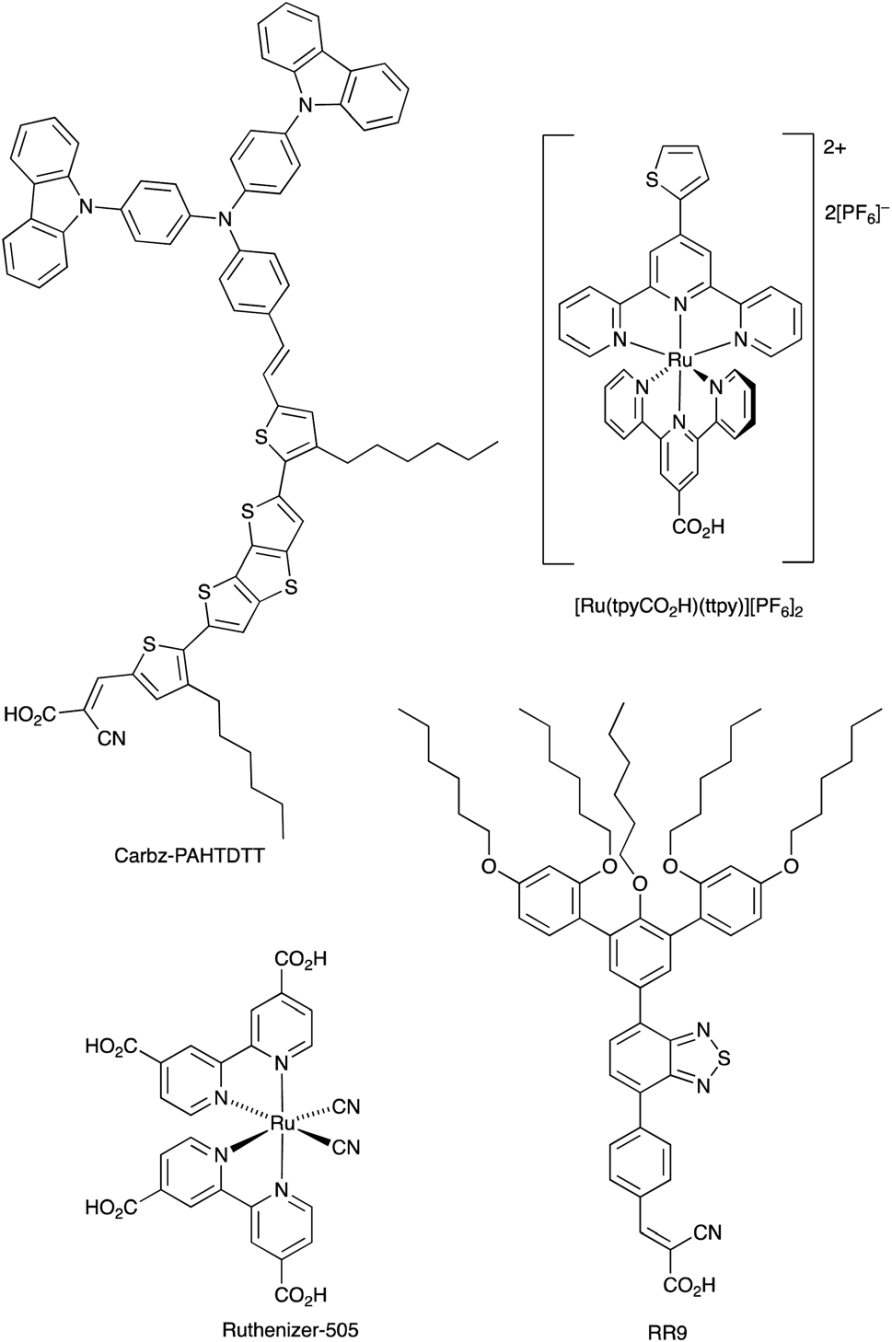
Structures of the metal-free dyes Carbz-PAHTDTT and RR9, and the ruthenium(ii) dyes [Ru(tpyCO_2_H)(ttpy)][PF_6_]_2_ (tpy = 2,2′:6′,2′′-terpyridine) and the commercially available Ruthenizer-505.

With its high natural abundance (*ca.* 41 000 ppm of the Earth's crust)^27^ and redox active properties, iron is the dream metal upon which to base DSC redox mediators in addition to DSC sensitizers (see later). An overview of Fe^3+^/Fe^2+^ redox couples in DSCs was given by Pashaei *et al.* in 2015.^71^ The ferrocenium/ferrocene (Cp_2_Fe^+^/Cp_2_Fe, *E*° = +0.62 V *vs.* NHE) couple is a standard reference redox couple in non-aqueous solvents,^146^ but in a DSC, it was found to suffer from rapid recombination of electrons from the semiconductor.^147^ One approach to suppressing this pathway is to passivate the TiO_2_ surface.^148^ For example, surface treatment with MeSiCl_3_ produces a blocking layer of poly(methylsiloxane) and leads to an improvement, but this is offset by slower regeneration of the oxidized dye.^149^ Remarkable progress with the Cp_2_Fe^+^/Cp_2_Fe redox shuttle was made by Bach, Spiccia and coworkers in 2011. They demonstrated that DSCs sensitized with the organic dye Carbz-PAHTDTT (Scheme 12) and containing an electrolyte comprising Cp_2_Fe^+^/Cp_2_Fe and TBP in MeCN, attained PCEs of up to 7.5%. The dye was selected because of its light absorption over a wide visible-wavelength range; best performances are gained with thin TiO_2_ electrodes. The data in Table 4 illustrate the effects of adding the co-adsorbent cheno, and compare the use of Cp_2_Fe^+^/Cp_2_Fe with the standard I_3_^−^/I^−^ redox mediator.^150^ Exclusion of O_2_ from the DSCs is essential when the Cp_2_Fe^+^/Cp_2_Fe couple is employed, and this makes cell fabrication less convenient than with many other redox mediators. Just as the redox potentials of the Co^3+^/Co^2+^ and Cu^2+^/Cu^+^ couples can be tuned by choice of ligand (see earlier), an advantage of the Cp_2_Fe^+^/Cp_2_Fe couple is that the redox potential can readily be shifted to higher or lower potentials through functionalization of the cyclopentadienyl rings.

**Table 4:** DSC performances using dye Carbz-PAHTDTT (under a light intensity of 1000 W m^−2^) from the work of Bach, Spiccia and coworkers.^150^ The electrolytes contained TBP. DSCs do not appear to be masked

Redox couple	Co-adsorbent	*J*_SC_/mA cm^−2^	*V*_OC_/mV	ff/%	*η*/%
Cp_2_Fe^+^/Cp_2_Fe	cheno	12.2	842	73	7.5
Cp_2_Fe^+^/Cp_2_Fe	—	9.6	815	75	5.9
I_3_^−^/I^−^	cheno	12.3	742	67	6.1
I_3_^−^/I^−^	—	13.3	735	62	6.1

Tris(2,2′-bipyridine)iron(iii)/(ii) based couples have been investigated both as redox mediators and co-mediators, the [Fe(bpy)_3_]^3+^/[Fe(bpy)_3_]^2+^ couple being readily reversible and stable with respect to ligand dissociation. In 2010, Caramori, Gros and coworkers demonstrated the use of [Fe(4,4′-Me_2_bpy)_3_]^3+^/[Fe(4,4′-Me_2_bpy)_3_]^2+^ and [Fe(4,4′-(MeO)_2_bpy)_3_]^3+^/[Fe(4,4′-(MeO)_2_bpy)_3_]^2+^ redox co-mediators in conjunction with [Co(4,4′-^*t*^Bu_2_bpy)_3_]^3+^/[Co(4,4′-^*t*^Bu_2_bpy)_3_]^2+^. Compared to DSCs using only the cobalt-based redox mediator, the electron-collection efficiency of a DSC sensitized with the ruthenium complex [Ru(tpyCO_2_H)(ttpy)][PF_6_]_2_ (Scheme 12) was enhanced when [Fe(4,4′-Me_2_bpy)_3_]^3+^/[Fe(4,4′-Me_2_bpy)_3_]^2+^ was added as a co-mediator. The improvement has its origins in electron-transfer between the Co^3+^/Co^2+^ and Fe^3+^/Fe^2+^ couples which creates an electron cascade between oxidized dye, electron co-mediator and electron mediator.^151^ As Fig. 2a and 3 illustrated, high values of *V*_OC_ are achieved by careful tuning of *E*_redox_, and by judicious matching of dye and redox couple energy levels. Earlier, we described the efficient combination of [Co(bpy)_3_]^3+^/[Co(bpy)_3_]^2+^ with the donor–π-bridge–acceptor triphenylamine dye D35 (Scheme 4).^85^ Starting with D35, Delcamp and coworkers^152^ designed dye RR9 (Scheme 12) to be energetically compatible with the [Fe(bpy)_3_]^3+^/[Fe(bpy)_3_]^2+^ redox mediator. Note that the arylamine group in D35 was replaced by an aryl-centred unit in RR9 to achieve a lower energy ground-state oxidation potential. Upon going from a combination of D35 and [Co(bpy)_3_]^3+^/[Co(bpy)_3_]^2+^ to RR9 and [Fe(bpy)_3_]^3+^/[Fe(bpy)_3_]^2+^, the maximum theoretical increase in *V*_OC_ is 810 mV. In practice, DSCs with these dye–redox couple combinations achieved values (average for two cells) of *V*_OC_ of 760 and 1420 mV, respectively. TiO_2_ layer thickness (2.7 μm) proved critical. For masked DSCs with RR9 and [Fe(bpy)_3_]^3+^/[Fe(bpy)_3_]^2+^, average values of *J*_SC_ = 2.8 mA cm^−2^, ff = 47%, and *η* = 1.9% were reported. This work is also of note for the fabrication of sequential series multijunction (SSM)-DSCs based upon RR9 and [Fe(bpy)_3_]^3+^/[Fe(bpy)_3_]^2+^ which attained single-illuminated-area voltages of 3.34 V from a three-subcell system.^152^

Potentials for redox mediators based on [Fe(bpy)_3_]^3+^/[Fe(bpy)_3_]^2+^ can be adjusted through ligand functionalization. Another approach is to move to couples based on [Fe(tpy)_2_]^3+^/[Fe(tpy)_2_]^2+^ and its derivatives. An example comes from the work of Kozyukhin *et al.* in which DSCs containing the commercial dye Ruthenizer-505 (Scheme 12) and the redox mediator [Fe(pytpy)_2_]^3+^/Fe(pytpy)_2_]^2+^ (pytpy = 4′-(pyridin-4-yl)-2,2′:6′,2′′-terpyridine) were tested under illumination of 1000 W m^−2^, and compared with analogous DSCs using an I_3_^−^/I^−^ redox shuttle. The *J*–*V* characteristics of the Fe-based electrolyte were notably poorer than those with I_3_^−^/I^−^, and this was explained in terms of the slower reduction kinetics of the oxidized dye for the [Fe(pytpy)_2_]^3+^/Fe(pytpy)_2_]^2+^ mediator.^153^

Although there has been progress with the use of Cp_2_Fe^+^/Cp_2_Fe and [Fe(bpy)_3_]^3+^/[Fe(bpy)_3_]^2+^-based redox couples in DSCs, there is significant scope for further exploration and improvements. Among the other classes of iron complexes considered for potential redox mediators are those with scorpionate ligands, *i.e.* tridentate (tripodal) ligands which lead to metal complexes with high stability constants. Such ligands are preorganized to bind to a metal ion in a *fac*-mode, as in [Fe(bdmpza)_2_]^+^ (Fig. 5b). Burzlaff and coworkers reported that [Fe(bdmpza)_2_][BF_4_] exhibits a reversible (iron-centered) redox process at +0.46 V *vs.* NHE. Despite the redox potential comparing favourably with that of the I_3_^−^/I^−^ couple, the solubilities of the iron complexes in solvents typically used in DSCs were too low for practical applications.^144^ This underlines one of the difficulties of pinpointing appropriate redox mediators for DSCs. While out of the main remit of this review, it is pertinent to note that the [Fe(acac)_3_]/[Fe(acac)_3_]^−^ redox mediator has been successfully developed for use in p-type DSCs.^154^

### Ni^4+^/Ni^3+^

**Fig. 6:**
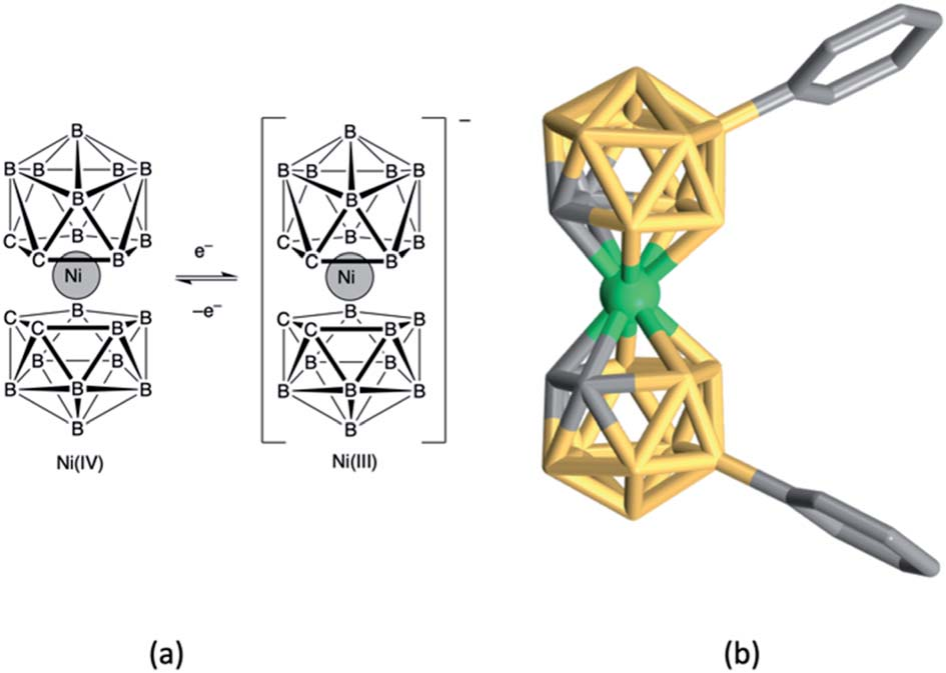
(a) The nickel(iv)/(iii) bis(dicarbollide), [Ni(C_2_B_9_H_11_)_2_]/[Ni(C_2_B_9_H_11_)_2_]^−^, redox couple, and (b) the structure of the nickel(iv) complex [Ni(C_2_B_9_H_10_Ph)_2_] (CSD refcode HABQEI).

Nickel(iv)/(iii) bis(dicarbollide) complexes show high thermal stability, are non-corrosive and interconvert reversibly (Fig. 6a) making them suitable candidates for redox mediators in DSCs,^155^ with redox potentials tuned by introducing electron-donating or withdrawing substituents. Hupp and coworkers fabricated DSCs sensitized with N719 and incorporating electrolytes comprising [Ni(C_2_B_9_H_10_R)_2_] (R = Ph, 4-MeC_6_H_4_, 4-MeOC_6_H_4_, 4-ClC_6_H_4_, 4-CF_3_C_6_H_4_, 3,5-(CF_3_)_2_C_6_H_3_), [Ni(C_2_B_9_H_10_R)_2_]^−^, [Bu_4_N][BF_4_] and TBP in dichloroethane. Values of *V*_OC_ in the range 640–740 mV were observed which could be enhanced by atomic layer deposition of Al_2_O_3_ (*ca.* 1.1 Å) on the TiO_2_ surface of the photoanode.^156^ There appears to have been no further development of this type of Ni-based redox mediator.

## First row d-block metals: from redox mediators to dyes

So far, we have focused on the highly promising approaches to replacing the I_3_^−^/I^−^ redox couple by mediators based upon first row d-block metals, in particular cobalt and copper. As we have discussed, some outstanding photoconversion efficiencies have been achieved using Co^3+^/Co^2+^ or Cu^2+^/Cu^+^ couples and metal-free or zinc(ii) porphyrin dyes. Nonetheless, the synthetic complexity associated with many state-of-the-art organic dyes is a disadvantage for upscaling for commercial applications. In contrast, first row d-block metal coordination compounds containing synthetically-accessible ligands and which absorb in the visible region are readily prepared. Complexes of copper(i) and iron(ii) are especially promising candidates for use as sensitizers in DSCs.

In the next part of this review, we turn our attention from redox couples to sensitizers. We have excluded zinc(ii) porphyrin and zinc(ii) phthalocyanine dyes because progress in this extensive field has been thoroughly reviewed,^7,9,30,36,46,48,53–55,157,158^ and references within the following selected papers also serve to access the relevant literature.^31,56,159–173^ More generally, the use of Earth abundant metals in DSCs has been reviewed by Förster and Heinze in 2020.^174^ Our last general review in this area covered the literature up to 2012 ^175^ and therefore the main emphasis in the current article is on developments in the last decade.

## The most readily achieved goal: copper-based sensitizers in DSCs

### From ruthenium(ii) to copper(i)

State-of-the-art ruthenium(ii) dyes are usually based upon an {Ru(bpy)_3_}^2+^ or {Ru(bpy)_2_(NCS)_2_} core. The development of copper(i) dyes which typically incorporate a {Cu(diimine)_2_}^+^ core (diimine = bpy or phen) follows from similarities between their photophysical behaviour and those of [Ru(diimine)_3_]^2+^ complexes.^174,176^ Simple (*e.g.* [BF_4_]^−^, [PF_6_]^−^) salts of [Ru(diimine)_3_]^2+^ and [Cu(diimine)_2_]^+^ species are usually orange or orange-red with absorption maxima in the range 400–550 nm. A notable distinction between them, however, is that molar extinction coefficients of [Cu(diimine)_2_]^+^ complexes are lower (*ca.* 5000 M^−1^ cm^−1^) than those of [Ru(diimine)_3_]^2+^ complexes (*ca.* 15 000 M^−1^ cm^−1^). As we exemplify later, this disadvantage of the copper(i) species can be addressed by appropriate functionalization of the diimine metal-binding domain.

The excited states of both [Ru(diimine)_3_]^2+^ and [Cu(diimine)_2_]^+^ complexes are mainly metal-to-ligand charge-transfer (MLCT) in character, and arise from the excitation of an electron from metal d-orbitals to antibonding π*-orbitals localized on the diimine ligand. There is, however, a significant difference between the ruthenium(ii) and copper(i) compounds. Upon excitation of an octahedral [Ru(diimine)_3_]^2+^ (d^6^) species, there is negligible change in the equilibrium geometry as the metal formally undergoes oxidation from Ru(ii) to Ru(iii). A d^10^ [Cu(diimine)_2_]^+^ complex is tetrahedral (or distorted tetrahedral) and the excited MLCT state is formally a d^9^ copper(ii) species for which a tetragonal arrangement of donor atoms is preferred. Excitation is therefore accompanied by a flattening of the copper coordination sphere and, unless this is mitigated through steric effects (see later), solvent interactions with the Cu(ii) metal centre result in shortening of the excited-state lifetime.

Another important difference between [Ru(diimine)_3_]^2+^ and [Cu(diimine)_2_]^+^ complexes is the lability of the ligands. The d^6^ configuration of ruthenium(ii) leads to a kinetically inert metal centre. In contrast, the d^10^ configuration of copper(i) results in labile ligands which undergo rapid exchange. Thus, for example, the ^1^H NMR spectrum of a 1 : 1 mixture of [Cu(6,6′-Me_2_bpy)_2_][PF_6_] and [Cu(2,9-Me_2_phen)_2_][PF_6_] in CD_3_CN exhibits four signals assigned to the methyl groups arising from a 1 : 2 : 1 statistical mixture of [Cu(6,6′-Me_2_bpy)_2_][PF_6_], [Cu(6,6′-Me_2_bpy)(2,9-Me_2_phen)][PF_6_] and [Cu(2,9-Me_2_phen)_2_][PF_6_].^177^ Ligand lability is a key issue that has been addressed by the ‘surfaces-as-ligands, surfaces-as-complexes’ (SALSAC)^178^ and the heteroleptic 1,10-phenanthroline Cu(i) complexes (HETPHEN)^179^ approaches which are discussed in detail below.

### Development of copper(i) dyes: homoleptic complexes

**Scheme 13:**
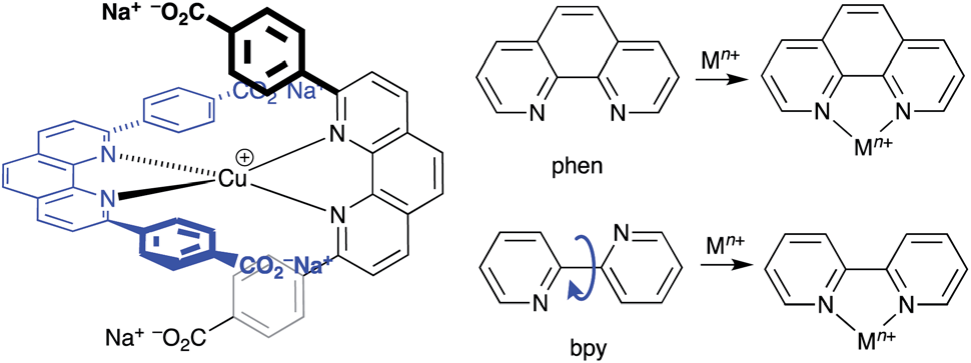
The sensitizer designed by Sauvage and coworkers, and the preorganized nature of the phen metal-binding domain compared to the conformational change required by bpy.

**Fig. 7:**
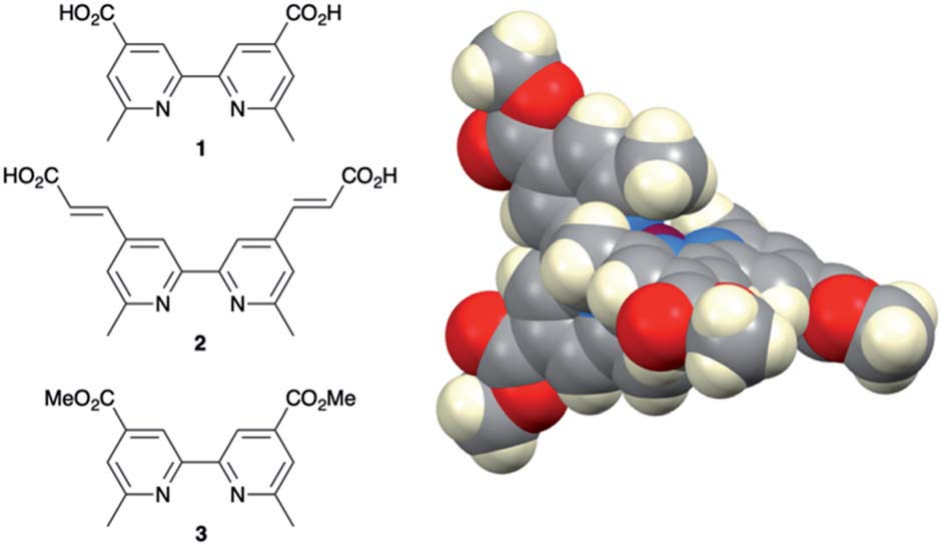
Structures of ligands 1–3, and the structure of [Cu(3)_2_]^+^ in the [PF_6_]^−^ salt (CSD refcode JOHXIO). The space-filling representation is used to emphasize the protection imparted by the 6- and 6′-methyl groups.

A number of reviews provide an entry into the area of bis(diimine)copper(i) sensitizers for DSCs.^174,175,180–183^ In 1994, Sauvage and coworkers were the first to demonstrate the combination of a homoleptic copper(i) complex as a dye with a wide-band-gap semiconductor for photoconversion.^184^ A number of features of their dye (Scheme 13) are relevant for an understanding of the design of ligands for bis(diimine)copper(i) sensitizers. Firstly, the choice of phen rather than bpy is advantageous because the phen metal-binding domain is preorganized for coordination whereas bpy requires a conformation change from s-*trans* to s-*cis* (Scheme 13). Consequently, [Cu(phen)_2_]^+^-based complexes possess stability constants which are typically one or two log *K* units greater than analogous [Cu(bpy)_2_]^+^ complexes. On the other hand, this has to be offset against the fact that a greater range of functionalized bpy ligands is synthetically accessible than functionalized phen ligands. The second feature of the copper(i) complex shown in Scheme 13 is the presence in the phen ligands of sterically demanding 2,9-substituents to prevent flattening of the copper coordination sphere upon excitation (see above). Thirdly, the carboxylate units are introduced to act as anchoring domains to attach the dye to the semiconductor surface. From this milestone report, little progress was made^185^ until 2008 when we, in collaboration with Grätzel, demonstrated photoconversion efficiencies of 1.9 and 2.3%, respectively, for masked DSCs sensitized with [Cu(1)_2_][PF_6_] and [Cu(2)_2_][PF_6_] (see Fig. 7 for ligand structures) and using an I_3_^−^/I^−^ redox mediator. These values of *η* compared to a value of *η* = 9.7% for a reference cell sensitized with the ruthenium(ii) dye N719. Structural data for the related ester [Cu(3)_2_][PF_6_] (Fig. 7) illustrated that the 6- and 6′-methyl groups are sufficiently large to protect the Cu(i) centre.^186^

**Scheme 14:**
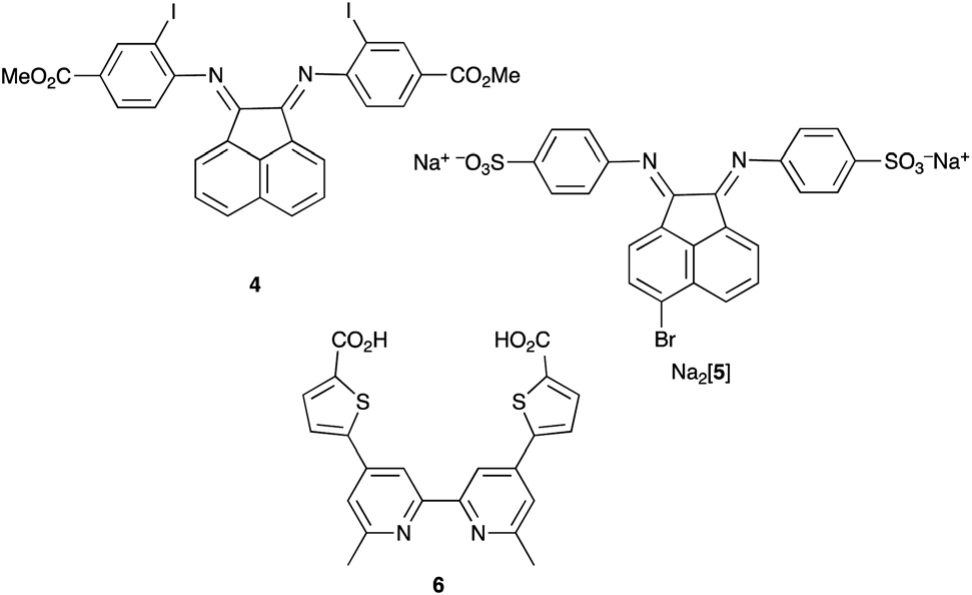
Structures of the bis(arylimino)acenaphthene compounds 4 and Na_2_[5], and ligand 6.

Note that while dyes with alkyl ester functionalities may bind to TiO_2_ as a result of hydrolysis to the corresponding carboxylic acid,^186^ use of the ester-protected anchoring domains is usually detrimental to DSC performance.^187^ In order to overcome this problem, Soo and coworkers pre-treated the TiO_2_-electrodes with a THF solution of KO^*t*^Bu for two days. Attachment of [Cu(4)_2_]^+^ was then possible through reaction of the ester groups in 4 (Scheme 14) with the activated surface. However, a non-optimized DSC containing [Cu(4)_2_]^+^ and the I_3_^−^/I^−^ redox mediator with TBP, GNCS, and DMII additives (GCNS = guanidinium thiocyanate, DMII = 1,3-dimethylimidazolium iodide) in a MeCN/valeronitrile-based electrolyte only achieved a value of *J*_SC_ = 0.0338 mA cm^−2^ and *V*_OC_ = 339 mV. Slight improvement was obtained by using [Cu(5)_2_]^3−^ in which [5]^2−^ (Scheme 14) contains sulfonate anchors.^188^ The effects of structural variation in the anchoring domain are known to have a significant impact on dye performance in DSCs,^41^ and Wills *et al.*^189^ have shown that the introduction of a thienyl spacer between a 6,6′-Me_2_bpy metal-binding domain and a carboxylic acid anchoring group leads to enhanced PCE for DSCs containing the homoleptic dye [Cu(6)_2_]^+^ (see Scheme 14 for ligand 6) compared to the previously reported [Cu(1)_2_]^+^.^186^

In 2013, Wills *et al.* prepared the 2,2′-biquinoline (biq) derivative [Et_3_NH][Cu(4,4′-(HO_2_C)_2_biq)(4,4′-(O_2_C)_2_biq)]. The extended conjugation with respect to related bpy complexes leads to the absorption maximum extending to longer wavelengths. In solution *λ*_max_ = 564 nm, and for the dye adsorbed in TiO_2_, *λ*_max_ = 552 nm. DSCs sensitized using [Et_3_NH]^+^ or Na^+^ salts of [Cu(4,4′-(HO_2_C)_2_biq)(4,4′-(O_2_C)_2_biq)]^−^ or with [Cu(4,4′-(HO_2_C)_2_biq)_2_]^+^ coupled with an I_3_^−^/I^−^ redox mediator were tested. Values of *J*_SC_ and *V*_OC_ were in the ranges of 0.197–0.235 mA cm^−2^ and 499–629 mV, respectively, leading to PCEs ≤ 0.1%. Removing TBP from the electrolyte did not lead to enhancement of DSC performance.^190^ A possible reason for these low performances is the short excited-state lifetime caused by exciplex formation.^191^

### From homoleptic to heteroleptic complexes: the SALSAC approach

**Fig. 8:**
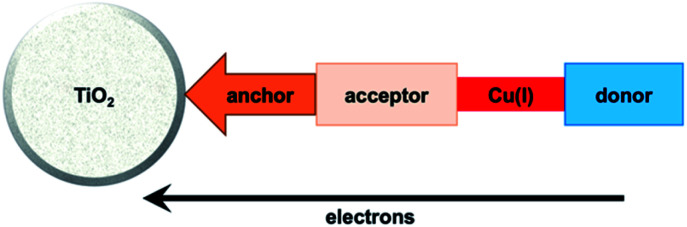
A schematic representation of structural design for a heteroleptic bis(diimine)copper(i) sensitizer facilitating electron transfer through the dye molecule to the n-type semiconductor.

**Scheme 15:**
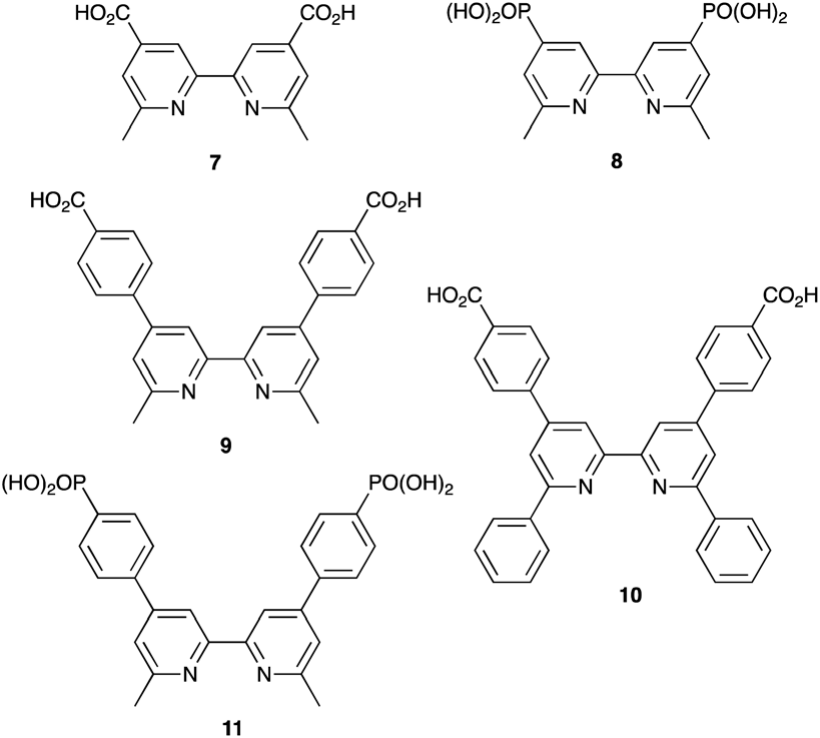
Structures of functionalized bpy ligands 7–11 which contain carboxylic or phosphonic acid anchoring groups.

**Fig. 9:**
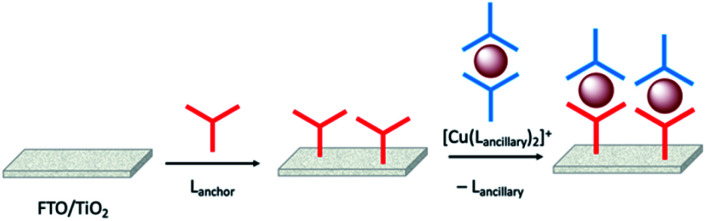
The SALSAC approach to *in situ* assembly of a heteroleptic copper(i) dye on an electrode surface using ligand exchange.

Enhancement of the photoconversion efficiency of DSCs based upon homoleptic copper(i) complexes is limited^175,180^ because of the lack of the desired ‘push–pull’ effect which is the key to the success of donor–π-bridge–acceptor metal-free dyes (see earlier discussion). To improve the performance of homoleptic copper(i) dyes, one strategy is to optimize the electrolyte components.^192^ A second strategy of using surface-bound heteroleptic dyes allows broad scope for capitalizing upon structure–property relationships. Fig. 8 illustrates the components of a heteroleptic copper(i) dye that should be present to facilitate electron transfer across the dye from the redox shuttle to semiconductor. In 2008, we established a protocol for ligand exchange reactions between [Cu(N^N)_2_][PF_6_] (N^N = functionalized bpy ligand) and TiO_2_-anchored ligands,^177^ and later screened a wider range of anchoring ligands to 7–10 (Scheme 15).^193^ The dye assembly process involves ligand exchange between a surface-anchored diimine ligand, L_anchor_, and a homoleptic complex [Cu(L_ancillary_)_2_]^+^ in the dye-bath (Fig. 9). This is the basis of the SALSAC approach which has also been developed to include stepwise assembly of the dye.^178^ The strategy takes advantage of the lability of bis(diimine)copper(i) complexes in solution. Critically, once attached to the semiconductor surface, the heteroleptic complex does not suffer from rapid ligand dissociation. The formation of surface-bound heteroleptic complexes was confirmed by using MALDI-TOF mass spectrometry and solid-state absorption spectroscopy. While not achieving PCEs greater than 1.51% for unmasked DSCs, our investigation in 2011 was pivotal in revealing that, for copper(i) dyes, phosphonic acid anchoring groups were more beneficial than carboxylic acids.^193^ We later showed that the presence of a phenylene spacer in anchoring ligand 11 (Scheme 15) enhanced DSC performance with respect to analogous DSCs containing dyes incorporating anchoring ligand 10.^194^ The observed benefits of using phosphonic rather than carboxylic acid anchors are consistent with the known adsorption strength of a phosphonic acid on TiO_2_ being *ca.* 80 times greater than that of a carboxylic acid.^195^

**Scheme 16:**
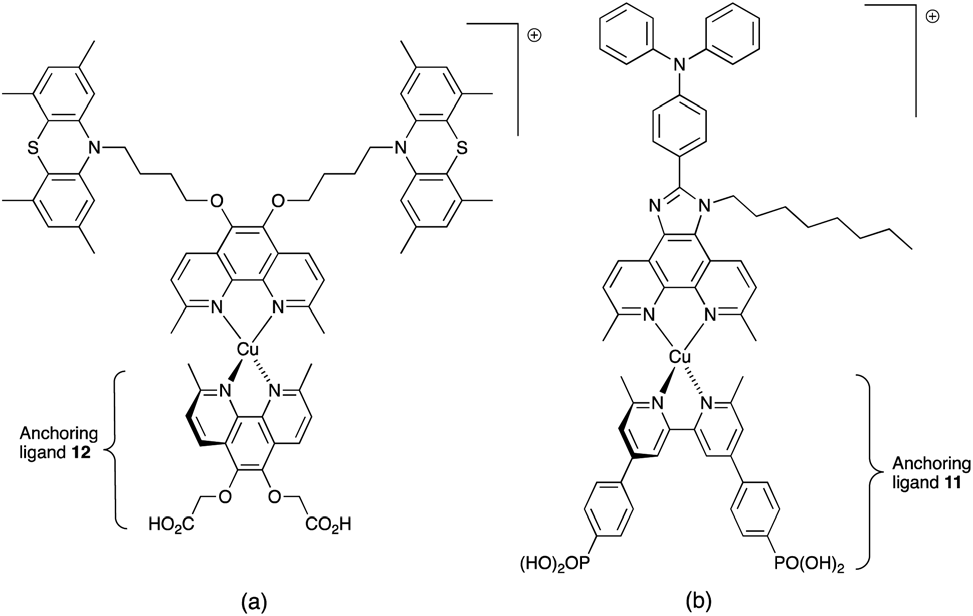
The structures of (a) [Cu(12)(tmpDMP)]^+^ reported by Ashbrook and Elliott, and (b) a copper(i) dye reported by our group tested with a Co^3+^/Co^2+^ redox mediator. Both heteroleptic dyes were assembled *in situ* using the SALSAC approach.

In 2013, Ashbrook and Elliott reported a stepwise assembly of heteroleptic copper(i) complexes of which [Cu(12)(tmpDMP)]^+^ (Scheme 16a) is representative, coupled with the use of a [Co(4,4′-^*t*^Bu_2_bpy)_3_]^3+^/[Co(4,4′-^*t*^Bu_2_bpy)_3_]^2+^ redox mediator.^196,197^ Around the same time, we also showed that a [Co(bpy)_3_]^3+^/[Co(bpy)_3_]^2+^ redox couple could replace I_3_^−^/I^−^ with no loss in DSC performance.^198^ Both studies used heteroleptic copper(i) dyes (Scheme 16) and were important in establishing the compatibility of copper(i) dyes and cobalt-based redox shuttles, paving the way for a shift away from the I_3_^−^/I^−^ mediator. In the work from Ashbrook and Elliott, a DSC containing [Cu(12)(tmpDMP)]^+^ performed the best in the series, but achieved only a modest value of *J*_SC_ (0.54 mA cm^−2^ compared to 1.74 mA cm^−2^ for a reference DSC with the ruthenium dye N3).^196,197^ The performances of masked DSCs sensitized by the dye shown in Scheme 16b combined with a [Co(bpy)_3_]^3+^/Co(bpy)_3_]^2+^ redox mediator were influenced by the thickness of the TiO_2_ layer on the photoanode and by post-treatment with H_2_O–TiCl_4_. The highest value of *J*_SC_ obtained was 5.11 mA cm^−2^ which contributed to *η* = 2.08% (compared to *η* = 6.90% for a DSC with N719 and I_3_^−^/I^−^). Note that the design of ancillary ligand in the complex in Scheme 16b incorporates an electron-donating Ph_2_N unit and long alkyl chain to inhibit electron recombination.^198^ We return to similar dyes later. Ashbrook and Elliott also demonstrated that Lewis bases in the [Co(4,4′-^*t*^Bu_2_bpy)_3_]^3+^/[Co(4,4′-^*t*^Bu_2_bpy)_3_]^2+^-based electrolyte (*e.g.* TBP or solvent) interact with the oxidized form of the surface-bound dye, inhibiting dye regeneration. They noted that this can be circumvented by careful choice of the ancillary ligand. When the dye was [Cu(12)(tmpDMP)]^+^, the 2,4,6,8-tetramethylphenothiazine groups in the ancillary ligand rapidly reduce the oxidized dye, thus precluding it from being kinetically trapped by coordination of Cu(ii) with TBP.^196,197^ Despite the promising performances achieved using combinations of bis(diimine)copper(i) dyes and Co^3+^/Co^2+^ redox mediators, the vast majority of DSC investigations involving copper(i) dyes continue to employ the I_3_^−^/I^−^ couple. We shall discuss the role of Cu^2+^/Cu^+^ mediators in a later section.

**Scheme 17:**
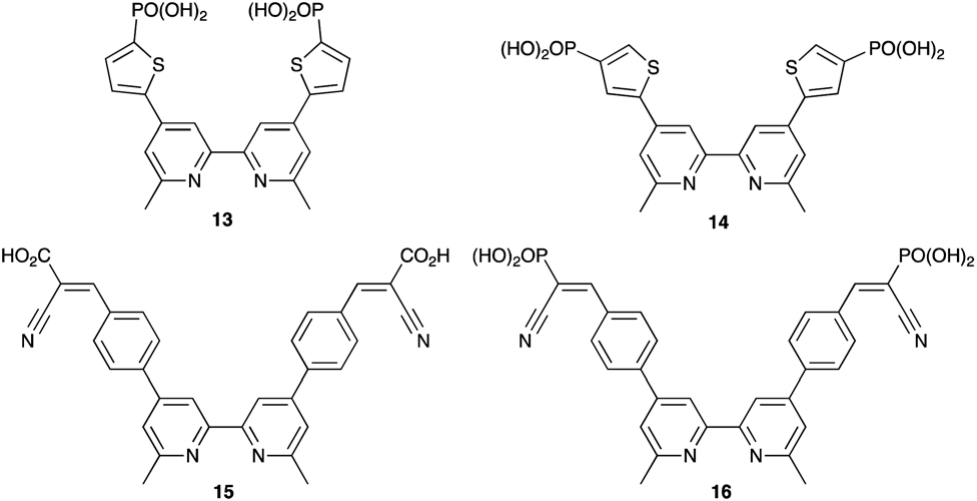
Structures of some anchoring ligands used with heteroleptic copper(i) dyes. See also Scheme 15.

By using the SALSAC approach, it is possible to screen a wide range of [Cu(L_anchor_)(L_ancillary_)]^+^ sensitizers containing different ancillary ligands with a common anchoring domain, or a range of anchoring ligands with a common L_ancillary_. As already detailed, we have found that for copper(i) dyes, ligand 11 is the anchoring ligand of choice. The protonation state of the ligand adsorbed on the TiO_2_ surface remains undefined, although the addition of one equivalent of base during surface functionalization with 11 can lead to an increase in DSC efficiency. On the other hand, addition of ≥3 equivalents of base results in poorer DSC performances.^199^ Replacing the phenylene spacers in 11 by 2-thienyl spacers bearing the phosphonic acid in the 5- or 4-positions (ligands 13 and 14, Scheme 17) leads to slight performance enhancement, and *V*_OC_ is higher when the PO(OH)_2_ group is in the 4- rather than 5-position.^200^ However, the improvement in PCE is offset by the easier synthetic route to 11 compared to the thienyl derivatives. Similarly, there is no benefit to replacing the PO(OH)_2_ groups in 11 by cyanoacrylic acid or (1-cyanovinyl)phosphonic acid anchors (ligands 15 and 16, Scheme 17).^201^

**Scheme 18:**
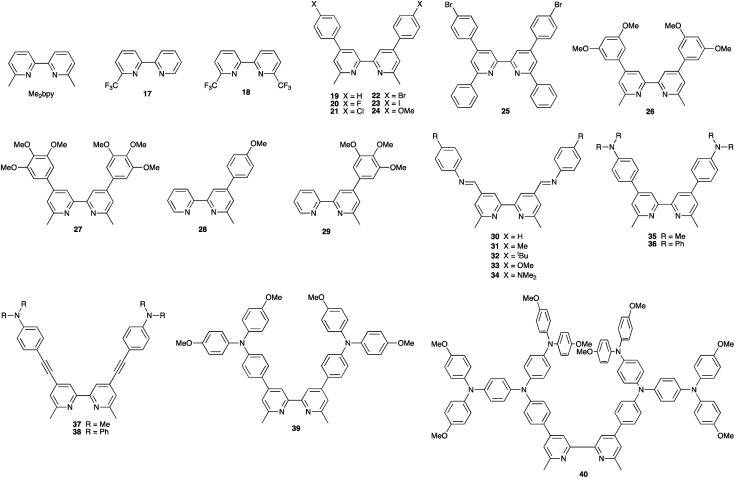
Structures of ancillary ligands which are derivatives of bpy used in complexes in Table 5. Note the use of electron-releasing methoxy groups in some of the ligands (see text).

**Scheme 19:**
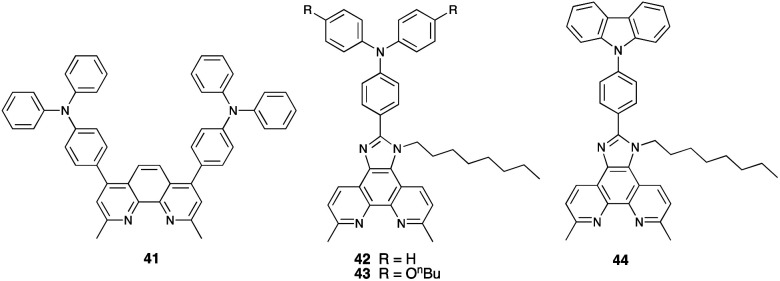
Structures of ancillary ligands which are derivatives of phen used in complexes in Table 5.

**Scheme 20:**
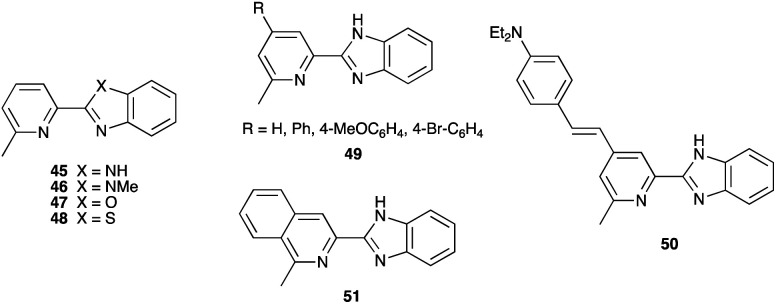
Structures of a series of related ancillary ligands used in heteroleptic copper(i) dyes; complexes with ligands in the family 49–51 were the subject of a theoretical study (see text).

Table 5 presents DSC parameters for a wide range of heteroleptic copper(i) dyes in which the anchoring ligand is 11 and the ancillary ligands are defined in Schemes 18–20. So that comparisons are legitimate, we have only included data for DSCs which were fabricated in a similar manner, in which DSCs were fully masked, and in which the electrolyte comprised LiI (0.1 M), I_2_ (0.05 M), 1-methylbenzimidazole (0.5 M) and 1-butyl-3-methylimidazolinium iodide (0.6 M) in 3-methoxypropionitrile (MPN). In most cases, the solvent in the dye bath for the ligand exchange process (Fig. 9) was CH_2_Cl_2_ (column 2 in Table 5). So that the photoconversion efficiencies can be compared for different investigations, values of *η* are accompanied in Table 5 by values relative to *η* for a reference DSC sensitized by N719. This is especially important when DSC performances are measured using different sun simulators (see footnotes *b* and *c* in Table 5) where absolute values of *J*_SC_, *V*_OC_, and *η* may differ, but the relative values of *η* are comparable.^187^ This is evident from the first two entries in Table 5 for DSCs sensitized with [Cu(11)(Me_2_bpy)]^+^, and for the DSCs containing [Cu(11)(22)]^+^. All parameters in Table 5 refer to the performances recorded on the day on which DSCs were fabricated. Where parameters for multiple cells were reported for a given dye in the original publications, the best performing DSC from each set is given in Table 5.

**Table 5:** DSC (masked cells) parameters for heteroleptic copper(i) dyes assembled using the SALSAC strategy in which the anchoring ligand is 11 (Scheme 15). See text for electrolyte composition. DSCs were illuminated under a light intensity of 1000 W m^−2^

Dye	Solvent in dye bath and dipping time	*J*_SC_/mA cm^−2^	*V*_OC_/mV	ff/%	*η*/%	*η*_rel_/% (relative to N719)^a^	Ref.
[Cu(11)(Me_2_bpy)]^+^	CH_2_Cl_2_ (*ca.* 3 days)	5.55^b^	522	72.0	2.08	27.9	187
[Cu(11)(Me_2_bpy)]^+^	CH_2_Cl_2_ (*ca.* 3 days)	3.79^c^	522	73.8	1.46	24.7	187
[Cu(11)(17)]^+^	CH_2_Cl_2_ (3 days)	5.35^c^	530	73.4	2.08	34.8	202
[Cu(11)(18)]^+^	CH_2_Cl_2_ (3 days)	4.81^c^	537	73.5	1.90	31.8	202
[Cu(11)(19)]^+^	CH_2_Cl_2_ (3 days)	4.27^c^	545	71	1.66	28.7	203
[Cu(11)(20)]^+^	CH_2_Cl_2_ (*ca.* 3 days)	6.37^b^	544	70.0	2.42	33.9	204
[Cu(11)(21)]^+^	CH_2_Cl_2_ (*ca.* 3 days)	6.00^b^	522	69.5	2.18	30.6	204
[Cu(11)(22)]^+^	CH_2_Cl_2_ (*ca.* 3 days)	6.49^b^	525	71.7	2.45	33.0	204
[Cu(11)(22)]^+^	CH_2_Cl_2_ (3 days)	4.92^c^	554	71.7	1.95	33.0	205
[Cu(11)(22)]^+^	MeCN (4 days)	5.09^b^	497	72	1.82	27.5	194
[Cu(11)(23)]^+^	CH_2_Cl_2_ (*ca.* 3 days)	6.92^b^	576	72.6	2.89	38.9	204
[Cu(11)(24)]^+^	CH_2_Cl_2_ (3 days)	4.87^c^	528	71	1.82	30.1	203
[Cu(11)(25)]^+^	CH_2_Cl_2_ (3 days)	4.63^c^	550	74.3	1.89	32.0	205
[Cu(11)(26)]^+^	CH_2_Cl_2_ (3 days)	3.68^c^	528	73	1.43	25.4	203
[Cu(11)(27)]^+^	CH_2_Cl_2_ (3 days)	4.79^c^	567	72	1.96	33.9	203
[Cu(11)(28)]^+^	CH_2_Cl_2_ (3 days)	4.96^c^	583	73	2.12	38.4	206
[Cu(11)(29)]^+^	CH_2_Cl_2_ (3 days)	4.33^c^	571	74	1.83	33.2	206
[Cu(11)(30)]^+^	CH_2_Cl_2_ (3 days)	4.08^c^	538	69	1.51	26	207
[Cu(11)(31)]^+^	CH_2_Cl_2_ (3 days)	3.44^c^	524	70	1.26	22	207
[Cu(11)(32)]^+^	CH_2_Cl_2_ (3 days)	4.08^c^	522	68	1.45	25	207
[Cu(11)(33)]^+^	CH_2_Cl_2_ (3 days)	2.96^c^	516	71	1.08	19	207
[Cu(11)(34)]^+^	CH_2_Cl_2_ (3 days)	1.56^c^	455	65	0.46	8	207
[Cu(11)(35)]^+^	CH_2_Cl_2_ (3 days)	4.74^c^	539	70.1	1.79	33.4	208
[Cu(11)(36)]^+^	CH_2_Cl_2_ (3 days)	5.25^c^	523	70.3	1.93	36.0	208
[Cu(11)(37)]^+^	CH_2_Cl_2_ (3 days)	3.59^c^	514	70.7	1.30	24.3	208
[Cu(11)(38)]^+^	CH_2_Cl_2_ (3 days)	4.24^c^	535	69.3	1.57	29.3	208
[Cu(11)(39)]^+^	CH_2_Cl_2_ (*ca.* 3 days)	5.47^b^	490	69.4	1.86	25.1	209
[Cu(11)(40)]^+^	CH_2_Cl_2_ (*ca.* 3 days)	4.32^b^	509	68.1	1.50	20.3	209
[Cu(11)(41)]^+^	MeCN (3 days)	6.81^b^	557	72	2.73	33.9	210
[Cu(11)(42)]^+^	MeCN (3 days)	5.41^b^	562	75	2.29	28.4	210
[Cu(11)(43)]^+^	MeCN (3 days)	5.65^b^	540	68	2.07	25.7	210
[Cu(11)(44)]^+^	MeCN (3 days)	5.89^b^	562	72	2.38	29.5	210
[Cu(11)(45)]^+^	CH_2_Cl_2_ (3 days)	6.93^b^	608	71.9	3.03	40.1	211
[Cu(11)(46)]^+^	CH_2_Cl_2_ (3 days)	6.99^b^	558	69.5	2.71	35.9	211
[Cu(11)(47)]^+^	CH_2_Cl_2_ (3 days)	6.91^b^	531	71.4	2.62	34.7	211
[Cu(11)(48)]^+^	CH_2_Cl_2_ (3 days)	7.76^b^	530	69.9	2.88	38.1	211

a For each dye, the PCE was measured relative to a reference cell with N719.

b Light source = Solaronix SolarSim 150 (1000 W m^−2^).

c Light source = LOT Quantum Design LS0811 (1000 W m^−2^).

The simplest ancillary ligand in Scheme 18 and Table 5 is Me_2_bpy. The 6,6′-dimethyl substituents are primarily introduced to hinder flattening of the copper coordination sphere upon photoexcitation (see earlier discussion). The sensitizer [Cu(11)(Me_2_bpy)]^+^ can therefore be taken as a reference point to illustrate how DSC performance can be improved as a consequence of structural modification of the ancillary ligand. To achieve the donor–acceptor properties of the dye (Fig. 8), the ancillary ligand should possess electron-releasing functionalities, and indeed, this is true for most of the ligands ancillary ligands shown in Schemes 18 and 19. However, compounds 17, 18 and 20–25 (Scheme 18) might not be expected to be target ligands of choice. The performances of DSCs sensitized with [Cu(11)(17)]^+^ and [Cu(11)(18)]^+^ are noticeably enhanced compared to cells with [Cu(11)(Me_2_bpy)]^+^. The improvement is associated with higher values of *J*_SC_ (3.79 mA cm^−2^ to 5.35 and 4.81 mA cm^−2^, Table 5, for DSCs measured on the same instrument) and this is also reflected in high external quantum efficiencies (EQEs) of 51% for [Cu(11)(17)]^+^ and 46% for [Cu(11)(18)]^+^ (EQE *λ*_max_ = 480 nm).^202^ On going from Me_2_bpy to the 4,4′-diphenyl derivative 19,^203^ a small increase in PCE is observed as a consequence of gains in both *J*_SC_ and *V*_OC_. The introduction of the peripheral halogen substituents in ancillary ligands 20–23 produces a significant rise in *J*_SC_, the optimum (*J*_SC_ = 6.92 mA cm^−2^) being for dye [Cu(11)(23)]^+^ with iodo substituents. The value of *V*_OC_ is also enhanced, leading to an overall *η* of 2.89%, which is 38.9% of the performance of a DSC with the reference dye N719. Despite the relative simplicity of the ancillary ligand, this remains one of the best performing copper-based dyes combined with an I_3_^−^/I^−^ redox shuttle reported to date, and this is possibly associated with enhanced electron transfer over the halogen of the aryl substituent from the reduced form of the redox couple.^204^ Independent studies of DSCs containing the dye [Cu(11)(22)]^+^ (Table 5) indicate that the use of MeCN in the dye bath (Fig. 9) rather than CH_2_Cl_2_ is detrimental to performance, probably due to the Lewis basicity and coordination tendency of MeCN. On going from ancillary ligand 22 to 25, the 6,6′-dimethyl substituents are replaced by 6,6′-diphenyl groups. By comparing data from DSC measurements made with the same instrumentation (Table 5), it is clear that the more sterically demanding phenyl substituents lead to slightly lower performances. More detrimental still is the introduction of phenyl substituents into the anchoring ligand, *i.e.* replacing the 6,6′-dimethyl groups in 11 by a 6,6′-diphenyl substituent pattern (11–Ph). Although the extended conjugation achieved with the phenyl groups leads to improved light absorption towards longer wavelengths, dyes with anchor 11–Ph rapidly bleach when exposed to the I_3_^−^/I^−^-containing electrolyte solution.^205^

We now move to dyes carrying peripheral methoxy groups. Interestingly, DSCs sensitized with [Cu(11)(23)]^+^ bearing the peripheral iodo groups^204^ outperform those using the related dye [Cu(11)(24)]^+^ carrying electron-releasing methoxy groups (Scheme 18 and Table 5).^203^ On going from 24 to 26 and 27, changes in the substitution pattern have a significant impact on DSC performance (Table 5), consistent with the electron-releasing nature of 4-MeO groups, and electron-withdrawing properties of 3- and 5-methoxy groups.^203^ Introduction of the asymmetrically substituted 28 and 29 ancillaries (Scheme 18) also give DSCs with rather high performances, and this is despite the fact that these ligands are based on a 6-Mebpy rather than a 6,6′-Me_2_bpy core.^206^

An investigation of a series of heteroleptic copper(i) dyes incorporating Schiff base ancillary ligands 30–34 concluded that the presence of the imine bond prevents efficient electron transfer across the dye.^207^ This can also be seen by comparing the performances of DSCs sensitized by dyes [Cu(11)(34)]^+^ (Schiff base) and [Cu(11)(35)]^+^ (no C=N unit). The presence of the C=N domain is detrimental to both *J*_SC_ and *V*_OC_ (Table 5). In [Cu(11)(35)]^+^ and [Cu(11)(36)]^+^, the electron-donating NR_2_ groups were expected to be beneficial in terms of stabilizing the hole remote from the TiO_2_ surface.^208^ Indeed, DSCs sensitized with these dyes performed well with respect to cells with the N719 reference dye (relative *η* = 33.4 and 36.0%, Table 5). However, as with the introduction of the imine functionality, inserting an alkyne unit between the phenylene and pyridine rings on going from ancillary ligand 35 to 37, or 36 to 38, resulted in significant decreases in *J*_SC_ values and in overall PCEs (Table 5).^208^

Ancillary ligands 39 and 40 (Scheme 18) represent first and second generation hole-transport dendrons. Going from [Cu(11)(39)]^+^ to [Cu(11)(40)]^+^ leads to enhanced light absorption towards lower energies, and although DSCs with [Cu(11)(40)]^+^ outperform those sensitized with [Cu(11)(39)]^+^, there was no gain in overall performance compared to the more structurally simple dyes listed in Table 5.^209^ With its sterically demanding and arene-rich second generation dendron, the dye [Cu(11)(40)]^+^ is likely to suffer from performance loss arising from aggregation. Hence, DSCs were also fabricated with cheno added to the dye. This had a significant impact on values of both *J*_SC_ and *V*_OC_ leading to a PCE of 2.23% compared to 1.50% without cheno. Interestingly, when acetone was used in the dye bath in place of CH_2_Cl_2_ (the step shown in Fig. 9), better DSC performances were observed for [Cu(11)(40)]^+^ although the enhancement gained by adding cheno was less noticeable.^212^ Like 39 and 40, ancillary ligands 41–44 (Scheme 19) were designed with electron-donating peripheral groups. They contain a phen metal-binding domain, and the design of 42–44 incorporates *n*-octyl chains to militate against electron recombination. For the SALSAC dye assembly (Fig. 9), the solvent used in the dye bath was MeCN. Compared to the DSCs with dyes containing bpy-based ancillary ligands (Me_2_bpy and 17–40), Table 5 reveals increased values of *J*_SC_ for cells sensitized with [Cu(11)(41)]^+^, [Cu(11)(42)]^+^, [Cu(11)(43)]^+^ or [Cu(11)(44)]^+^, leading to higher PCEs. A comparison of the cell parameters for the 5,6-substituted phen dyes indicates that there is little difference between the diphenylamine or carbazole hole-transporting domains. However, the introduction of the 4-(diphenylamino)phenylene hole-transporting units in the 4- and 7-positions of the phen unit is highly beneficial.^213^

**Fig. 10:**
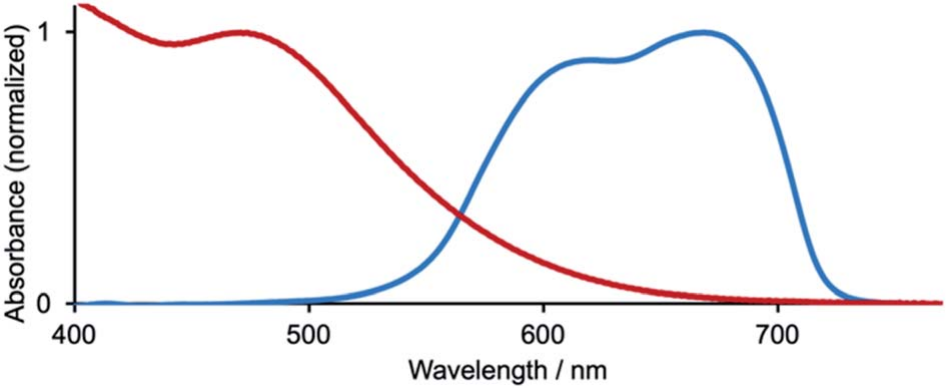
Solid-state absorption spectra of FTO/TiO_2_ electrodes with adsorbed dyes [Cu(11)(45)]^+^ (red) and SQ2 (blue). The high-energy tail arises from TiO_2_ absorption (spectra recorded by Frederik Malzner, University of Basel).

Compounds 45–48 (Scheme 20) return us to structurally simple ancillary ligands.^211^ A series of copper(i) dyes with asymmetrical ancillary ligands related to 45–48 in combination with the phosphonic acid anchoring ligand 11 was the basis for a theoretical study from Wei, Lu and coworkers in 2020. This work concluded that the use of these ancillaries coupled with appropriate functionalization (ligands 49–51) is effective in reducing the HOMO–LUMO energy gap and enhancing the light-harvesting of the dye.^214^ Indeed, DSCs sensitized with [Cu(11)(45)]^+^, [Cu(11)(46)]^+^, [Cu(11)(47)]^+^ and [Cu(11)(48)]^+^ gave some of the highest values of *V*_OC_ and *J*_SC_ (Table 5) observed for heteroleptic copper(i) dyes, and the PCE for [Cu(11)(45)]^+^ exceeded 3% for fully masked cells. Electrochemical impedance spectroscopy (EIS) was used to understand the striking DSC performances with [Cu(11)(45)]^+^ and [Cu(11)(48)]^+^. With [Cu(11)(45)]^+^, the device exhibited a high chemical capacitance and a low recombination resistance. However, the latter is offset by a low transport resistance, leading to a high *J*_SC_ and *V*_OC_. DSCs with [Cu(11)(48)]^+^ have the lowest transport resistance of this family of dyes.^211^ One of the major drawbacks of bis(diimine)copper(i) dyes is the spectral limitation of light absorption. The broad MLCT absorption of a simple [Cu(bpy)_2_]^+^ derivative typically has its maximum at *ca.* 460–480 nm, and although the absorption can be extended towards longer wavelengths by judicious functionalization, much of the region beyond 600 nm remains unharvested by the dye. A major improvement can be made by co-sensitization with a complementary organic dye. Suitable commercial dyes include SQ2 (Scheme 2) and Fig. 10 displays the solid-state absorption spectra of separate FTO/TiO_2_ electrodes functionalized with [Cu(11)(45)]^+^ and SQ2. After optimization of dye-bath conditions, the best performing masked DSC achieved values of *J*_SC_ = 9.56 mA cm^−2^, *V*_OC_ = 493 mV, *η* = 3.36% and relative *η* (relative to N719) = 44.5%. Upon ageing the DSC for a week, further improvement was observed with *J*_SC_ = 12.26 mA cm^−2^, *V*_OC_ = 515 mV, *η* = 4.51% and relative *η* of 65.6% with respect to N719.^215^ Since this report in 2017, the co-sensitization strategy has unfortunately not been exploited further, and we advocate this as a fruitful method of boosting the PCE of copper-based DSCs without the need for elaborate organic structure design.

Further application of the SALSAC approach is exemplified in the dipyrrin complexes^216^ which are discussed later with porphyinato derivatives.

### From homoleptic to heteroleptic complexes: the HETPHEN approach

**Scheme 21:**
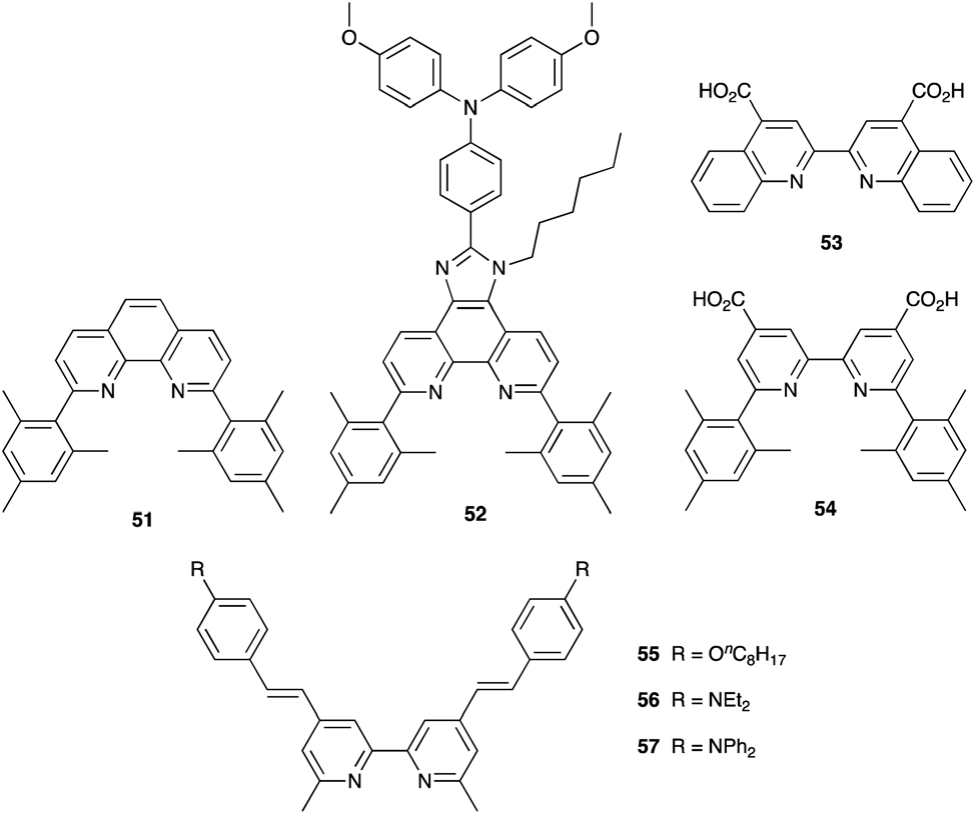
Anchoring and ancillary ligands used in the HETPHEN approach to copper(i) dyes.

**Fig. 11:**
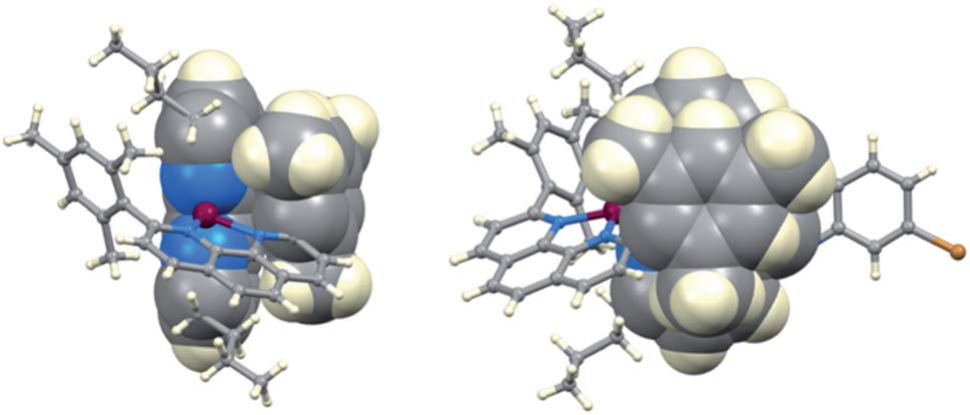
Two views of the structure of a [Cu(51)(phenazine)]^+^ derivative (CSD refcode RNAFAP) showing the π-stacking interaction between one mesityl group and the phen unit of the second ligand.

In contrast to our own approach of *in situ* assembly of a heteroleptic dye on TiO_2_, Odobel and coworkers have approached the problem of labile bis(diimine)copper(i) dyes by applying the HETPHEN strategy.^179,217,218^ The underlying principle of this approach is the use of a phen metal-binding domain bearing very sterically demanding mesityl substituents in the 2,9-positions.^219^ This motif can be in either the anchoring or ancillary ligand (ligands 51–54 in Scheme 21). In 2013, Sandroni *et al.* reported the synthesis of [Cu(51)(53)][PF_6_] and [Cu(52)(53)][PF_6_] using a stepwise method in which 51 or 52 was first reacted with [Cu(NCMe)_4_][PF_6_] to generate the intermediates [Cu(51)(NCMe)_*x*_]^+^ and [Cu(52)(NCMe)_*x*_]^+^. The steric hindrance of the mesityl substituents prevents the formation of [Cu(51)_2_]^+^ or [Cu(52)_2_]^+^. Reaction of the intermediates with *ca.* 0.9 equivalents of 53 leads to salts of [Cu(51)(53)]^+^ and [Cu(52)(53)]^+^.^179^ The structural, photophysical and electrochemical properties of related complexes had previously demonstrated the success of this synthetic strategy and also the stability of the heteroleptic species in solution. Fig. 11 shows the structure of a representative complex and highlights the π-stacking interaction between one mesityl group and the phen domain of the second ligand.^220^ The solid-state absorption spectra of [Cu(51)(53)]^+^ and [Cu(52)(53)]^+^ adsorbed on TiO_2_ exhibit broad MLCT bands with *λ*_max_ around 560 nm. DSCs incorporating [Cu(51)(53)]^+^ or [Cu(52)(53)]^+^ with an I_3_^−^/I^−^ redox mediator achieved PCEs of 0.49 and 0.22%, respectively, compared to 6.55% for a DSC with reference dye N719. Values of *J*_SC_ and *V*_OC_ were 1.61 mA cm^−2^ and 455 mV for [Cu(51)(53)]^+^, and 0.78 mA cm^−2^ and 445 mV for [Cu(52)(53)]^+^. Since the light-harvesting efficiency was found to be similar for the two dyes, the poorer performance of the DSC containing [Cu(52)(53)]^+^ was attributed to less efficient regeneration of the dye by the redox mediator. This initial study concluded that improved DSC performance should be possible by using electron-donating substituents on the ancillary ligand and employing a more rigid anchoring ligand.^179^

In 2014, Sandroni *et al.* confirmed the importance of the HETPHEN approach in achieving efficiently performing dyes. In the sensitizers [Cu(54)(Me_4_bpy)]^+^, [Cu(54)(55)]^+^, [Cu(54)(56)]^+^ and [Cu(54)(57)]^+^ (Me_4_bpy, see Scheme 6; 55–57, see Scheme 21), the mesityl groups are present in the anchoring ligand 54 (Scheme 21). Intramolecular π-stacking interactions are envisaged in these complexes, analogous to that found in the [Cu(51)(phenazine)]^+^ derivative shown in Fig. 11. Both [Cu(54)(56)]^+^ and [Cu(54)(57)]^+^ exhibit greater light-harvesting than [Cu(54)(Me_4_bpy)]^+^ and [Cu(54)(55)]^+^ as a consequence of an intense intraligand charge transfer (ILCT) band. The DSC performances of these dyes with and without the addition of cheno are summarized in Table 6; the redox shuttle was I_3_^−^/I^−^. The values of *η* compared with 7.36% for a reference DSC containing N719. The beneficial effects of cheno in preventing dye aggregation on the semiconductor surface are clear, with higher values of *J*_SC_ and *V*_OC_ in all cases. The PCEs of 4.42 and 4.66% for DSCs with [Cu(54)(56)]^+^/cheno and [Cu(54)(56)]^+^, respectively, remain the highest reported for heteroleptic copper(i) sensitizers. However, we note that no comments were made in the original work about the use of a DSC mask.^217^

**Table 6:** DSC parameters for cells containing heteroleptic copper(i) dyes assembled using the HETPHEN strategy in which the anchoring ligand is 54 (Scheme 21). DSCs were illuminated under a light intensity of 1000 W m^−2^ (ref. 217)

Dye/co-adsorbent	*J*_SC_/mA cm^−2^	*V*_OC_/mV	ff/%	*η*/%
[Cu(54)(Me_4_bpy)]^+^	2.20	475	72.80	0.76
[Cu(54)(Me_4_bpy)]^+^/cheno	3.76	525	74.64	1.47
[Cu(54)(55)]^+^	2.89	535	72.54	1.12
[Cu(54)(55)]^+^/cheno	4.99	565	72.39	2.04
[Cu(54)(56)]^+^	7.51	545	71.52	2.93
[Cu(54)(56)]^+^/cheno	10.86	605	70.97	4.66
[Cu(54)(57)]^+^	6.70	565	73.32	2.77
[Cu(54)(57)]^+^/cheno	10.13	625	69.76	4.42

The HETPHEN approach is extremely attractive for the preparation of robust copper(i) sensitizers. However, with the exception of work from Dragonetti *et al.* described later,^221^ there appears to have been little further progress in applications in DSCs since the initial work from the Odobel group. Additional investigations of the photophysical properties of sterically congested bis(diimine)copper(i) species prepared by the HETPHEN strategy have been reported.^222,223^

### Other heteroleptic copper(i) and copper(ii) dyes

**Scheme 22:**
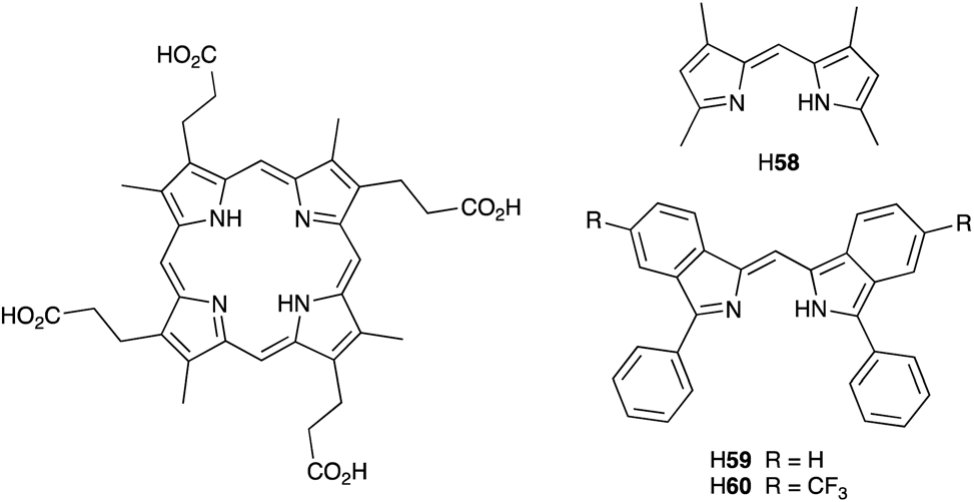
Structures of the porphyrin H_2_CPI, and three Hdipyrrin ligands.

In this section, we give an overview of copper photosensitizers other than bis(diimine)copper(i) complexes. In comparison to the large literature focused on porphyrinatozinc(ii) or phthalocyanatozinc(ii) dyes,^46,48,53–56^ those containing copper(ii) are rather sparse. Porphyrins and phthalocyanines exhibit intense absorption bands in both the high energy (Soret-band) and red/near-infrared (Q-band). A number of functionalized CuPc dyes in DSCs has been reported, with the degree of dye aggregation being a focus of attention.^224–228^ For dyes containing carboxylic acid-functionalized porphyrin domains, the nature of the carboxylic acid linker has a noteworthy impact on the performance of DSCs. In 2010, Grätzel and coworkers investigated the photoconversion efficiencies of DSCs sensitized with CPICu and CPIZn (H_2_CPI, Scheme 22) and using an I_3_^−^/I^−^ redox shuttle. Photoanodes were made with either a 3.3 μm thick layer of TiO_2_ or a double layer (7.5 + 5 μm) TiO_2_ architecture. Table 7 summarizes the performances of the DSCs, illustrating that CPICu-based DSCs outperform their zinc(ii) analogues, and that the thickness of the TiO_2_ layer significantly affects performance.^229^

**Table 7:** DSC parameters for masked cells sensitized with CPICu and CPIZn and with an electrolyte containing I_3_^−^/I^−^, 1,3-dimethylimidazolium iodide, and TBP and GCNS additives in MeCN/valeronitrile. DSCs were illuminated under a light intensity of 1000 W m^−2^ (ref. 229)

Dye	TiO_2_ layer or double layer/μm	*J*_SC_/mA cm^−2^	*V*_OC_/mV	ff/%	*η*/%
CPICu	3.3	5.7	655	70	2.6
CPICu	7.5 + 5	7.9	636	75	3.8
CPIZn	3.3	3.17	504	72	1.1
CPIZn	7.5 + 5	6.0	565	75	2.6

An interesting contribution from Leung and coworkers describes the fabrication of photoanodes comprising TiO_2_ sensitized with N719 and then covered with a 30 nm layer of CuPc. The design is predicated upon a cascade charge-transfer process involving the absorption of a near-infrared photon and creation of an exciton (electron–hole pair) which diffuses to the CuPc/N719 interface; electrons are transferred to the LUMO of N719 and then injected into the TiO_2_ conduction band. This innovative design leads to an increase in *J*_SC_ from 14.97 mA cm^−2^ (no CuPc) to 21.12 mA cm^−2^ (with CuPc) with negligible change in *V*_OC_. Overall, the PCE increases from 6.39 to 9.48%. The thickness of the CuPc layer was optimized to minimize quenching effects from the oxidized form of the I_3_^−^/I^−^ redox couple.^230^

**Scheme 23:**
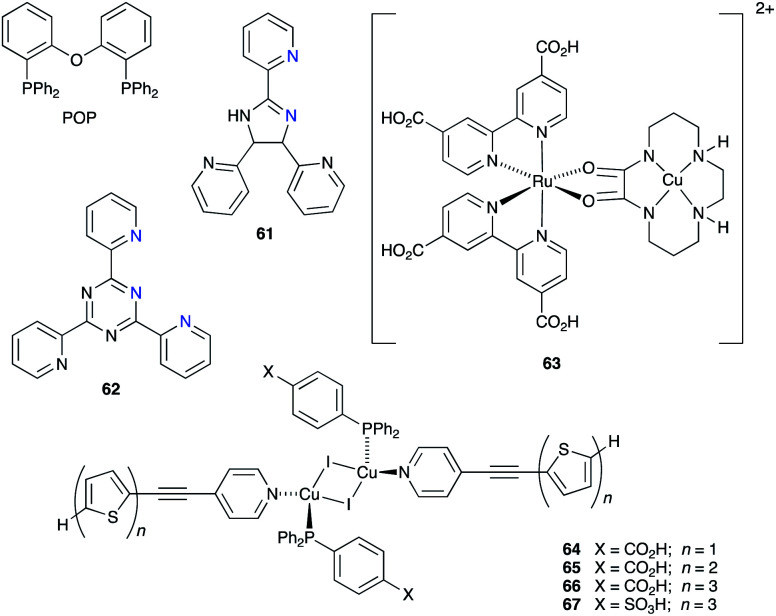
Structures of the ligands POP, 61 and 62, and the structures of the hetermetallic complex 63 and of the dinuclear copper(i) complexes from Jayapal *et al.*^235^ In 61 and 62, the N atoms shown in blue are the metal-binding sites.

In 2014, Robertson and coworkers screened a series of heteroleptic copper(i) complexes containing the dipyrrin ligands [58]^−^, [59]^−^ and [60]^−^ (Scheme 22) and anchoring ligand 1 (Fig. 1). The heteroleptic dyes were assembled using the SALSAC approach,^178^ with a mixture of [Cu(NCMe)_4_][BF_4_] and H58, H59 or H60 in the dye bath (using CH_2_Cl_2_ or acetone) rather than the isolated homoleptic copper(i) complex. An I_3_^−^/I^−^ redox mediator was used in the DSCs. Under a light intensity of 1000 W m^−2^, the DSCs achieved PCEs in the range 0.13 to 0.41%, the highest *J*_SC_ of 1.21 mA cm^−2^ leading to the highest value of *η*. Increasing the conjugation in the dipyrrin ligand on going from [58]^−^ to [59]^−^ and [60]^−^ led to an increase in DSC performance. However, better DSC performances were observed for a reference cell in which the TiO_2_-coated photoanode was immersed in a solution of [Cu(1)_2_][BF_4_] (*J*_SC_ = 2.33 mA cm^−2^, *V*_OC_ = 530 mV, *η* = 0.83%),^216^ and this should be compared with the results of Bessho *et al.* discussed earlier.^186^ Robertson and coworkers also investigated the use of [Cu(POP)(1)]^+^ as a photosensitizer in which POP is the wide-bite angle bisphosphane shown in Scheme 23. [Cu(P^P)(N^N)]^+^ complexes are more usually associated with their emissive properties and are of particular interest because of their ability to exhibit thermally-activated delayed fluorescence (TADF).^231^ DSCs containing [Cu(POP)(1)]^+^ and an I_3_^−^/I^−^ redox mediator gave very low PCEs (<0.1%) due to low values of both *V*_OC_ and *J*_SC_, even in the presence of cheno.^232^ Other [Cu(P^P)(N^N)]^+^ complexes that have been trialled in DSCs include [Cu(PPh_3_)_2_(61)][PF_6_] and [Cu(PPh_3_)_2_(62)][PF_6_]. The crystal structures of the latter show a distorted tetrahedral Cu(i) centre in [Cu(PPh_3_)_2_(61)]^+^, while in [Cu(PPh_3_)_2_(62)]^+^, the Cu(i) ion is 5-coordinate although one Cu–N bond is longer (3.020(2) Å) than is typical. DSCs were made using N719 alone as the dye, and with TiO_2_ photoanodes co-sensitized with N719 and [Cu(PPh_3_)_2_(61)]^+^, or N719 and [Cu(PPh_3_)_2_(62)]^+^. Given that the amount of N719 in the dye baths for the latter was half that in the pristine N719-based cells, it is noteworthy that the DSC containing N719 and [Cu(PPh_3_)_2_(62)]^+^ reached a relative PCE of 73.1% with respect to the pristine N719-based cell. However, low fill-factors contributed to low PCEs for all devices.^233^ In contrast to this co-sensitization approach, Robertson and coworkers designed a dimetallic complex 63 (Scheme 23) in which the Ru(ii) centre is in an environment similar to that in N3 or N719, and the Cu(ii) centre is in a cyclam cavity. The absorption range and molar extinction coefficients of the dye were enhanced relative to those of N719. However, combined with an I_3_^−^/I^−^ redox mediator, the best PCE that was obtained under light irradiation of 1000 W m^−2^ was 2.55% relative to 6.4% for an N719 reference DSC. This was attributed to an energy mismatch between the TiO_2_ conduction band and the LUMO of the dye combined with the instability of the dye in the electrolyte solution.^234^

The neutral dinuclear copper(i) complexes 64–67 (Scheme 23) were designed with thienyl domains although these are not associated with the anchoring units. Solid-state absorption spectra of the compounds adsorbed on TiO_2_ exhibit broad bands which are most red-shifted for 66 and 67 which contain the terthienyl substituents. However, the absorption maximum for each of the four compounds is <400 nm. Theoretical studies revealed that the LUMO of each compound lies above the TiO_2_ conduction band, suggesting that the 64–67 are suited as DSC sensitizers. DSCs fabricated with these dyes and an I_3_^−^/I^−^ redox mediator achieved PCEs in the range 0.15–1.56%, with the highest value being for dye 67. Although the performances were generally low, the trends were consistent with a sulfonic acid anchoring unit being more beneficial than a carboxylic acid.^235^

### All-copper DSCs

We have already detailed how the use of Cu^2+^/Cu^+^ redox mediators with organic dyes leads to impressive values of *V*_OC_ in excess of 1000 mV. The natural progression towards DSCs with sustainable components is the combination of copper-containing dyes and copper-based redox shuttles. Despite yielding some promising results, this approach has, as yet, received little attention.

Dragonetti *et al.* applied the HETPHEN approach to prepare [Cu(9)(51)][BF_4_] (for 9 and 51, see Schemes 15 and 21). DSCs were made using [Cu(9)(51)]^+^ as the dye and I_3_^−^/I^−^, [CuCl(Me_2_phen)_2_]^+^/[Cu(Me_2_phen)_2_]^+^ or [Cu(Buphen)_2_]^2+^/[Cu(Buphen)_2_]^+^ (Buphen = 2-*n*-butyl-1,10-phenanthroline) as redox mediators. DSC performances were reported for unmasked cells (Table 8), with a comment that performances decreased by about 25–30% when a mask was applied. The high value of *V*_OC_ = 750 mV obtained with the [CuCl(Me_2_phen)_2_]^+^/[Cu(Me_2_phen)_2_]^+^ redox couple was offset by a decreased value of *J*_SC_ (Table 8), and the fill factors with the copper-based redox shuttles were also low.^236^ Dragonetti *et al.* developed their work to encompass dyes [Cu(9)(L_ancillary_)]^+^ in which the ancillary ligands were structurally similar to 52 (Scheme 21). Combined with [CuCl(Me_2_phen)_2_]^+^/[Cu(Me_2_phen)_2_]^+^ or [Cu(Buphen)_2_]^2+^/[Cu(Buphen)_2_]^+^ redox couples, masked DSCs achieved *V*_OC_ and *J*_SC_ values of up to 694 mV and 3.8 mA cm^−2^, respectively, and PCEs of up to 1.4% (16% with respect to an N719 reference cell set at 100%).^221^ Optimization of the composition of the electrolyte solution is critical, and Colombo *et al.* have demonstrated the key roles of TBP and LiTFSI. DSCs sensitized with [Cu(9)(51)]^+^ as the dye were tested with various electrolyte compositions, and the best performing masked device which contained both TBP and LiTFSI realized values of *J*_SC_, *V*_OC_ and *η* of 5.77 mA cm^−2^, 622 mV and 2.51%, respectively.^237^

**Table 8:** DSC parameters for the all-copper devices (unmasked) compared with N719 from the work of Dragonetti *et al.*^236^ See footnotes for electrolyte components. Light illumination of 1000 W m^−2^

Dye	Redox mediator	*J*_SC_/mA cm^−2^	*V*_OC_/mV	ff/%	*η*/%
N719	I_3_^−^/I^−^a^,^^b^	15.4	800	71	8.9
[Cu(9)(51)]^+^	I_3_^−^/I^−^a^,^^b^	9.0	610	63	3.5
[Cu(9)(51)]^+^	I_3_^−^/I^−^a^,^^c^	8.2	670	65	3.6
[Cu(9)(51)]^+^	[CuCl(Me_2_phen)_2_]^+^/[Cu(Me_2_phen)_2_]^+^^d^	4.7	750	36	1.3
[Cu(9)(51)]^+^	[Cu(Buphen)_2_]^2+^/[Cu(Buphen)_2_]^+^^d^	6.3	610	53	2.0

a 0.28 M TBP in valeronitrile/MeCN.

b 0.65 M *N*-methyl-*N*-butylimidazolium iodide, 0.025 M LiI, 0.04 M I_2_; for N719 DSC, GNCS was added.

c 0.26 M *N*-methyl-*N*-butylimidazolium iodide, 0.01 M LiI, 0.017 M I_2_.

d 0.17 M Cu(i): 0.017 M Cu(ii) + 0.1 M LiTFSI in MeCN.

**Scheme 24:**
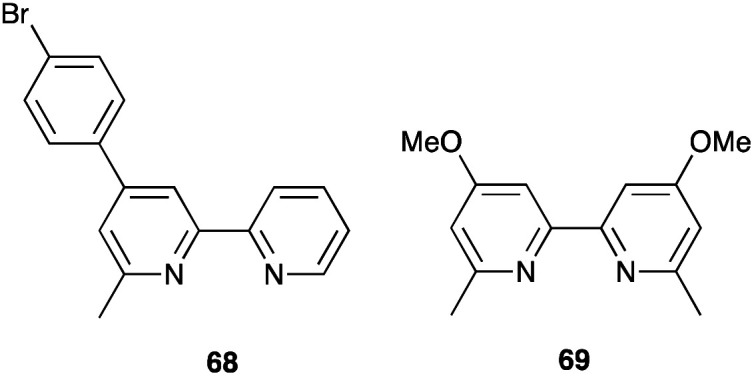
Structures of ancillary ligand 68, and of ligand 69 used in a Cu^2+^/Cu^+^ redox shuttle.

Around the same time as the first results from Dragonetti *et al.* were published,^236^ we also demonstrated the successful combination of copper(i) dyes and Cu^2+^/Cu^+^ redox mediators.^238^ The SALSAC approach was used to functionalize photoanodes with the dyes [Cu(11)(Me_2_bpy)]^+^, [Cu(11)(Me_2_phen)]^+^, [Cu(11)(22)]^+^, [Cu(11)(68)]^+^ and [Cu(11)(69)]^+^ (11, Scheme 15; 22, Scheme 18; 68 and 69, Scheme 24) and these were combined with the redox mediators [Cu(22)_2_]^2+^/[Cu(22)_2_]^+^, [Cu(68)_2_]^2+^/[Cu(68)_2_]^+^, [Cu(Me_2_bpy)_2_]^2+^/[Cu(Me_2_bpy)_2_]^+^, [Cu(Me_2_phen)_2_]^2+^/[Cu(Me_2_phen)_2_]^+^ and [Cu(69)_2_]^2+^/[Cu(69)_2_]^+^ in masked DSCs under light illumination of 1000 W m^−2^. In comparison to a value of *V*_OC_ = 614 mV for an N719 (with I_3_^−^/I^−^) reference DSC, the all-copper DSCs achieved values of *V*_OC_ in the range 558–796 mV. Values of *J*_SC_ varied from 1.10 to 4.01 mA cm^−2^ compared to 12.54 mA cm^−2^ for N719. The most promising redox mediator was found to be [Cu(69)_2_]^2+^/[Cu(69)_2_]^+^ which contains peripheral methoxy-substituents. Data for the best performing dye/redox mediator combinations are given in Table 9. The highest PCE of 2.06% corresponded to a relative efficiency of 38.1% with respect to the N719 reference DSC. An established problem with Cu^2+^/Cu^+^ redox mediators is the high diffusion resistance (*R*_d_) and electrochemical impedance spectroscopic data confirmed this for all the DSCs; values of *R*_d_ were in the range 115 to 1005 Ω. This leads to low electron transport in the electrolyte with consequent non-optimal regeneration of the oxidized sensitizer and low *J*_SC_ values. In keeping with the results of Colombo *et al.*,^237^ we found TBP to be a beneficial component of the electrolyte.^238^ On the other hand, given the susceptibility of Cu(ii) to coordination by TBP and other Lewis bases (see earlier discussion), the role TBP with Cu^2+^/Cu^+^ couples remains to be further explored in all-copper DSCs.

**Table 9:** DSC (masked cells) parameters for the best combinations of bis(diimine)copper(i) dyes and Cu^2+^/Cu^+^ redox couples compared with N719 from the work of Karpacheva *et al.*^238^ Light illumination of 1000 W m^−2^

Dye	Redox mediator^a^	*J*_SC_/mA cm^−2^	*V*_OC_/mV	ff/%	*η*/%
N719	I_3_^−^/I^−^	12.54	614	70	5.40
[Cu(11)(69)]^+^	[Cu(69)_2_]^2+^/[Cu(69)_2_]^+^	4.01	684	75	2.06
[Cu(11)(Me_2_bpy)]^+^	[Cu(69)_2_]^2+^/[Cu(69)_2_]^+^	3.85	686	76	2.00
[Cu(11)(Me_2_phen)]^+^	[Cu(Me_2_phen)_2_]^2+^/[Cu(Me_2_phen)_2_]^+^	2.98	804	74	1.76
[Cu(11)(Me_2_bpy)]^+^	[Cu(Me_2_phen)_2_]^2+^/[Cu(Me_2_phen)_2_]^+^	2.80	796	73	1.63
[Cu(11)(68)]^+^	[Cu(Me_2_phen)_2_]^2+^/[Cu(Me_2_phen)_2_]^+^	3.09	812	72	1.82

a Electrolyte composition: [CuL_2_][PF_6_]_2_/[CuL_2_][PF_6_] in a molar 1 : 5 ratio, 0.5 M TBP, 0.1 M LiPF_6_ in MeCN.

## The holy grail: iron in DSCs

### The promise of iron(ii) sensitizers

In 2004, in reviewing metal complexes as sensitizers, Polo *et al.* stated: “As iron is a common and cheap metal, it can provide a very economical alternative to ruthenium in sensitizing complexes”.^239^ However, progress in this field has been slow and the reasons for this are effectively summarized by Wenger who, in 2019, posed the question: “Is iron the new ruthenium?”.^240^ Of metal-based photosensitizers, some of the highest photoconversion efficiencies are realized using ruthenium(ii) compounds. However, as we have already noted, the very low crustal abundance of ruthenium (*ca.* 0.001 ppm)^27^ makes its use on a commercial scale non-sustainable. Like Ru(ii), Fe(ii) has a d^6^ electronic configuration. However, the photophysical behaviour of iron(ii) polypyridine complexes does not mirror that of their ruthenium(ii) counterparts. While polypyridyl Ru(ii) complexes possess long-lived MLCT excited states in a characteristic range of 100–1000 ns, the MLCT excited states in polypyridyl Fe(ii) complexes suffer extremely fast deactivation.^240^ Although this militates against their use as photosensitizers, early investigations by Ferrere *et al.*^241,242^ confirmed that functioning DSCs were possible using simple dyes such as [Fe(4,4′-(HO_2_C)_2_bpy)_2_(CN)_2_] (a close analogue of N3, Scheme 3) in the presence of the co-adsorbent cheno. We have previously reviewed these and related early results in detail.^175^ More recently, Jakubikova and coworkers published the results of DFT and time-dependent DFT (TD-DFT) studies on the ground and excited state properties of [Fe(4,4′-(HO_2_C)_2_bpy)_2_(CN)_2_], [Fe(4,4′-(HO_2_C)_2_bpy)(CN)_4_]^2−^ and [Fe(bpy)(CN)_4_]^2−^. Each complex exhibits two absorption bands in the visible region, both with dominant MLCT character, and it was concluded that all three complexes may undergo interfacial electron transfer (IET) on a time-scale that is competitive with the ultrafast intersystem crossing of the initially excited ^1^MLCT states into the low-lying metal-centred (MC) states. TiO_2_-anchored [Fe(4,4′-(HO_2_C)_2_bpy)_2_(CN)_2_] exhibits band-selective sensitization because of poor energy matching of the lowest energy excited states with the conduction band of TiO_2_. These calculational results^243^ are consistent with the earlier experimental observations of Ferrere *et al.*^241,242^ A complementary theoretical study looked at the effects of different anchoring domains in [Fe(4,4′-X_2_bpy)_2_(CN)_2_] in which X = carboxylic acid, phosphonic acid, hydroxamate, catechol and acetylacetonate. The results suggest that hydroxamate anchors could be particularly beneficial,^244^ but to the best of our knowledge, this has not been confirmed in practice. Using a computational approach, Tsaturyan *et al.* investigated the effects of having one, two, three or four CO_2_H anchors in the dye [Fe(qtpy)(NCS)_2_], the anchors being introduced into the 4-, 4′-, 4′′- and 4′′′-positions of 2,2′:6′,2′′:6′′,2′′′-quaterpyridine (qtpy). In terms of the absorption spectrum and energies of the frontier MOs of the complex, it was concluded that the optimal number of anchors is two.^245^ An overview of computational work focused on polypyridyl iron(ii) complexes as sensitizers published in 2015 gives an excellent insight into the ground rules for ligand design, in particular in addressing the relative rates of intersystem crossing (ISC) and IET processes, the aim being to ensure that IET becomes more competitive with ISC.^246^

A further investigation from Jakubikova and coworkers is relevant to the move from polypyridyl to N-heterocyclic carbene (NHC) complexes of iron(ii) as dyes in DSCs (see below). Starting with [Fe(tpy)_2_]^2+^ and [Fe(dcpp)_2_]^2+^ (dcpp = 2,6-bis(2-carboxypyridyl)pyridine), the field strength of the terdentate ligands was systematically altered by replacing the central pyridine ring with 5-membered (NHC, pyrrole, furan) or 6-membered (aryl, thiazine-1,1-dioxide, 4-pyrone) units. For applications of these complexes as dyes, several design principles are advocated: (i) the presence of Fe–C bonds (*i.e.* NHC ligands are favoured), (ii) as ideal an octahedral environment around Fe(ii) as possible (*i.e.* 6- rather than 5-membered ring as the central unit in the terdentate ligand), and (iii) short Fe–X_ligand_ bond lengths.^247^ Ashley and Jakubikova have also developed a computational approach to predicting the redox behaviour of polypyridyliron(ii) complexes which should assist in the design of Fe(ii)-based dyes for DSCs.^248^

**Fig. 12:**
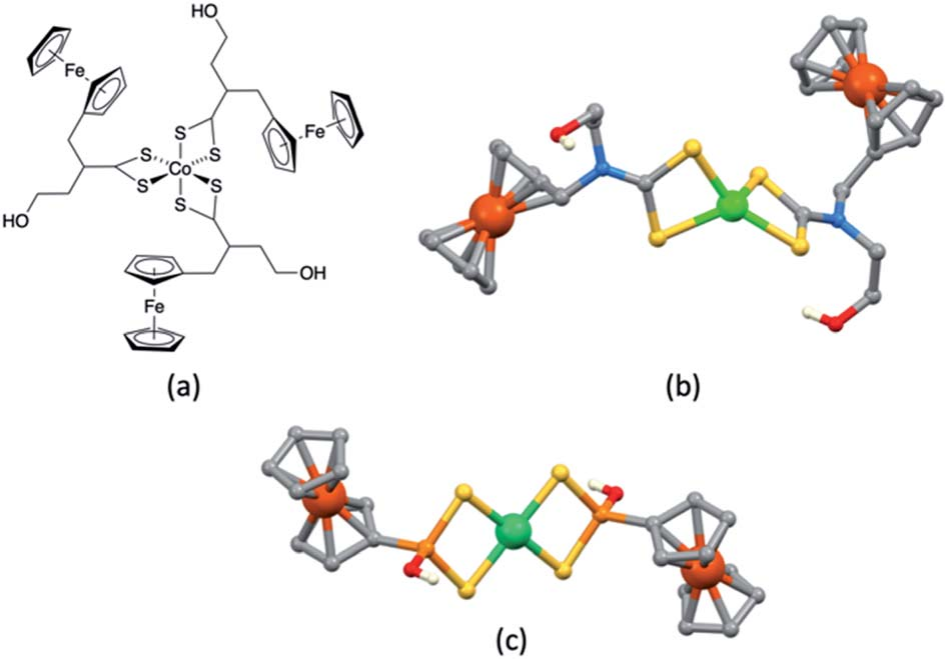
Examples of ferrocenyl dithiocarbamate metal complexes used as sensitizers, (a) a cobalt(iii) complex, and (b) a zinc(ii) complex (CSD refcode EJAYUL). Both have hydroxyl anchoring groups. (c) Example of a ferrocenyl dithiophosphonate complex of nickel(ii) with P–OH anchors (refcode XORKAT).

Dyes containing ferrocenyl units also received attention in the period up to 2013, although performances in DSCs were typically poor.^175^ Several later investigations of ferrocenyl derivatives have focused on ferrocenyl dithiocarbamate metal complexes.^249–253^ A masked DSC combining an I_3_^−^/I^−^ redox mediator with the Co/Fe dye shown in Fig. 12a achieved values of *J*_SC_ = 7.23 ± 0.03 mA cm^−2^, *V*_OC_ = 620 ± 10 mV and *η* = 3.25 ± 0.04% under light intensity of 1000 W m^−2^.^252^ Yadav *et al.* have also reported the enhancement of N719-based DSCs by co-sensitization with the ferrocenyl dithiocarbamate zinc(ii) complex shown in Fig. 12b. An average value for three devices of *η* = 7.10 ± 0.02% was obtained which compared to *η* = 5.76 ± 0.04% for N719 alone with an I_3_^−^/I^−^ redox shuttle.^251^ The group of van Zyl has reported a series of ferrocenyl-decorated dithiophosphonate complexes of Ni(ii), Zn(ii) and Cd(ii) which have been tested as single dyes and as co-sensitizers with N719 in DSCs. The anchoring domains are P–OH units (Fig. 12c). Using a commercial I_3_^−^/I^−^-based electrolyte, the performances of unmasked DSCs reached PCEs of 2.43, 3.58 and 3.12% (averages of three cells) for the Ni(ii), Zn(ii) and Cd(ii)-centred dyes compared to 7.19% for N719. Co-sensitization with N719 led to slightly enhanced performances with respect to N719 alone for the Zn(ii) and Cd(ii)-based dyes.^254^

Several examples of derivatives of 1,1′-bis(diphenyl)phosphanoferrocene (dppf) have been trialled as sensitizers in DSCs.^255,256^ The dimetallic complexes [Ni(dppf)(S_2_C=C(H)NO_2_)] and [Ni(dppf)(S_2_C=C(COMe)_2_)] absorb in the visible with maxima *ca.* 430 nm when adsorbed on TiO_2_. The dyes were combined with an I_3_^−^/I^−^ redox mediator in unmasked DSCs which were illuminated under a light intensity of 1000 W m^−2^. Compared to values of *J*_SC_, *V*_OC_ and *η* = 13.22 mA cm^−2^, 730 mV and 6.91% for a reference N719 DSC, the ferrocenyl dyes performed with values of *J*_SC_ = 5.87 and 7.96 mA cm^−2^, *V*_OC_ = 644 and 654 mV and *η* = 2.32 and 3.21%. However, the authors conclude that a change in the anchoring domain from the nitro and diacetyl present in [Ni(dppf)(S_2_C=C(H)NO_2_)] and [Ni(dppf)(S_2_C=C(CMeO)_2_)], respectively, could be beneficial.^256^

In a series of papers, Özacar and coworkers reported the use of complexes of iron(ii) incorporating tannin or quercetin ligands as sensitizers with either TiO_2_ or ZnO coated photoanodes.^257–260^

### The N-heterocyclic carbene era arrives

The bottleneck inhibiting the use of polypyridyl iron(ii) complexes as dyes in DSCs is their fast deactivation from an MLCT to lower lying MC states.^240^ This leads to inefficient electron injection into the semiconductor and, therefore, low *J*_SC_ values. A review from Visbal and Gimeno in 2014, looked at the progress made in the field of luminescent transition metal complexes with HNC ligands, and included applications in DSCs. However, at this stage, the focus was still on ruthenium(ii) complexes.^261^ Theoretical investigations in the period 2014–2015 highlighted the advantages of using cyclometallating or N-heterocyclic carbene ligands (which are strongly σ-donating) with iron(ii) in place of polypyridyl metal-binding domains.^247,262^

**Scheme 25:**
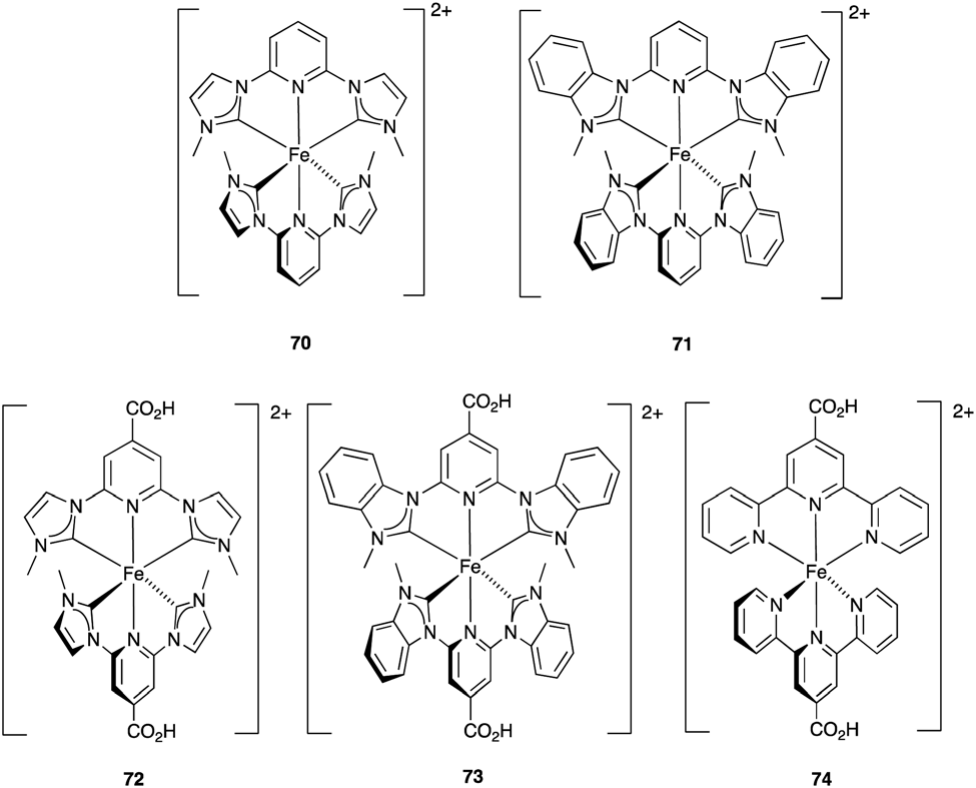
Structures of the homoleptic NHC iron(ii) complexes 70–73 and of [Fe(4′-HO_2_Ctpy)_2_]^2+^ (74).

**Fig. 13:**
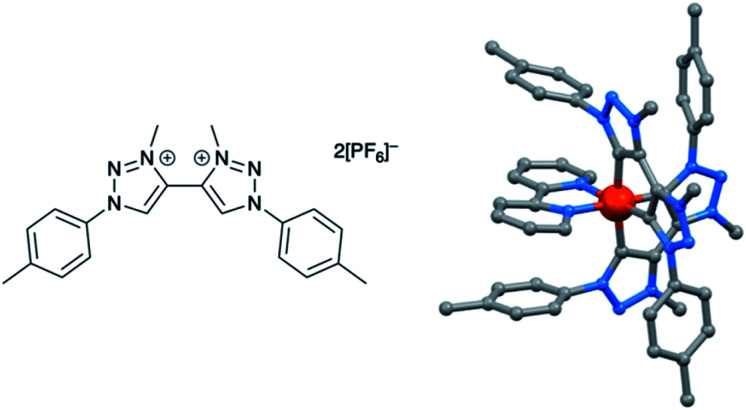
The 1,2,3-triazol-5-ylidene-containing compound [H_2_btz][PF_6_]_2_ and the structure of [Fe(bpy)(btz)_2_]^2+^ (CSD refcode NOVVAX).

In practice, 2013 saw the preparation of the first homoleptic NHC complex of iron(ii) (70, Scheme 25) which exhibited an extended ^3^MLCT lifetime (9 ps). This pivotal work from Wärnmark and coworkers^263^ increased the excited-state lifetime by a factor of 100 in comparison to previously reported polypyridyl iron(ii) complexes. In addition, transient absorption measurements showed no significant population of ^5^MC states, consistent with deactivation pathways in NHC iron(ii) compounds such as 70 being fundamentally different from those in polypyridyl iron(ii) complexes. The long excited-state lifetime is a consequence of a significant destabilization of both ^3^MC and ^5^MC states with respect to those in polypyridyl iron(ii) complexes.^264^ Replacing the NHC ligands in 70 by the corresponding benzimidazolylidene-based ligands to give complex 71 (Scheme 25) increased the excited ^3^MLCT state lifetime to 16.4 ps.^265^ Wärnmark, Sundström and coworkers also demonstrated that use of strongly σ-donating 1,2,3-triazol-5-ylidene domains in the heteroleptic complex [Fe(bpy)(btz)_2_]^2+^ (Fig. 13) led to an excited ^3^MLCT state lifetime of 13 ps.^266^ Another highly relevant publication around this time came from Kühn and coworkers^267^ who demonstrated that the electronic structure of NHC iron(ii) complexes is strongly influenced by the number of NHC donors. Cyclic voltammetric data confirmed a linear correlation between the oxidation potentials of the iron(ii) complexes and the number of NHC donors.

All of the investigations of NHC iron(ii) compounds described above came closely on the heel of one another, and heralded the arrival of NHC iron(ii) dyes.^268^ In 2015 both Gros and coworkers^269^ and Wärnmark and coworkers^270^ reported the synthesis of the carboxylic acid anchor-containing complex 72 (Scheme 25). The introduction of the CO_2_H groups on going from 70 to 72 leads to an increase in the lifetime of the ^3^MLCT excited state from 9 to 18 ps (in MeCN solution).^270^ A similar elongation of the ^3^MLCT lifetime (from 16.4 to 26 ps) is seen on introducing CO_2_H functionalities into 71 to give 73 (Scheme 25).^265^ When complex 72 was anchored on an Al_2_O_3_ surface, the lifetime was further extended to 37 ps. The latter was (in 2015), the longest excited-state lifetime reported for any mononuclear iron complex. This detailed investigation also provided evidence for efficient photo-induced electron injection from the ^3^MLCT state into the conduction band of TiO_2_ with a 92% yield of conversion to photoelectrons in the conduction band. Wärnmark and coworkers noted that a proportion of the injected electrons underwent fast electron–dye recombination.^270^ We return to this problem later. As this latter work was in progress,^270^ Gros and coworkers reported the first application of complex 72 as a sensitizer in DSCs, and also compared the photophysical properties of 72 with the tpy derivative 74 (Scheme 13). Notably, while the solution absorption spectrum of 74 exhibits one MLCT band in the visible region (*λ*_max_ = 569 nm), that of compound 72 shows two bands (*λ*_max_ = 394 and 520 nm), assigned to MLCT involving the carbene and pyridine rings, respectively. This is a general feature of this family of dyes and contributes to better light harvesting in the visible region. Under illumination of 1000 W m^−2^, DSCs containing 72 and the co-adsorbent cheno (Scheme 1) with an I_3_^−^/I^−^ redox mediator achieved values of *J*_SC_ = 0.41 mA cm^−2^, *V*_OC_ = 457 mV and *η* = 0.13% (entry 1, Table 10). These values compared to 0.016 mA cm^−2^, 250 mV and 0.01% for DSCs with dye 74. For comparison, a reference DSC with N719 gave a PCE of 6.1%. Data were reproducible for three DSCs per dye.^269^ Although the performances were low, these results were encouraging, and provided motivation for further optimization of DSC components.

**Table 10:** DSC parameters for cells sensitized with homoleptic NHC iron(ii) dye 72 and heteroleptic dyes 75–83. Light illumination = 1000 W m^−2^. For dye 72 table entry 3, and dyes 82 and 83, the parameters are for the best performing DSC and the values in parentheses are average data from four cells

Dye	Co-adsorbent	Electrolyte	Cell fabrication details	*J*_SC_/mA cm^−2^	*V*_OC_/mV	ff/%	*η*/%	Ref.
72	cheno	AN50^a^	Sealed DSC; Pt/FTO counter-electrode	0.41	457	68	0.13	278
72	cheno	b	Sealed DSC; masked; Pt/FTO counter-electrode	2.31	339	65	0.51	274
72	cheno	b	Sealed DSC; masked; dense TiO_2_ underlayer on photoanode; Pt/FTO counter-electrode	2.09 (1.90 ± 0.2)	466 (460 ± 20)	75.2 (74 ± 10)	0.73 (0.63 ± 0.07)	271
72	cheno	c	Sealed DSC; masked; Pt/FTO counter-electrode	3.82 ± 0.14	285 ± 9	60 ± 1	0.65 ± 0.02	275
72	cheno	d	Open-configuration DSC; blocking underlayer on photoanode; Pt/FTO counter-electrode	3.30	440	63	0.92	277
75	cheno	AN50^a^	Sealed DSC; Pt/FTO counter-electrode	0.33	400	73	0.10	278
75	cheno	d	Open-configuration DSC; masked; blocking underlayer on photoanode; PEDOT/FTO counter-electrode	3.95	490	61	1.18 ± 0.10	279
75	cheno	e	Open-configuration DSC; masked; blocking underlayer on photoanode; PEDOT/FTO counter-electrode	4.44	450	64	1.27 ± 0.12	279
75	cheno	d	Open-configuration DSC; masked; blocking underlayer on photoanode; Pt/FTO counter-electrode	4.26	510	59	1.29 ± 0.09	279
75	cheno	e	Open-configuration DSC; masked; blocking underlayer on photoanode; Pt/FTO counter-electrode	4.98	470	62	1.44 ± 0.07	279
76	cheno	AN50^a^	Sealed DSC; Pt/FTO counter-electrode	0.36	440	73	0.11	278
77	cheno	AN50^a^	Sealed DSC; Pt/FTO counter-electrode	0.36	390	71	0.10	278
78		f	Sealed DSC; masked; Pt/FTO counter-electrode	3.68	417	62	0.94	280
78	cheno	f	Sealed DSC; masked; Pt/FTO counter-electrode	3.00	397	60	0.71	280
79		d	Open-configuration DSC; masked; blocking underlayer on photoanode; PEDOT/FTO counter-electrode	2.69	460	63	0.78 ± 0.08	279
79		e	Open-configuration DSC; masked; blocking underlayer on photoanode; PEDOT/FTO counter-electrode	2.90	450	62	0.81 ± 0.10	279
80		d	Open-configuration DSC; masked; blocking underlayer on photoanode; PEDOT/FTO counter-electrode	3.55	440	60	0.94 ± 0.11	279
80		e	Open-configuration DSC; masked; blocking underlayer on photoanode; PEDOT/FTO counter-electrode	3.89	430	57	0.95 ± 0.09	279
81		e	Open-configuration DSC; blocking underlayer on photoanode; PEDOT/FTO counter-electrode	6.80 ± 0.17	470 ± 20	57 ± 1	1.83 ± 0.10	281
82	cheno	b	Sealed DSC; masked; dense TiO_2_ underlayer on photoanode; Pt/FTO counter-electrode	3.52 (3.3 ± 0.2)	512 (500 ± 10)	72.4 (71.4 ± 8)	1.31 (1.2 ± 0.1)	271
83	cheno	b	Sealed DSC; masked; dense TiO_2_ underlayer on photoanode; Pt/FTO counter-electrode	3.23 (2.7 ± 0.4)	416 (400 ± 20)	69.4 (69 ± 10)	0.93 (0.8 ± 0.2)	271

a Commercial electrolyte: I_2_ (0.05 M), LiI (0.1 M), DMPII and TBP (0.5 M) in MeCN.^272,273^

b LiI (0.1 M), I_2_ (0.05 M) and DMPII (0.60 M) in MPN.

c LiI (0.18 M), I_2_ (0.10 M) and DMII (0.60 M) in MPN.

d LiI (0.1 M), I_2_ (0.1 M), PMII (0.60 M), MgI_2_ (0.1 M) and GNCS (0.1 M) in MeCN.

e LiI (0.1 M), I_2_ (0.1 M), PMII (0.60 M), MgI_2_ (0.1 M), Bu_4_NI (0.1 M) and GNCS (0.1 M) in MeCN.

f LiI (0.18 M), I_2_ (0.05 M) and DMPII (0.60 M) in MPN.

### Improving the performances of DSCs with NHC iron(ii) dyes: electrolyte tuning

**Scheme 26:**
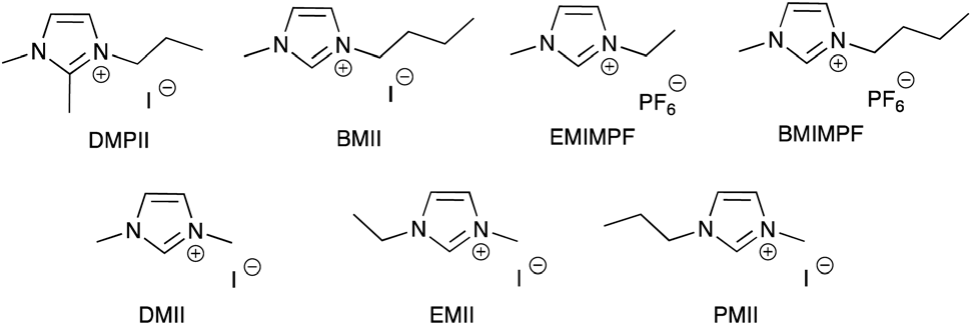
Structures of some ionic liquids (ILs) used in DSC electrolytes.

Starting with dye 72, several strategies have been followed to improve the performances of NHC iron(ii)-based DSCs. The first is optimization of the electrolyte composition. Until recently,^271^ NHC iron(ii)-based DSCs have only incorporated the I_3_^−^/I^−^ redox mediator, and a commercial electrolyte (AN50) was used in the initial report from Gros.^269^ AN50 comprises I_2_ (0.05 M), LiI (0.1 M), the ionic liquid 1,2-dimethyl-3-propylimidazolium iodide (DMPII, Scheme 26) and TBP (0.5 M) in MeCN.^272,273^ In 2018, we performed a detailed investigation of the effects of solvent (MeCN or MPN), different ionic liquids (DMPII, BMII, EMIMPF and BMIMPF, Scheme 26) and the presence and varying concentrations of the additives TBP and 1-methylbenzimidazole (MBI) on the performance of masked DSCs containing the dye 72, the co-adsorbent cheno and an I_3_^−^/I^−^ redox mediator. Use of MPN rather than MeCN as electrolyte solvent resulted in higher values of *J*_SC_ with little impact in *V*_OC_. The electrolyte composition that gave the best performing DSCs comprised LiI (0.1 M), I_2_ (0.05 M), DMPII (0.6 M) with no or 0.01 M MBI. Using multiple DSCs to confirm reproducibility, we realized values of *J*_SC_ in the range 2.31 to 2.78 mA cm^−2^, *V*_OC_ in the range 292 to 374 mV and values of *η* in the range of 0.47 to 0.57%. These PCEs represented 7.8–9.3% of the PCE recorded for N719 reference cells. Entry 2 in Table 10 shows one of the best-performing cells.^274^ Recently, Lindh *et al.* reported the performances of DSCs with 72 that used the same electrolyte composition as in our study^274^*i.e.* LiI (0.1 M), I_2_ (0.05 M), DMPII (0.6 M) in MPN. Although cheno was added as a co-adsorbent, Lindh *et al.* observed competitive anchoring of cheno and 72 on the TiO_2_ surface. They therefore used a sequential treatment of the surface with 72 followed by cheno, rather than a single dye bath with both adsorbents. This, as well as the use of a dense TiO_2_ underlayer (deposited by spray pyrolysis of a [Ti(O^i^Pr)_2_(acac)_2_] solution) leads to improved performance as is appreciated by comparing entries 2 and 3 in Table 10. The increase in *V*_OC_ is particularly marked. Reproducibility of the DSC parameters was checked by measuring four cells, and ranges of values are given in Table 10.^271^

Further tuning of the electrolyte composition^275^ focused on optimizing the concentration of I_2_ coupled with different ionic liquids (ILs). For dye 72, values of *J*_SC_, *V*_OC_ and ff were all affected by changes in the I_2_ concentration, irrespective of the IL. After screening a range of *N*-alkyl substituted imidazolium-based ILs with chains of lengths from methyl to *n*-hexyl, it was found that DMII and EMII (Scheme 26) were the most beneficial additives. These, in combination with low initial I_2_ concentrations led to the lowest values of the transport resistance in the semiconductor. Too high I_2_ concentrations resulted in lower values of the diffusion resistance in the electrolyte and the counter electrode resistance. By using an optimized electrolyte composition of LiI (0.18 M), I_2_ (0.10 M) and DMII (0.60 M) in MPN it was possible to achieve PCEs of 0.65 ± 0.02% (Tables 10 and 11) for masked DSCs with the dye 72 in the presence of the co-adsorbent cheno; cheno was added to the dye in a single dye-bath. The reproducibility of the cells in Table 11 is noteworthy, and provides credibility to the improvement in DSC performance as a consequence of electrolyte tuning.^275^ Systematic changes in the electrolyte composition have taken account of the concentrations of I_2_, Li and IL, as well as the counter ion in the IL. The variation in values of *J*_SC_ and *V*_OC_ is substantial, and for dye 72 with cheno, some of the best performing DSCs were achieved with an electrolyte composition of LiI (0.18 M), I_2_ (0.05 M) and DMPII (0.60 M) in MPN; the best masked DSC exhibited values of *J*_SC_ = 3.27 mA cm^−2^, *V*_OC_ = 348 mV, *η* = 0.66% (11.8% with respect to a reference N719 cell). In all cases, data extracted from electrochemical impedance spectra support the trends in *J*_SC_ and *V*_OC_.^276^ A trade-off between values of *J*_SC_ and *V*_OC_ is often problematical when optimizing DSC performances for a given dye, and this is apparent when comparing entries 3 and 4 in Table 10. Both we^274^ and Marchini *et al.*^277^ have also observed that the addition of TBP is detrimental to DSC performance when the dye is 72.

**Table 11:** DSC parameters for fully-masked cells, fabricated under the same conditions and sensitized with the NHC iron(ii) dye 72 compared with N719.^a^ Electrolyte composition: LiI (0.18 M), I_2_ (0.10 M) and DMII (0.60 M) in MPN; the co-adsorbent cheno was added to the dye. Light illumination = 1000 W m^−2^. Average values are given in Table 10 for comparisons with other works

DSC number	*J*_SC_/mA cm^−2^	*V*_OC_/mV	ff/%	*η*/%	Relative *η*/%^a^
1	3.94	282	61	0.67	10.8
2	3.70	285	60	0.64	10.3
3	3.93	275	58	0.62	10.0
4	3.70	296	61	0.67	10.8

a Relative to an average N719 efficiency of 6.19% from three cells sensitized with N719 (6.22, 6.21 and 6.14%).

Marchini *et al.* continued the focus on the homoleptic dye 72 with an informative study that looked at the effects of a change from a TiO_2_ to SnO_2_ semiconductor, and of further electrolyte tuning.^277^ TiO_2_ remains the favoured photoanode material. When adsorbed on either SnO_2_ or TiO_2_, the NHC-dye 72 gives rise to photoinduced injection leading to a long-lived (millisecond regime) charge-separated state, but for SnO_2_, recombination is faster than for TiO_2_ leading to poorer DSC performance for SnO_2_. The PCE of DSCs with 72 (with cheno) can be pushed towards 1% by using (i) an electrolyte composition of Li (0.1 M), I_2_ (0.1 M), MgI_2_ (0.1 M), GNCS (0.1 M) and PMII (Scheme 26, (0.6 M)) in MeCN and (ii) TiO_2_ electrodes with a blocking underlayer made by spin coating Ti(O^i^Pr)_4_ on top of the FTO. Under these conditions, values of *J*_SC_ = 3.30 mA cm^−2^, *V*_OC_ = 440 mV, ff = 63% and *η* = 0.92% were obtained (Table 10), although there is no comment about masking the DSCs in the original publication.^277^

An noted earlier, NHC iron(ii) dyes have invariably been combined with the I_3_^−^/I^−^ redox mediator. In 2021, Wärnmark and coworkers reported that use of the [Co(bpy)_3_]^3+^/[Co(bpy)_3_]^2+^ redox mediator with dye 72 leads to a drastic decrease in *J*_SC_ (<0.2 mA cm^−2^), most probably due to significantly greater recombination of electrons with the oxidized form of the redox couple in the case of cobalt.^271^

### Improving the performances of DSCs with NHC iron(ii) dyes: heteroleptic complexes

**Scheme 27:**
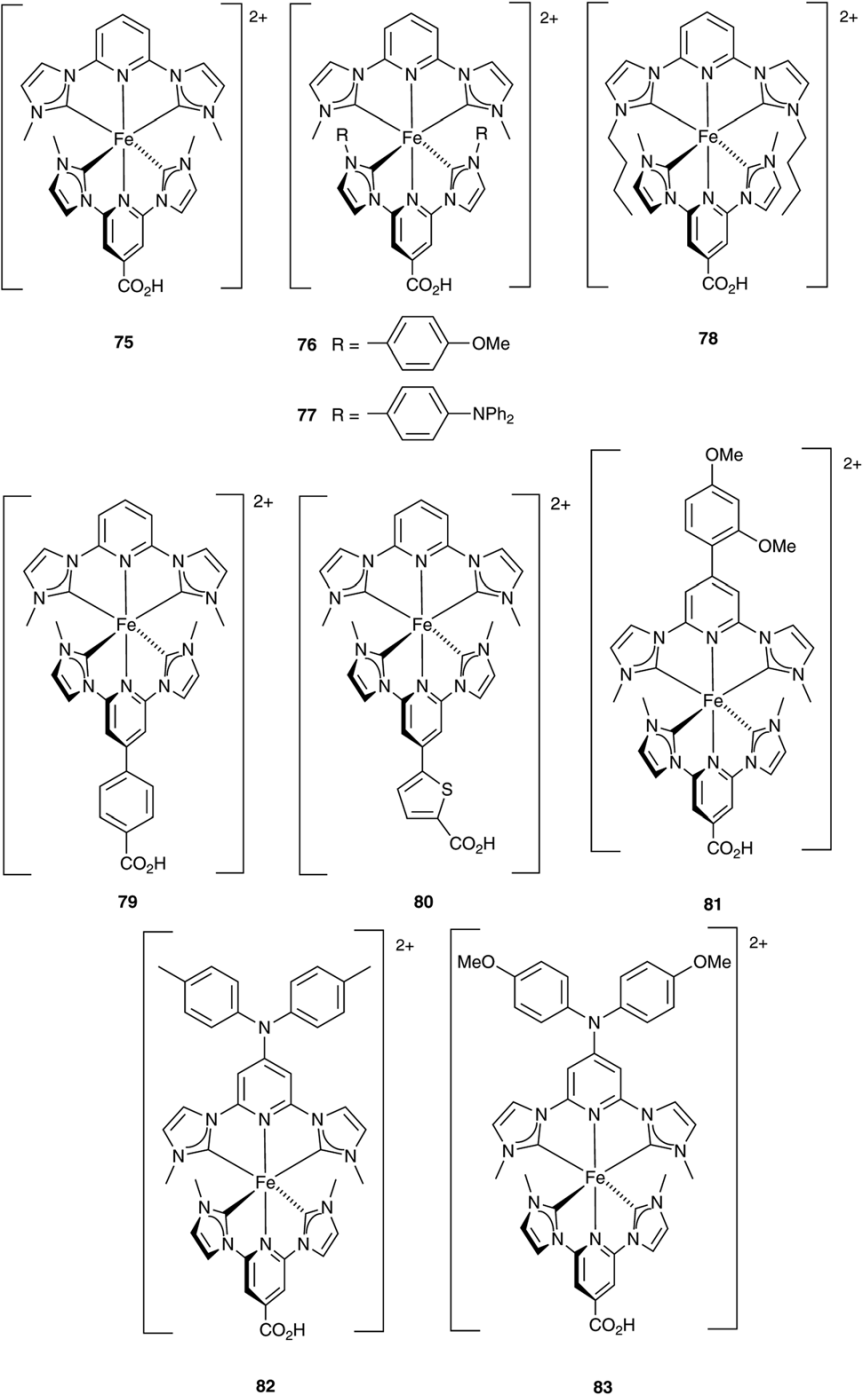
Structures of the heteroleptic NHC iron(ii) complexes 75–83.

Homoleptic NHC iron(ii) dyes like 72 lack the ‘push–pull’ design that is fundamental for optimal dye performance (see the discussion accompanying Fig. 8). In 2016, Pastore *et al.* reported the preparation and characterization of the three heteroleptic dyes 75–77 (Scheme 27). Compared to the homoleptic dye 72, 75–77 exhibited better interfacial charge separation. However, DSCs sensitized with 75, 76 or 77 with cheno as a co-adsorbent performed less well than a cell with homoleptic dye 72 (Table 10). The low values of *J*_SC_ were a contributing factor, and the data imply an unfavorable balance between electron injection and recombination processes. Pastore *et al.* drew several important conclusions which emphasized the challenges in developing NHC iron(ii) dyes: (i) fast recombination processes both with the oxidized dye and the I_3_^−^/I^−^ redox shuttle militate against good DSC performances, (ii) homoleptic NHC iron(ii) complexes do not exhibit the necessary directional electron flow towards the dye–semiconductor interface, and (iii) although heteroleptic NHC iron(ii) complexes exhibit beneficial interfacial charge separation, they do not show efficient rates of electron injection.^278^ However, a change in electrolyte composition as shown in Table 10, combined with the use of an under blocking layer on the photoanode and modifications of the counter electrode (PEDOT or Pt, Table 10), have a remarkable effect on the values of *J*_SC_ and, therefore, on photoconversion efficiency. For masked DSCs, the values of *η* = 1.29 and 1.44% are some of the highest recorded for NHC iron(ii) sensitizers. A critical factor was the addition of Mg^2+^ ions to the electrolyte. Computational modelling of the dye/TiO_2_ interface and calculations of the electron injection/recombination revealed that the presence of Mg^2+^ ions adsorbed on the semiconductor surface leads to a higher rate of electron injection for dye 75.^279^

Our own investigations of electrolyte tuning for homoleptic dyes (see above) concluded that an optimum composition was LiI (0.18 M), I_2_ (0.05 M) and DMPII (0.60 M) in MPN, and we maintained this composition for initial screening of heteroleptic dyes. Two factors which contributed to enhanced DSC performance were the time in which the photoanode was immersed in the dye bath, and the introduction of longer alkyl substituents in the ancillary ligand. Dye 78 (Scheme 27) was designed with *n*-butyl and methyl substituents on the ancillary and anchoring ligands, respectively, and after optimization of dye-bath conditions, the range of PCE values for three DSCs with dye 78 was 0.93–0.95% which represented 14.6–14.9% relative to DSCs sensitized with N719. The parameters in Table 10 are the average values for three cells. Interestingly, the performances of DSCs with 78 were lower when the co-adsorbent cheno was added to the dye bath (Table 10),^280^ and this observation appears to be consistent with the competition between dye and cheno reported by Lindh *et al.*^271^ However, the electrochemical impedance spectroscopic response was more strongly influenced by the immersion time in the dye bath than by the addition of cheno.^280^

Earlier discussions in this review illustrated how the nature of the anchoring ligand affects dye performance. This aspect of NHC iron(ii) dyes has only recently been explored with a comparison of the performances of sensitizers 75, 79 and 80 (Scheme 27).^279^ We have already noted that the performance of 75 (in the presence of cheno) is markedly enhanced by the addition of Mg^2+^ ions to the electrolyte (Table 10), the presence of an under blocking layer on the photoanode, and the use of Pt rather than PEDOT-coated counter electrodes. Employing similar fabrication conditions to DSCs with 79 and 80 leads to the DSC performances shown in Table 10. Although the introduction of the phenylene or thienyl spacer in 79 and 80 results in enhanced light absorption in the visible region, the dye performances were not significantly affected.^279^

A recent investigation from Gros and coworkers focused on heteroleptic dyes based upon 75 (Scheme 27) and bearing different substituents in the 4-position of the pyridine ring of the ancillary ligand.^281^ Substituents with varying electronic properties were selected and the most efficient dye was 81 (Scheme 27). DSCs were fabricated with 20 μm thick TiO_2_ photoanodes, and the optimized electrolyte composition described above, *i.e.* LiI (0.1 M), I_2_ (0.1 M), PMII (0.60 M), MgI_2_ (0.1 M), Bu_4_NI (0.1 M) and GNCS (0.1 M) in MeCN; the counter electrode was coated with PEDOT rather than Pt,^281^ even though in a previous study, Pt was shown to be more beneficial than PEDOT.^279^ The best performance was found for dye 81 which incorporates electron-donating MeO groups (Scheme 27 and Table 10). From the results of EIS studies and computational modelling, it was concluded that 81 combines good light-harvesting and beneficial recombination kinetics.^281^ Wärnmark and coworkers have recently reported the photophysical and electrochemical properties and DSC performances of heteroleptic dyes 82 and 83 (Scheme 27) which incorporate electron-donating ancillary ligands. Time-resolved spectroscopy confirmed ultrafast (<100 fs) interfacial electron injection from dyes 82 and 83 into TiO_2_. However, charge recombination between the injected electrons and the oxidized dye occurs rapidly, and only 5–10% of the injected electrons contribute to DSC current. Given this difficulty, it is exceptionally promising that PCEs of 1.31% for 82 and 0.93% for 83 were achieved (Table 10). For both dyes, cheno was added as a co-adsorbent, but since competitive anchoring of cheno and the dyes to the photoanode was observed, Wärnmark and coworkers used a sequential treatment of the surface with 82 or 83 followed by cheno. The DSC parameters for 82 and 83 in Table 10 are the best cells from sets of four for which performances were reproducible.^271^

In summary, the last few years have witnessed significant progress in the application of NHC iron(ii) sensitizers in DSCs. The range of NHC iron(ii) complexes remains limited, but the transition from homoleptic to heteroleptic dyes has now been made, providing synthetic strategies that can be adapted to diversify the families of dyes. The steadily increasing PCEs have been a result of (i) systematic tuning of electrolyte compositions, (ii) modification of electrode materials, (iii) ligand functionalization, and (iv) use of the co-adsorbent cheno, although competition between cheno and CO_2_H-anchored dyes must be considered. The field is ripe for development.

## Other first row transition metals in DSCs: still capitalizing on MLCT transitions

### Vanadium and chromium

To date, no vanadium-based complexes have been used as sensitizers in DSCs. Chromium compounds have also received little attention. Perhaps the most promising candidates for future investigations are octahedral chromium(0) complexes incorporating chelating diisocyanide ligands. These complexes have been developed by Wenger and coworkers and are isoelectronic with [Fe(bpy)_3_]^2+^. They are strong reductants and can exhibit long-lived ^3^MLCT excited-state lifetimes.^282–285^ To date, these species have not been tested in DSCs.

### Nickel

The most important use of nickel in DSCs is in the form of NiO as a p-type semiconductor in p-type or tandem DSCs.^286–293^ In 2012, we overviewed Ni(ii)-containing sensitizers for n-type DSCs that had been reported up to that date,^175^ and here we focus on Ni(ii) dyes reported since 2012. A number of dithiolate and dithiocarbamate complexes of nickel and bearing peripheral ferrocenyl units were described earlier in this review.^254–256^

**Fig. 14:**
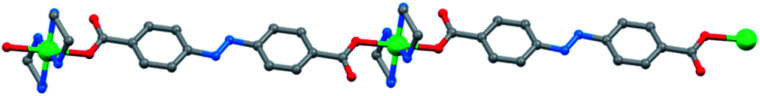
Part of the 1D-coordination polymer [Ni(en)_2_(azobc)]_*n*_ (CSD refcode SIQNEN01).

Zhang *et al.* reported the formation and structure of the 1D-coordination polymer [Ni(en)_2_(azobc)]_*n*_ (H_2_azobc = azobenzene-4,4′-dicarboxylic acid) (Fig. 14). FTO/TiO_2_ photoanodes were prepared with this dye and N719 as co-sensitizers. Although solution and solid-state UV-VIS spectra are reported, the integrity of the coordination polymer in solution was not established. Compared to N719 alone, additional light-harvesting towards the UV region is observed when a combination of dyes was used, and a significantly enhanced *J*_SC_ (13.46 mA cm^−2^*vs.* 8.78 mA cm^−2^) was reported. An increase in *V*_OC_ suggested that co-sensitization produced an upward shift of the conduction band edge of the TiO_2_ semiconductor.^294^

While nickel(ii) phthalocyanine dyes in DSCs have been investigated, their performances are typically lower than cells sensitized by their zinc(ii) analogues,^57^ as exemplified in the work of Gorduk *et al.*^226,227^

## Zinc(ii) as ‘glue’: ligand-centred chromophores rather than MLCT transitions

Since Zn(ii) possesses a d^10^ configuration, zinc-based dyes rely not on MLCT transitions but on ligand-centred chromophores. The most investigated zinc(ii)-containing sensitizers in DSCs are zinc(ii) porphyrinato and phthalocyanato complexes and their structures and applications in DSCs have been thoroughly reviewed.^46,48,53–57^ In this section, we focus upon complexes in which the Zn^2+^ ion functions as the ‘glue’ that connects anchoring and ancillary ligand domains. We also look at a number of zinc(ii) compounds that have been used as co-sensitizers, *e.g.* with N719. As in previous sections, our main focus is on post-2012 publications; for earlier work, we refer the reader to our 2013 review.^175^

### Zinc(ii) dyes assembled *in situ* using the SALSAC strategy

**Fig. 15:**
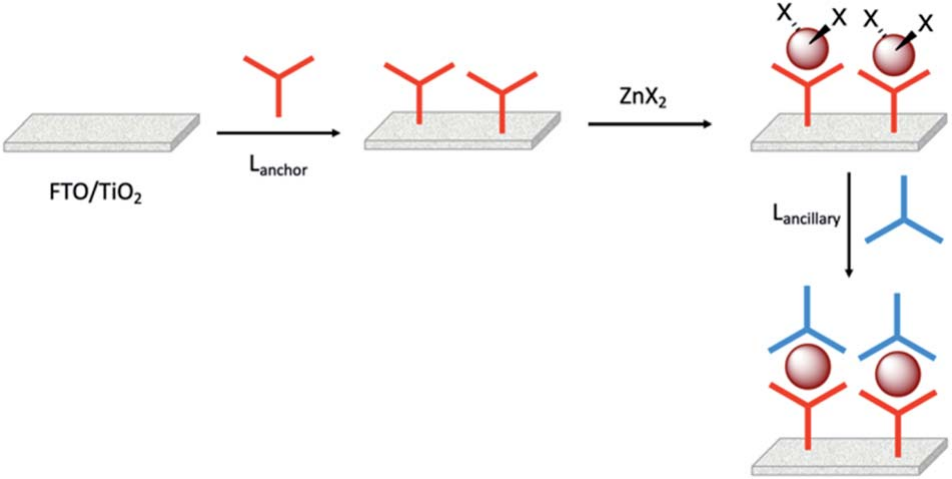
The SALSAC approach to *in situ* assembly of a heteroleptic bis(terpyridine)zinc(ii) dye on an electrode surface using a stepwise strategy. ZnX_2_ is typically Zn(OAc)_2_ or ZnCl_2_.

**Scheme 28:**
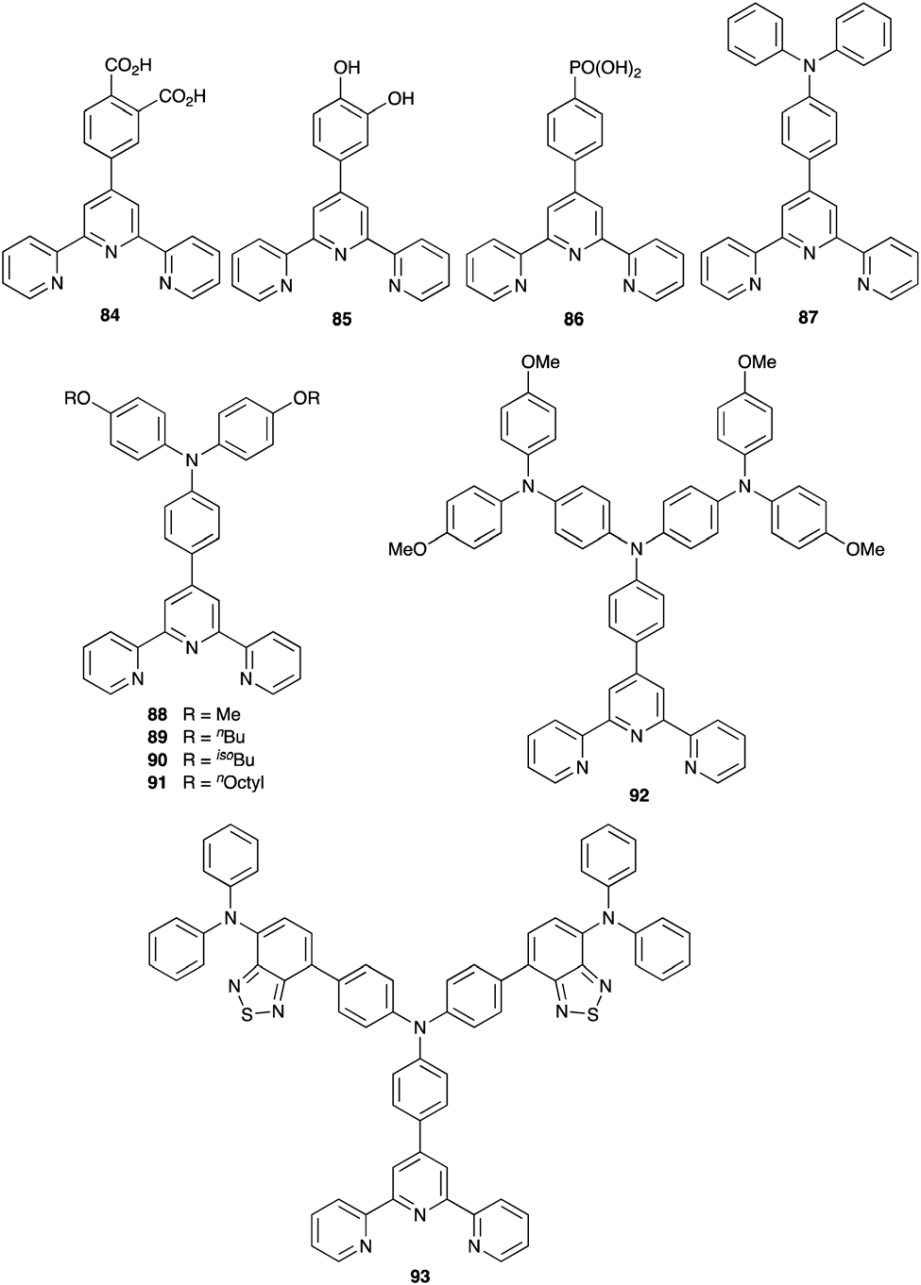
Structures of derivatives of tpy used as anchoring (84–86) and ancillary (87–93) ligands in zinc(ii) dyes.

As detailed earlier, the SALSAC approach to assembling copper(i) sensitizers on TiO_2_ (Fig. 9) is extremely beneficial for effective screening of copper-based dyes. Like Zn(ii), Cu(i) has a d^10^ electronic configuration and the ligands in tetrahedral [Cu(N^N)_2_]^+^ complexes (N^N = diimine) are labile in solution. Ligand exchange between two different homoleptic [Cu(N^N)_2_]^+^ species occurs immediately in solution to give a statistical mixture of homoleptic and heteroleptic complexes.^177^ In contrast, octahedral [Zn(tpy)_2_]^2+^ (tpy = 2,2′:6′,2′′-terpyridine or a substituted derivative) complexes undergo slow exchange of ligands in solution. Thus, although the SALSAC approach as shown in Fig. 9 can be applied to assemble [Zn(tpy)_2_]^2+^-based dyes on TiO_2_, the procedure has been adapted to a stepwise assembly (Fig. 15) to allow optimal formation of anchored heteroleptic [Zn(L_anchor_)(L_ancillary_)]^2+^ complexes.^295^ In an initial investigation, FTO/TiO_2_ electrodes were functionalized with anchoring ligands 84, 85 or 86 (Scheme 28), followed by treatment with Zn(OAc)_2_ or ZnCl_2_, and then ancillary ligands 87 or 88 (Scheme 28). Combinations of these anchoring and ancillary ligands produced TiO_2_-anchored zinc(ii) dyes with solid-state absorption spectra having maxima in the range 425–480 nm arising from ILCT. DSCs using [Zn(84)(87)]^2+^ in combination with an I_3_^−^/I^−^ redox mediator performed the best of the series, but all efficiencies were very low (*η* < 1%). Nonetheless, this study confirmed the potential for using the SALSAC strategy to assemble zinc(ii) sensitizers on TiO_2_ photoanodes.^295^ An extension of this work led to the screening of DSCs with dyes [Zn(L_anchor_)(L_ancillary_)]^2+^ in which L_anchor_ = 84 or 86 and L_ancillary_ = 88–91 (Scheme 28). Solid-state absorption spectra for the surface-bound dyes exhibited broad maxima (*λ*_max_ = 443–448 nm) assigned to ILCT. Despite the donor–acceptor nature of the complexes and the modifications of the alkyloxy units on the ancillary ligands, masked DSCs containing the dyes [Zn(84)(88)]^2+^, [Zn(84)(89)]^2+^, [Zn(84)(90)]^2+^, [Zn(84)(91)]^2+^, [Zn(86)(88)]^2+^, [Zn(86)(89)]^2+^, [Zn(86)(90)]^2+^ and [Zn(84)(91)]^2+^ in conjunction with an I_3_^−^/I^−^ redox shuttle exhibited poor PCEs, the primary reason being extremely low values of *J*_SC_ (<0.5 mA cm^−2^).^296^ An important point to note is that DSCs containing FTO/TiO_2_ photoanodes *without any adsorbed dye* generate small values of *J*_SC_ and *V*_OC_ which contribute appreciably to parameters for poorly performing dyes.^297^ In order to enhance light-harvesting of the [Zn(L_anchor_)(L_ancillary_)]^2+^ complexes, ancillary ligands 92 and 93 (Scheme 28) with extended chromophores were incorporated into the dyes [Zn(84)(92)]^2+^, [Zn(84)(93)]^2+^, [Zn(86)(92)]^2+^ and [Zn(86)(93)]^2+^. Masked DSCs with these dyes and an I_3_^−^/I^−^ redox shuttle still performed poorly under light intensity of 1000 W m^−2^ with *J*_SC_ < 1 mA cm^−2^ and *V*_OC_ ≈ 400 mV, although good ff values were achieved. Moving the electron-withdrawing thiadiazole units from the ancillary to the anchoring domain could be beneficial.^297^ This series of studies indicates that dyes based upon the {Zn(tpy)_2_}^2+^ core are not optimal for sensitizers in DSCs, and no further investigations in this area have been reported.

### Other zinc(ii) sensitizers and co-sensitizers

**Scheme 29:**
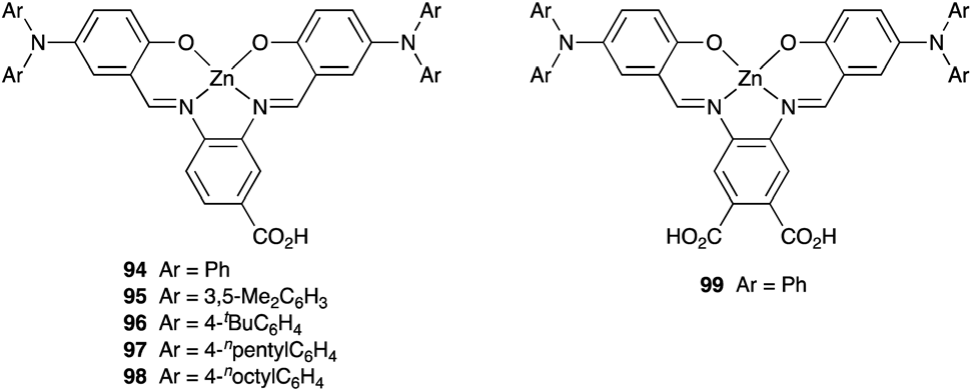
Schiff base zinc(ii) D–π–A dyes.

Jing and coworkers designed the D–π–A zinc(ii) complexes shown in Scheme 29. DSCs were made by combining FTO/TiO_2_/dye photoanodes and an I_3_^−^/I^−^ redox mediator and were illuminated under a light intensity of 1000 W m^−2^. The introduction of the second anchoring group on going from 94 to 99 was beneficial both for *J*_SC_ and *V*_OC_. Taking into account that there is no comment about the masking of the DSCs in the original paper,^298^ their performances appear to be comparable with those of the bis(terpyridine)zinc(ii) dyes described above.

**Scheme 30:**
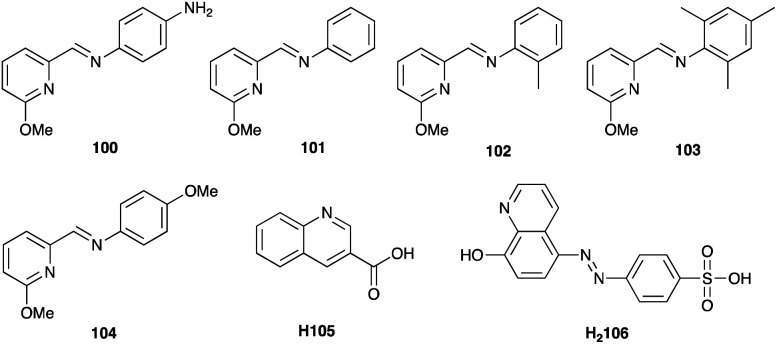
Structures of ligands 100–104, H105 and H_2_106 used in zinc(ii) co-sensitizers with N719.

There has been a number of investigations dealing with the use of Schiff base zinc(ii) dyes as co-sensitizers with the ruthenium(ii) dye N719, the aim of enhancing light absorption in the blue-violet and UV regions. This could potentially offset visible light absorption by I_3_^−^ that competes with light-harvesting by the dye. In 2014, Yang and coworkers prepared [Zn(100)Cl_2_]; ligand 100 is shown in Scheme 30. Co-sensitized DSCs were fabricated by immersing FTO/TiO_2_ electrodes in an EtOH solution of [Zn(100)Cl_2_] followed by an EtOH solution of N719. It is not apparent how [Zn(100)Cl_2_] anchors to the semiconductor. Open (*i.e.* unsealed) cells contained an I_3_^−^/I^−^ redox mediator along with TBP in an MeCN–propylene carbonate electrolyte. The results indicated that the presence of the zinc(ii) complex boosts values of *J*_SC_, leading to a small increase in PCE.^299^ This was followed by screening of [Zn(101)Cl_2_], [Zn(102)Cl_2_] and [Zn(103)Cl_2_] (see Scheme 30 for 101–103) as co-sensitizers with N719. Masked DSCs were assembled in a similar manner to those with [Zn(100)Cl_2_]/N719 and the light intensity was 1000 W m^−2^. From the absorption spectra of the complexes compared to that of N719, it was concluded that use of [Zn(103)Cl_2_] as a co-sensitizer would ‘fill-in’ the higher energy part of solar light harvested by the DSC. Indeed, values of *J*_SC_ increased from 13.26 to 17.36 mA cm^−2^ leading to an increase in PCE from 5.14 to 6.62%; *V*_OC_ was little affected.^300^ Yang and coworkers also demonstrated that the introduction of a peripheral electron-donating MeO substituent to give ligand 104 (Scheme 30) is beneficial in co-sensitized DSCs with dyes N719 and 104. EIS measurements show that the use of the zinc(ii) co-sensitizer led to a decrease of the electron transfer impedance and an increase in the rate of charge transfer.^301^ Yang, Fan and coworkers have investigated the use of [Zn(105)_2_] (H105 = quinoline-3-carboxylic acid, Scheme 30) as a co-sensitizer with N719. In the solid state, the zinc(ii) complex forms a coordination network [Zn(105)_2_]_*n*_, and the material is soluble in polar solvents including DMSO, EtOH and MeOH. The species present in EtOH solution, formulated here as [Zn(105)_2_], has a broad absorption maximum at 392 nm. Photoanodes were co-sensitized with [Zn(105)_2_] and N719 by sequential dipping in dye baths of single dyes (Zn(ii) then N719). Open (*i.e.* unsealed) DSCs with the I_3_^−^/I^−^ redox shuttle and irradiated under a light intensity of 1000 W m^−2^ gave enhanced values of *J*_SC_ and *V*_OC_ with respect to DSCs containing only N719 (*J*_SC_ = 18.61 *vs.* 13.42 mA cm^−2^ and *V*_OC_ = 730 *vs.* 660 mV). EIS data revealed that the electron lifetime for the co-sensitized DSC was longer (16.42 ms) than for the N719 cell (8.59 ms).^302^ Interestingly, use of the zinc(ii) complex of ligand [106]^2−^ (Scheme 30) as a co-sensitizer with N791 leads to an increase in *J*_SC_ but a decrease in *V*_OC_. Again, the characterized complex was a coordination polymer, [Zn_3_(106)_2_(OH)_2_(OH_2_)_6_]_*n*_,^303^ and we note that dissolution in EtOH for the dye-bath and photoanode assembly will result in disassembly of the polymer.

## Recyclability

This review has focused on harvesting energy from a sustainable source, the Sun, using DSCs with sustainable components. However, the process of photoconversion is only truly sustainable if materials in the DSC can be recycled and/or the device can be regenerated after degradation processes result in its failure. This aspect of the end-of-life processing of DSCs is not widely investigated, with most effort being invested on the re-use of TiO_2_ or reclamation of platinum group metals. Most recently, electrolyte recycling has become of interest, with suggestions that copper-based electrolytes might be more recyclable and sustainable than those based on iodine.

A common degradation process in a DSC is the loss of volatile solvent from the electrolyte and extending the lifetime of the device can be addressed by replacing the organic solvent by a less volatile medium. The pros and cons of aqueous-based DSCs have been critically reviewed by Bella, Grätzel and coworkers.^304^ An alternative approach is the use of solid-state, ionic liquid or gel electrolytes.^44,305,306^

Typical DSCs used in the research laboratory comprise glass/FTO/TiO_2_ and glass/Pt electrodes which are only *ca.* 2 cm × 2 cm in size (Fig. 2c). Upscaling to commercial needs demands significant changes to design, for example the development of flexible devices with polymer substrates for roll-to-roll manufacturing. The question of the recyclability of DSC components has been addressed in several recent reviews,^307–309^ and the recovery of silver from silicon photovoltaics^310^ is also relevant for the silver employed in electrical contacts in DSCs.

Bonomo and coworkers highlight several points which are fundamental for approaches to recycling.^309^ The first is the use of critical raw materials *e.g.* Ru, Co, Ag and Pt, and we have addressed this issue throughout this review. The second is performance degradation arising from electrolyte instability, mainly linked to loss of volatile solvents (see above). The third point concerns the high energy-demanding conducting glass substrates.^309^ While recycling of FTO or other transparent conducting oxide (TCO) glass from DSCs may be technically achievable, it is not perceived to be commercially viable in practice.^307^ Bonomo and coworkers also comment on the sustainability associated with waste management. They also note that, based upon the quantities of materials used within a DSC, an assessment of sustainability factors should focus more on counter electrode and electrolyte materials than on dyes.^309^

## Conclusions

DSCs are now an established technology and are destined to contribute to the sustainable energy market. Ideally, all device components should be based upon sustainable materials, and the use of TiO_2_ as the semiconductor on the photoanode is in line with these criteria. The use of metal-free (organic dyes) including natural dyes is also beneficial and indeed, state-of-the-art DSCs including certified devices are all based on organic dyes. From the point of view of sustainability, organic dyes provide the most efficient and stable DSCs. The emphasis of this review has been on metal-containing dyes, of which the best performing DSCs include those containing ruthenium(ii) sensitizers. However, ruthenium is poorly abundant in the Earth's crust. Although the dye is a critical component of a DSC, the photoconversion efficiency is also dictated by the complementarity of the dye and the redox mediator. Combined with organic, zinc(ii) porphyrin, zinc(ii) phthalocyanine and ruthenium(ii) dyes, one of the most established redox mediators is the I_3_^−^/I^−^ couple. However, its use limits the values of *V*_OC_ that can be reached and it creates a corrosive chemical environment within the DSC which impacts upon the long-term stability of the cells. With these specifics as a starting point, we have presented developments in the field of DSCs containing dyes and redox mediators based upon coordination compounds of first row d-block metal ions. The focus has been primarily on progress over the last decade.

First row d-block metal coordination compounds, especially those containing cobalt(iii)/cobalt(ii), copper(ii)/copper(i) and iron(iii)/iron(ii) couples, have come to the fore as alternative redox mediators to I_3_^−^/I^−^. In contrast to the I_3_^−^/I^−^ couple, the redox potentials of couples based on metal complexes can readily be tuned by use of different ligands. Of particular importance is the fact that use of Cu^2+^/Cu^+^ redox mediators (which are typically bis(diimine) complexes) has led to *V*_OC_ values in excess of 1000 mV. This is an exciting development which contributes to higher PCEs. However, the flattening of the copper(i) tetrahedral geometry upon oxidation leads to the copper(ii) state being susceptible to attack by Lewis bases. This includes TBP which is often added to electrolyte solutions to produce a shift in the conduction band of the semiconductor. The effects of TBP in Cu^2+^/Cu^+^-containing electrolytes remain to be fully understood.

Turning to the dye and the fact that ruthenium is a critical raw material, we focused in this review on the use of coordination complexes of the more abundant first row d-block metals, in particular copper, iron and zinc, as dyes in DSCs. A major challenge in these DSCs is an enhancement of their photoconversion efficiencies which currently lag significantly behind those containing ruthenium-based dyes. Bis(diimine)copper(i) sensitizers exhibit promising PCEs, especially when used as a co-sensitizer with organic dyes such as SQ2, their light-harvesting ranges being complementary (Fig. 10). Both the SALSAC and HETPHEN strategies militate against ligand redistribution that is an inherent problem of bis(diimine)copper(i) complexes in solution. The crustal abundance of iron makes this the dream element on which to base a sensitizer, and the discovery of the long excited-state lifetimes of N-heterocyclic carbene iron(ii) complexes has, in the last few years, opened up an exciting field of iron-based dyes. The steadily increasing PCEs of DSCs incorporating NHC iron(ii) dyes have been achieved by systematic tuning of electrolyte compositions, modification of electrode materials, and ligand functionalization. Although the PCEs of these DSCs are only now passing 1%, the rapid improvements in the last few years confirm that this field is ripe for development.

If the use of DSCs on a commercial scale is to become a truly sustainable endeavour, either the device components must be recycled and/or it must be possible to revive spent devices. These aspects of sustainable DSCs have been reviewed by others and we draw attention to these publications to highlight the needs for the future.

## Author contributions

Conceptualization: CEH and ECC; writing – original draft: CEH; writing – review and editing: ECC.

## Conflicts of interest

There are no conflicts to declare.

## Supplementary Material
